# Computational investigation and experimental validation of the molecular mechanism of *Solanecio mannii* aqueous roots extract against cervical cancer

**DOI:** 10.1371/journal.pone.0323680

**Published:** 2025-05-30

**Authors:** Amel Elbasyouni, Mutinda C. Kyama, Hany A. El-Shemy, Peter G. Mwitari

**Affiliations:** 1 Molecular Biology and Biotechnology Program, Pan African University Institute for Basic Sciences, Technology & Innovation (PAUSTI), Nairobi, Kenya; 2 Department of Medical Laboratory Science, College of Health Sciences, Jomo Kenyatta University of Agriculture and Technology, Nairobi, Kenya; 3 Biochemistry Department, Faculty of Agriculture, Cairo University, Giza, Egypt; 4 Centre for Traditional Medicine and Drug Research, Kenya Medical Research Institute (KEMRI), Nairobi, Kenya; Institute of Medical Sciences, Banaras Hindu University, INDIA

## Abstract

Cervical cancer remains one of the leading causes of cancer-related mortality among women worldwide, particularly in low- and middle-income countries, highlighting the need for improved strategies in treatment and management. This study aimed to investigate the anti-cervical cancer potential and molecular mechanisms of *Solanecio mannii* (*S. mannii*) aqueous extract using a “multi-compound, multi-target, multi-pathway” approach, integrating both computational and experimental methods. The metabolomics profile of the extract was analysed, and its selective cytotoxicity was assessed against human cervical cancer cell lines (HeLa cells) using the CCK8 assay. A network pharmacology approach identified potential molecular targets and pathways, which was complemented by molecular docking and dynamic simulation. The expression levels of key targets were validated experimentally using quantitative real-time polymerase chain reaction. Additionally, the extract’s effects on apoptosis, autophagy, and cell cycle progression were studied experimentally. The aqueous roots extract exhibited selective cytotoxicity against HeLa cells with an IC_50_ of 12.53 ± 4.983 μg/ml. The network pharmacology analysis identified 25 drug-like compounds targeting 493 unique cervical cancer-associated proteins, forming a protein-protein interaction network of 465 nodes and 2230 edges, and implicated in 178 enriched KEGG pathways. Key targets, including NFΚB1, PIK3CA, HIF1A, STAT3, HSP90AA1, HSP90AB1, PPARG, and ESR1 were experimentally downregulated. Furthermore, *S. mannii* aqueous roots extract triggered apoptosis through endoplasmic reticulum stress, DNA damage, and activation of the non-transcriptional, P53-mediated mitochondrial apoptotic pathway. Additionally, the extract inhibited hypoxia and autophagy, and induced cell cycle arrest at the G2/M phase, even in the presence of oncogenic HPV proteins (E6 and E7). In conclusion, *Solanecio mannii* aqueous roots extract demonstrates a “multi-compound, multi-target, multi-pathway” molecular mechanism against cervical cancer.

## Introduction

Cervical cancer incidence is increasing globally, particularly in low and middle-income countries [1]. It is the most common cancer in women in sub-Saharan Africa and the second most common cancer in Northern Africa after breast cancer. According to GLOBOCAN 2020, there were 604,127 new cases and 341,831 fatalities worldwide [2]. Cervical cancer is the leading cause of cancer-related death among females in Eastern Africa [3]. Age-adjusted rates of 40, 36, and 31 cases per 100,000 woman-years were recorded in Eastern, Southern, and Central Africa. Malawi, Gambia, Kenya, Uganda, and Zimbabwe have the highest rates of both incidence and death [2,4,5]. Swaziland has the highest incidence rate in Africa and the world [4]. Zimbabwe’s incidence rate has dramatically increased by 2.5-fold [5]. Transitioning countries have higher rates overall due to socioeconomic status, higher poverty rates, and limited access to HPV vaccination [2]. Globally, cervical cancer prevalence is expected to rise significantly in 2040 [3]. Notably, more than 90% of cervical cancer occurs after 20 years post-infection with high-risk oncogenic Human papillomaviruses (HPV) strains, making it a “necessary causal agent”. Within 12–24 months of exposure to the virus, 90% of HPV infections are cleared or become inactive (transitory infection) [6]. However, infections by the high-risk HPV types persist, increasing the risk of progression to cervical cancer. Infections with HPV 16 and 18 are associated with 50–70% and 20–30% of this type of cancer, respectively [7]. From a total of 2.2 million new cancer cases attributed to viral infections, 570,000 new cervical cancer cases are attributed to HPV persistent infection [8].

To effectively address the high occurrence of cervical cancer, it is necessary to go beyond simply managing symptoms or focusing solely on specific signalling pathways and molecular mechanisms through targeted or immunological therapy. Current cervical cancer treatments now available cause significant adverse effects and contribute to the emergence of drug resistance in tumours [9]. Consequently, there is a pressing requirement for novel and enhanced therapeutic alternatives that are more effective. Phytopharmaceutical drug discovery can accelerate and streamline the development of pharmaceuticals, by potentially influencing essential signalling pathways and improving the patient’s quality of life. phytopharmaceutical drug discovery may accelerate and reduce the cost of medication development [10]. Certain compounds in plants and multi-component extracts can prevent cancer cell growth, through triggering programmed cell death, cell growth inhibition, and cell cycle arrest [11]. Compounds and multi-component plant extracts can improve the quality of life for cancer patients [12]. Network pharmacology is a holistic and systems-level approach that integrates pharmacology with network biology to understand the complex interactions between drugs and biological systems. This approach is particularly valuable in the study of multi-component plant extracts, as it allows for the mapping of interactions between the diverse constituents of the extracts and their multiple molecular targets within cells. Thereby, it reveals the underlying mechanisms of action and identifies potential synergistic effects of the compounds. Ethnobotanical investigations have shown that the roots of *Solanecio mannii* (*S. mannii*) possess medicinal properties [13]. Traditionally, this plant has been used to cure wounds, infections, and cancer. Research attributes its efficacy to its rich phytochemical composition, which includes sesquiterpenoids, flavonoids, alkaloids, and phenolics. However, there is a significant lack of evidence on the detailed metabolomic profile and molecular mechanisms against cervical cancer. The current study aimed to investigate the molecular mechanism of metabolites derived from the aqueous extract of *Solanecio mannii* roots for the management of cervical cancer by integrating computational approaches (network pharmacology, molecular docking, and dynamic simulations), followed by experimental validation to forecast the “multi-component- multi target- multi pathway” mechanism of *S. mannii* against cervical cancer.

## Materials and methods

### Plant collection and extraction

Roots of *Solanecio mannii* were collected from Kenya (0°12′06.7″N 35°05′58.3″E), and identified at the National Museum of Kenya. No permits were required for the collection of plant material as the study site was not located within a protected area, and the species collected was not classified as endangered or restricted. The plant material was freeze-dried and ground before total solvent extraction using diethyl-ether: methanol (v/v). The total extract was filtered with Whatman filter paper 1 and concentrated at 55°C under vacuum. The total extract was rinsed with 250 ml n-hexane to separate non-polar residues. The lower phase was partitioned for 24 hours with distilled water: ethyl acetate (v/v) to separate mid-polar from highly polar phytocompounds. The highly polar aqueous extract in the lower phase was collected, filtered, and freeze-dried for further studies.

### Gas chromatography-mass spectrometry (GC-MS)-based metabolomics profiling of *S. mannii* aqueous roots extract

The metabolomics profile of the extract was analysed using gas chromatography coupled with mass spectrometry. The GC-MS analysis was conducted using an Agilent Technologies 7890B gas chromatograph, which was equipped with a DB-5MS column (30 m x 0.25 mm internal diameter and 0.25 µm film thickness), and a 5977A mass spectrometer. The injection volume was 1 µl, and hydrogen gas was employed as the carrier gas, flowing at 1.0 ml/min. The temperature was held at 60 °C for 1 minute, then increased to 320 °C at a rate of 10 °C per minute, and then maintained for an additional 10 minutes. The injector and detector were maintained at 300°C and 320°C, respectively. Using a solvent delay of 6 minutes and a spectral range of m/z 50–800, mass spectra were obtained through electron ionisation at 70 eV. The quadrupole and mass analyser were maintained at 150°C and 230°C, respectively. The spectrum fragmentation pattern was compared to those stored in the Wiley and NIST Mass Spectral Library data to identify phytocompounds. The sample was derivatized before GC-MS analysis. In brief, the dried aqueous extract was resuspended in 50 µ L of bis(trimethylsilyl)trifluoroacetamide (BSTFA), supplemented with trimethylchlorosilane (TMCS) (99:1) silylation reagent and 50 µ L of pyridine.

### Liquid chromatography-electrospray ionization-tandem mass spectrometry-based metabolomics profiling of *S. mannii* aqueous roots extract

Liquid chromatography-electrospray ionization-tandem mass spectrometry (LC-ESI-MS/MS) was used to analyse the material. An ExionLC AC system was used for separation, and a SCIEX Triple Quad 5500 + MS/MS system with an electrospray ionisation (ESI) detector was used for detection. The separation was performed with an Ascentis® Express 90 Å C18 Column (2.1 × 150 mm, 2.7 µm). Two eluents comprised the mobile phase; A: 5 mM ammonium formate (PH = 8) for the negative ionization mode or 5 mM ammonium formate, supplemented with 0.1% formic acid (PH = 3) for the positive ionization mode; and B: LC-grade acetonitrile.

With a flow rate of 0.3 ml/min and an injection volume of 5 µl, the mobile phase gradient was programmed as follow:

i. 5% B at 0–1 min,
ii. 5–100% B from 1–20 min,
iii. 100% B from 20–25 min,
iv. 5% at 25.01,
v. 5% from 25.01–30 min.

For MS/MS analysis, negative and positive ionization modes were applied with EMS-IDA-EPI scan from 100 to 1000 Da for MS1 with curtain gas of 25 psi, source temperature of 500°C, and ion source gas 1 and 2 were 45 psi. The ion spray voltages in the negative and the positive modes were -4500 and 5500, respectively. For MS2, the scan was set from 50 to 1000 Da with a de-clustering potential of -80 and 80, and collision energy of -35 and 35 for the negative and positive ionization modes, respectively. Identification of biomolecules was performed using the Respect Library database-implemented MS-DIAL version 4.70, with an MS tolerance of 0.2 Daltons for parent and fragment, and matching of 70%.

For the targeted metabolites profiling, a Multiple Reaction Monitoring technique (MRM) was used. Separation was performed using a Poroshell 120 EC-C18 column (3.0 × 100 mm, 2.7 µm). The mobile phases consisted of two eluents A: 0.1% formic acid in water; and B: acetonitrile. The mobile phase program is summarized in Table 1.

**Table 1:** Mobile phase program used for targeted metabolites profiling using multiple reaction monitoring technique (MRM) (injection volume: 5 µl).

Time (min)	%B	Flow rate (mL/min)
0	8	0.5
1	8	0.5
4	15	0.4
12	20	0.4
20	30	0.4
25	45	0.4
25.01	8	0.5
28	8	0.5

Negative ionization mode was applied with the following mass spectrometer parameters: curtain gas: 25 psi, Ion Spray voltage: -4500, source temperature: 400°C, and ion source gas: 55 psi. Table 2 shows the MRM parameters used for targeted metabolite profiling.

**Table 2:** Multiple reaction monitoring technique (MRM) parameters used for targeted metabolites profiling.

ID	Q1 (m/z)	Q3 (m/z)	RT (min)	CE (V)	CXP (V)	DP (V)
Gallic acid 168.9/124.9	168.9	124.9	1.67	-30	-11	-110
Gallic acid 168.9/79	168.9	79	1.67	-30	-11	-110
Caffeic acid 178/135	178.9	135	5.83	-22	-9	-115
Caffeic acid 178/107	178.9	107	5.83	-30	-7	-115
Rutin 609/299.9	609	299.9	9.13	-48	-15	-230
Rutin 609/270.9	609	270.9	9.13	-70	-9	-230
Coumaric acid 162.9/119	162.9	119	7.7	-20	-7	-90
Coumaric acid 162.9/93	162.9	93	7.7	-40	-5	-90
Vanillin 151/136	151	136	7.48	-12	-9	-140
Vanillin 151/92	151	92	7.48	-16	-7	-140
Naringenin 271/151	271	151	20.98	-24	-25	-130
Naringenin 271/119	271	119	20.98	-34	-11	-130
Querectin 301/151	301	151	18.16	-28	-9	-50
Querectin 301/178.8	301	178.8	18.16	-20	-7	-50
Ellagic acid 301/145	301	145	8.97	-40	-14	-120
Ellagic acid 301/245	301	245	8.97	-38	-14	-120
3.4-Dihydroxybenzoic acid 152.9/109	152.9	109	3.13	-40	-5	-75
3,4-Dihydroxybenzoic acid 152.9/90.9	152.9	90.9	3.13	-20	-7	-75
Hesperetin 301/164	301	164	22.62	-23	-10	-125
Hesperetin 301/136	301	136	22.62	-38	-10	-125
Cinnamic acid 146.9/102.6	146.9	102.6	18.16	-17	-6	-60
Cinnamic acid 146.9/77	146.9	77	18.16	-33	-6	-60
Methyl gallate 183/124	183	124	5.04	-30	-10	-110
Methyl gallate 183/140	183	140	5.04	-30	-10	-110
Kaempferol 284.7/93	284.7	93	22.08	-46	-10	-120
Kaempferol 284.7/116.8	284.7	116.8	22.08	-52	-10	-120
Ferulic acid 192.8/133.9	192.8	133.9	8.89	-16	-5	-25
Ferulic acid 192.8/177.9	192.8	177.9	8.89	-12	-5	-25
Syringic acid 196.9/122.8	196.9	122.8	6.23	-24	-5	-30
Syringic acid 196.8/181.9	196.9	181.9	6.23	-12	-5	-30
Apigenin 269/151	269	151	21.47	-15	-7	-35
Apigenin 269/117	269	117	21.47	-15	-7	-35
Catechin 288.8/244.9	288.8	244.9	5.05	-16	-8	-40
Catechin 288.8/109	288.8	109	5.05	-32	-8	-40
Luteolin 284.7/132.9	284.7	132.9	18.14	-38	-10	-50
Luteolin 284.7/150.9	284.7	150.9	18.14	-26	-10	-50
Daidzein 253/132	253	132	16.22	-55	-10	-65
Daidzein 253/91	253	91	16.22	-50	-13	-65
Chlorogenic acid 353/191	353	191	5.1	-23	-10	-60
Chlorogenic acid 353/179	353	179	5.1	-35	-10	-60

### Cytotoxicity analysis using Cell Counting Kit 8 (CCK8) assay

Kidney epithelial cells of African green monkey (Vero cells) and human cervical cancer cell lines (HeLa cells) were generously provided by Dr. Peter G. Mwitari, and were cultured in 10% foetal bovine serum (FBS) in Eagle’s Minimum Essential Medium (EMEM). The culture medium was supplemented with 1% streptomycin/penicillin, sodium bicarbonate, HEPES, and glutamine. The cells were inoculated into a 96-well plate, to attach overnight, at a density of 1 × 10^4^ cells/well. To prepare a stock of 100,000 µg/ml, 100 mg of dried extract was dissolved in 1 ml of absolute Di-Methyl Sulf-Oxide (DMSO). The stock solution was further diluted with complete growth media to achieve a working solution of 200µg/ml. Cells were treated with the aqueous extract at a concentration of 200 µg/ml for 48 hours (h). After the treatments, wells were rinsed with 100µl of phosphate-buffered saline (PBS). 90µl of complete growth media and 10µl of CCK8 solution were added to each well. Plates were incubated for 3 hours, after which absorbances were measured at 450 and 650 nm using a microplate reader. Positive and negative controls were rapamycin and 0.2% DMSO, respectively. The incubation parameters were set at 37°C, 5% CO2, and 95% humidity, throughout the study. Additionally, cells were treated with different concentrations of the extract, ranging from 300 µg/ml to 4.69 µg/ml for 48 h. The half-maximal inhibitory concentration (IC_50_) and cytotoxicity concentration (CC_50_) were determined using Splin/LOWESS fit in GraphPad Prism. Cell viability and selectivity were determined using the following formulas:


$$Cell\;viability\;(\;\%)=\frac{\left(absorbance\;of\;treated\;samples{\;OD}_{450}-\;absorbance\;of\;treated\;samples{\;OD}_{650}\;\right)-\;{Absanbance\;of\;blank\;OD}_{450}}{\left(absorbance\;of\;untreated\;samples{\;OD}_{450}-\;absorbance\;of\;untreated\;samples{\;OD}_{650}\;\right)-\;{Absanbance\;of\;blank\;OD}_{450}}$$

$$SI=\frac{CC50}{IC50}$$

### Network pharmacology study

#### Medicinal properties of the identified compounds.

Simplified Molecular Input Line Entry System (SMILES) of the compounds were retrieved from the PubChem database. In this study, we evaluated the physicochemical and medicinal properties of the compounds identified in *S. mannii* aqueous roots extract using ADMETLAB3.0, including the molecular weight, number of rings, formal charge, number of heteroatoms, number of atoms in the biggest ring, number of rotatable bonds, topological polar surface area, number of hydrogen bond donors/acceptors, n-octanol/water distribution coefficient (logP), logP at pH = 7.4, and the aqueous solubility. The drug-likeness of the compounds was evaluated using the Lipinski rule (a maximum of 5 hydrogen bond donors, a maximum of 10 hydrogen bond acceptors, a molecular weight of a maximum of 500 Daltons, and an octanol-water partition coefficient (logP) of less than 5). Compounds with more than one violation of the Lipinski rule were not considered eligible for network pharmacology-based drug discovery.

#### Identification of cervical cancer-associated *S. mannii* aqueous roots extract targets.

In this study, we used the GeneCards database, Pharos database, and DisGeNET database (accessed on 12^th^ July 2024) to retrieve targets of human cervical cancer. The following keywords were used: “cervical cancer”, “cervical carcinoma”, “cancer of the cervix”, “cancer of the cervix uterine”, and 11,180 unique targets of cervical cancer were identified. Similarly, targets of the compounds were obtained from the SuperPred database, Similarity Ensemble Approach database (SEA), and SwissTarget prediction database (with a probability >50%, accessed on 12^th^ July 2024). 742 unique targets of compounds were identified. The official symbols of targets (standard gene names) were normalized using the UniProt database (Retrieve/ID mapping, accessed on 12^th^ July 2024). Overlapping between the two datasets identified 493 targets of *S. mannii* aqueous roots extract, associated with cervical cancer.

#### Protein-protein interaction (PPI) network analysis.

This study aimed to explore the potential molecular mechanism by which *Solanecio mannii* aqueous roots extract exhibits its anti-cancer activity against cervical carcinoma. The new dataset, containing 493 cervical cancer-associated targets of *S. mannii* aqueous roots extract, was uploaded in the STRING database (accessed on 12^th^ July 2024). The organism was set as Homo sapiens, and the minimum required interaction score was set at high confidence (0.7). The protein-protein interaction network was visualized and analysed according to the degree of connectivity between nodes using Cytoscape (*V.* 3.8.2). CytoHubba plug-in was recruited to determine and visualize the top 30 degree-sorted genes (core targets).

#### Molecular docking.

The PubChem database was used to retrieve the SDF file of the ligands. Polar atoms were added to compounds using Discovery Studio Visualizer. Ligands were uploaded into PyRx software, and their energy was minimized with 200 steps. The Protein Data Bank (PDB) database was used to retrieve the 3D structures of target proteins in PDB format. Non-standard residues, heteroatoms, ions, water atoms, existing ligands, and non-target protein chains were removed from the structures using UCSF Chimera X. Structures were prepared for docking using the Dock Prep plug-in in UCSF Chimera X. Briefly, solvents and non-complexed ions were deleted, residues types were standardized, side chains were completed, and hydrogens and charges were added (PDB identifiers of the macromolecules and targeted chains are in Table 3).

**Table 3:** PDB identifiers and amino acids chains of target protein/macromolecules.

Protein/macromolecule	PDB ID	Chain
ESR1	1A52	A, B
NFKB1	3GUT	P65: A, C, E, G; P105: B, D, F, H
PIK3CA	7R9V	A
HIF1A	4ZPR	B
HSP90AA1	4BQG	A
HSP90AB1	5UC4	A, B, C, D
STAT3	6TLC	A, B
PPARG	8WFE	A, B

Molecular docking was conducted using Vina wizard built into PyRx with a maximized auto grid dimension. Poses with the lowest binding affinity and null RMSD values were selected. Intermolecular interactions were visualized using Discovery Studio Visualizer.

#### Molecular Dynamic (MD) Simulation.

Using the GROMACS simulation package, we processed the complex with the OPLS/AA force field and neutralized it to 0.15 molar in a triclinic box. The complex’s energy was minimized, and it was NVT/NPT-equilibrated for 100ps. The temperature was set at 300 K, while the pressure was set at 1 bar. The dynamic simulation was conducted for 100 ns (5000 frames). Visualization and post-MD simulation analyses was conducted using VMD and QT-GRACE software. The analysis included the Roots Mean Square Deviation (RMSD) of the protein, binding pocket, and the ligand; Roots Mean Square Fluctuation (RMSF); radius of gyration, total energy; secondary structure content (Define Secondary Structure of Proteins/ DSSP), Molecular Mechanics Poisson-Boltzmann Surface Area (MMPBSA); inter-hydrogen bonds, contact map, and Solvent Accessible Surface Area (SASA).

#### Gene annotation.

This study employed Gene Ontology (GO) to annotate the 180 cervical cancer-associated targets of *S. mannii* aqueous roots extract, according to Cellular Component (CC), Biological Process (BP), and Molecular Function (MF), using GORILLA webserver (accessed on 12^th^ July 2024). FDR-based top 5 annotations of gene ontology analysis were visualised using SRplot (accessed on 14^th^ July 2024).

#### Pathway enrichment analysis.

KEGG pathway enrichment was performed using the Database for Annotation, Visualization and Integrated Discovery (DAVID) database (accessed on 12^th^ July 2024). FDR-based top 20 pathways were visualized using SRplot (accessed on 14^th^ July 2024).

### (4′,6)-Diamidino-2-phenylindole (DAPI) staining

HeLa cells were seeded on membrane-treated slides in 24-well plates at a density of 2 × 10^5^ cells/well. After attachment, the cells were treated with 1 ml/well at a dosage of  ¼ IC_50_ for 18h and  ½ IC_50_ for 24 hours. After treatment, the slides were rinsed with PBS, fixed with 3.7% paraformaldehyde, and permeabilized with Triton-X. The samples were then stained with DAPI solution for 15 minutes at room temperature in the dark. Slides were washed twice with PBS and analysed using a fluorescence microscope.

### Monodansylcadaverine (MDC) staining

HeLa cells were seeded on membrane-treated slides in 24-well plates at a density of 2 × 10^5^ cells/well and allowed to attach overnight. Subsequently, they were treated with ¼ IC_50_ and ½ IC_50_ for 18, and 24 hours, respectively. The cells were rinsed with PBS, followed by the wash buffer, after treatment. The cells were incubated in the dark at room temperature for 30 minutes with MDC stain. The cells were rinsed with the wash buffer and then examined under a fluorescent microscope.

### Cell cycle analysis

In T25 cell culture flasks, 1 × 10^6^ cells were seeded and allowed to attach. The following day, the cells were subjected to treatment at ¼ IC_50_ and ½ IC_50_ for 18 hours and 24 hours, respectively. Accutase was used to collect the cells, which were subsequently washed twice with PBS, re-suspended in 70% (v/v) ethanol, and stored at -20°C overnight. The cells were washed with PBS and suspended in a staining buffer containing 50 μg/mL propidium iodide, 100 μg/mL RNase, and 0.1% Triton-X. Flow cytometry analysis was conducted after the cells were incubated with the staining buffer for 1 hour in the dark at room temperature.

### Analysis of the relative expression of target genes

In T75 flasks, at 80% confluence, HeLa cells were treated. After treatment, cells were washed twice with PBS, and RNA was extracted using the PureLink RNA Mini Kit, according to the manufacturer’s protocol. Briefly, cells were lysed using a lysis buffer supplemented with 1% 2-mercaptoethanol. The lysate was homogenized and supplemented with an equal volume of cold absolute ethanol. RNA was bound to spin cartridge membranes, washed, and eluted. Following the manufacturer’s recommendations, cDNA was reverse transcribed in a reaction volume of 20 μL using the SensiFAST^TM^ cDNA Synthesis Kit. The concentrations of the cDNA samples were measured using a Nanodrop, standardised to 900 ng/μL, and then diluted (1:9) with nuclease-free water. The SolisFAST® SolisGreen® quantitative polymerase chain reaction (qPCR) Master Mix Kit was used to perform quantitative real-time polymerase chain reaction (qRT-PCR) in accordance with the manufacturer’s protocol.

Table 4 displays the forward and reverse primer sequences of target genes and β-tubulin (housekeeping gene). Relative gene expression was quantified according to the 2^-ΔΔCt^ method.

**Table 4:** Forward and reverse primers’ sequences of target genes.

Gene	Forward primer (5′- > 3′)	Reverse primer (5′- > 3′)
ATG13	TGTGGAAACCTGTGATCCAT	CTTGTCCAGGTCCTTTCTGT
Beclin1	GGACAGTTTGGCACAATCAA	CCCAGAACAGTATAACGGCA
LC3	AGAAAGGATTTTGAGGAGGGG	TGAGACAGTGTCAGAACTACC
BAK	CAGGACACAGAGGAGGTTTT	TGCTAGGTTGCAGAGGTAAG
BAX	CTGAGCAGATCATGAAGACAG	TCCTCTGCAGCTCCATGTTA
FASL	AGCATCATCTTTGGAGAAGC	TGGACCTTGAGTTGGACTTG
APAF1	TGTAGTCCCTGTGGAGAGTT	CGTTCCACCACTTTACATTCT
chop c/EBP	GAACCAGGAAACGGAAACAG	GTTCATGCTTGGTGCAGATT
Noxa	GAAGTCGAGTGTGCTACTCA	CCTGAGCAGAAGAGTTTGGA
PUMA	CAGGGCAGGAAGTAACAATG	ACCCCATGCCAAATTTCATC
P16	GCCTTTTCACTGTGTTGGAG	ATTTGAGCTTTGGTTCTGCC
P21	TGCCGAAGTCAGTTCCTTGT	TCTCGGTGACAAAGTCGAAG
STAT3	GAGAGATTGACCAGCAGTAT	ATACCTGCTCTGAAGAAACT
PIK3CA	GACCCGATGCGGTTAG	GATGTATGGTTGTCCCAAAG
HIF1A	AGGATCACCCTCTTCGT	AAGAGAAGGAAAGGCAAGT
PPARG	CGAGGACACCGGAGA	CATTTCGTTAAAGGCTGACT
NFKB1	CCGCTTAGGAGGGAGA	CTCTCTGTTTAGGTTGCTCT
ESR1	TCTAACCTCGGGCTGTG	CTTGGATCTGATGCAGTAGG
HSP90AA1	TCCTGTGCGGTCACTT	CCAGAGTCTAATTTACTGGGA
HSP90AB1	TATCGGAAAGCAAGCCTAC	ATTGATGATGAGGGACATGA
RB1	GGATATACTCTACCCTGCGATTT	CTGTCAGCCTTAGAACCATGTT
E6	TTGCTTTTCGGGATTTATGC	CAGGACACAGTGGCTTTTGA
E7	GAACCGGACAGAGCCCATTA	AGAACAGATGGGGCACACAAT
β-tubulin	TTTCTTGCCCCATACATACC	CCTGTGGCTTCATTGTAGTA

### Statistical analysis

All experiments were performed in triplicates. Using GraphPad Prism (*v.*10), two-way ANOVA was utilised to evaluate the quantitative data between the groups; a p-value of less than 0.05 was considered statistically significant.

## Results

### High-throughput metabolomics profiling of the aqueous extract of *S. mannii* roots

**Fig 1:**
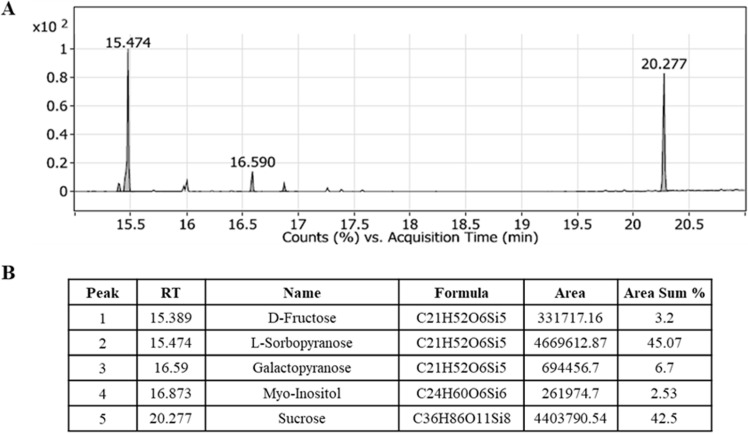
Metabolomics profiling of the aqueous extract of *Solanecio mannii* roots using GC-MS.

Our study provides a high-throughput metabolic fingerprint of the aqueous extract of *S. mannii* roots using both gas chromatography and liquid chromatography, each coupled to mass spectrometry. The study identified the volatile and thermally stable compounds (organic acids, fatty acids, sugars, and small phenolic compounds) by subjecting the extract to derivatization, which increased the phytocompounds volatility for more efficient vaporization and separation in the chromatographic columns. The GC-MS analysis revealed the presence of L-sorbopyranose, a ketose sugar isomer of L-sorbose, as the major component in the extract, accounting for 45% of the area sum. Monosaccharides, including D-fructose and galactopyranose, and disaccharides (sucrose, 42.5%) were also identified. Due to derivatization, the hydrogen atoms in the hydroxyl groups of Myo-inositol were replaced by TMS groups, enabling the effective detection of this sugar alcohol by GC-MS. Fig 1 shows the five peaks detected by GC-MS, along with their respective retention times and area sums.

**Fig 2:**
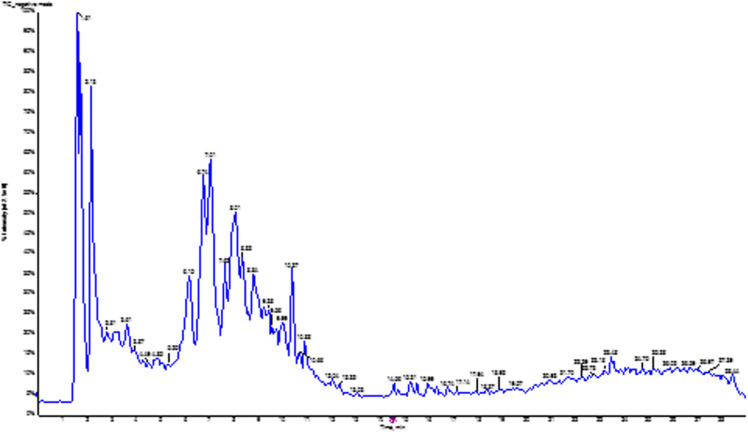
Total ion chromatogram (TIC) of the aqueous extract of *Solanecio mannii* roots in negative ionization mode. The number above each peak corresponds to the retention time.

**Fig 3:**
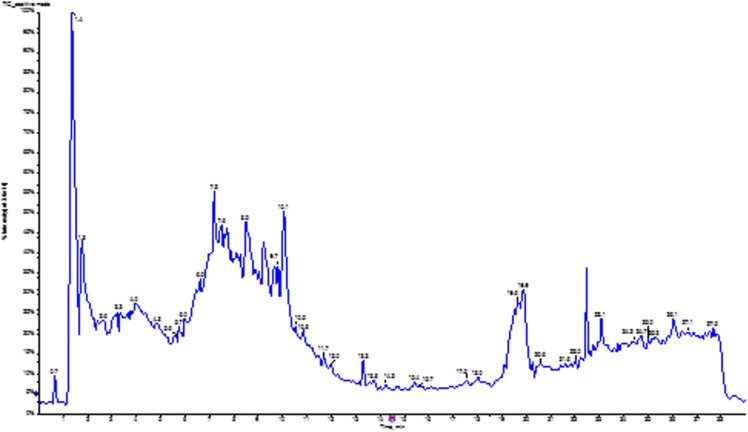
Total ion chromatogram (TIC) of the aqueous extract of *Solanecio mannii* roots in positive ionization mode. The number above each peak corresponds to the retention time.

In contrast, without the need for extensive derivatization, liquid chromatography-electrospray ionization-tandem mass spectrometry is effective for high-throughput profiling of non-volatile, large, and thermally labile compounds, including lipids, peptides, and secondary metabolites (alkaloids and flavonoids). The positive ionization mode enables the detection of alkaloids, peptides, and primary metabolites with basic functional groups that gain a proton, whereas the negative ionization mode is suitable for the detection of acidic or neutral compounds, such as phenolic acids, flavonoids, and fatty acids. Figs 2 and 3 show the total ion chromatograms of the aqueous extract of *Solanecio mannii* roots in negative and positive ionization modes, respectively.

A total of 41 compounds were identified using liquid chromatography-electrospray ionization-tandem mass spectrometry technique in negative and positive ionization modes (23 and 18 biomolecules, respectively).

This study revealed the presence of L-methionine sulfone as a major compound (molecule **13–15**), followed by caffeic acid (**9**), baicalein-7-O-glucuronide (**17–19**), glycyl-L-proline (**3–6**), citrate (**7**), peonidine-3-O-glucoside chloride (**10**), cis-aconitate (**12**), DL-glyceraldehyde 3-phosphate (**1** and **2**), N-formyl-L-methionine (**11**), L-beta-homotryptophan-HCl (**22**), NADP^+^ (**21**), thymidine-5’-triphosphate sodium salt (**20**), farnesol (mixture of isomers) (**23**), thymidine-5’-monophosphate (**8**), and inosine-5’-triphosphate trisodium salt (**16**). Table 5 shows the metabolomic profile of the aqueous extract of *S. mannii* roots, analysed by LC-ESI-MS/MS in the negative mode.

**Table 5:** LC-ESI-MS/MS-based metabolomics profile of *S. mannii* aqueous extract of the roots in the negative ion mode.

Index	RT (min)	m/z	Adduct	Metabolite name	Area	Reference
**1**	1.515317	168.96	[M-H]–	DL-Glyceraldehyde 3-phosphate	2.44E + 08	MSBNK-RIKEN-PR100709
**2**	1.515317	169.08	[M-H]–	DL-Glyceraldehyde 3-phosphate	2.72E + 08	
**3**	1.515317	171.12	[M-H]–	Glycyl-L-proline	4.40E + 08	MSBNK-RIKEN_ReSpect-PS100004 MoNA ID: PR100854
**4**	1.515317	171.24	[M-H]–	Glycyl-L-proline	5.02E + 08	
**5**	1.577283	170.88	[M-H]–	Glycyl-L-proline	3.21E + 08	
**6**	1.577283	171	[M-H]–	Glycyl-L-proline	3.93E + 08	
**7**	1.697783	190.92	[M-H]–	Citrate	1.07E + 09	MSBNK-NAIST-KNA00814
**8**	3.257567	321.08	[M-H]–	Thymidine-5’-monophosphate	9.60E + 07	MSBNK-RIKEN-PR100611
**9**	3.52675	179.04	[M-H]–	Caffeic acid	2.37E + 09	MSBNK-Keio_Univ-KO000512
**10**	6.65985	461.16	[M-H]–	Peonidine-3-O-glucoside chloride	5.52E + 08	Spectra ID 439217
**11**	6.7833	175.92	[M-H]–	N-Formyl-L-Methionine	4.28E + 08	MSBNK-Keio_Univ-KO000800
**12**	7.019834	172.92	[M-H]–	cis-Aconitate	5.21E + 08	MSBNK-RIKEN-PR100734
**13**	7.019834	179.88	[M-H]–	L-Methionine sulfone	1.25E + 09	MoNA ID: PT102820
**14**	7.019834	180	[M-H]–	L-Methionine sulfone	1.00E + 09	
**15**	7.019834	180.12	[M-H]–	L-Methionine sulfone	8.22E + 08	
**16**	7.15395	507.04	[M-H]–	Inosine-5’-triphosphate trisodium salt	8.53E + 07	MSBNK-RIKEN_ReSpect-PS025909
**17**	10.36207	444.96	[M-H]–	Baicalein-7-O-glucuronide	6.18E + 08	MSBNK-RIKEN_ReSpect-PS041507
**18**	10.36207	445.08	[M-H]–	Baicalein-7-O-glucuronide	7.30E + 08	
**19**	10.36207	445.2	[M-H]–	Baicalein-7-O-glucuronide	8.66E + 08	
**20**	10.36207	481.08	[M+Cl]–	Thymidine-5’-triphosphate sodium salt	1.06E + 08	Spectra ID: 1472212
**21**	10.67688	743.04	[M-H]–	NADP+	1.93E + 08	MSBNK-NAIST-KNA00210
**22**	10.87507	217.08	[M-H]–	L-beta-homotryptophan-HCl	1.94E + 08	MSBNK-RIKEN_ReSpect-PS061501
**23**	10.9487	221.28	[M-2H]2-	Farnesol (mixture of isomers)	1.06E + 08	MSBNK-RIKEN_ReSpect-PS088103

On the other hand, the characterisation of the extract’s phytocompounds with the positive mode identified guanine as the major compound (**29–31**), followed by L-proline (**24** and **25**), 2-(4-isobutylphenyl) propionic acid (**32** and **33**), nicotinamide (**26** and **27**), alpha-D-glucose-1,6-diphosphate (**39** and **40**), N-tigloylglycine (**37** and **38**), N,N-dimethylaniline (**28**), 1-decanoyl-2-hydroxy-sn-glycero-3-phosphocholine (**35**), cerulenin (**41**), trans-zeatin-9-glucoside (**36**), and (-)-riboflavin (**34**). Table 6 shows the metabolomics profile of the studied extract analysed in the positive mode. S1–S26 Figs in S1 File shows the fragmentation patterns of the identified compounds.

**Table 6:** LC-ESI-MS/MS-based metabolomics profile of *S. mannii* aqueous extract of the roots in the positive ion mode.

Index	RT (min)	m/z	Adduct	Metabolite name	Area	Reference
**24**	1.3467	116.04	[M+H]+	L-Proline	5.34E + 08	MSBNK-Antwerp_Univ-METOX_P100401_EF88
**25**	1.369583	116.16	[M+H]+	L-Proline	7.20E + 08	
**26**	1.7129	122.88	[M+H]+	Nicotinamide	2.99E + 08	MSBNK-BGC_Munich-RP022303
**27**	1.7129	123	[M+H]+	Nicotinamide	4.17E + 08	
**28**	1.84995	122.16	[M+H]+	N,N-dimethylaniline	3.88E + 08	MSBNK-Fac_Eng_Univ_Tokyo-JP007684
**29**	2.08325	151.92	[M+2H]2+	Guanine	2.03E + 09	Spectra ID: 438628
**30**	2.08325	152.04	[M+NH4]+	Guanine	2.28E + 09	
**31**	2.08325	152.16	[M+NH4]+	Guanine	1.92E + 09	
**32**	5.968884	207.12	[M+H]+	2-(4-isobutylphenyl) propionic acid	5.02E + 08	MSBNK-Fac_Eng_Univ_Tokyo-JP004112
**33**	6.038	207.24	[M+H]+	2-(4-isobutylphenyl) propionic acid	3.00E + 08	
**34**	8.919117	377.16	[M+H-H2O]+	(-)-Riboflavin	2.05E + 08	MSBNK-BGC_Munich-RP013102
**35**	8.919117	412.2	[M+NH4]+	1-Decanoyl-2-Hydroxy-sn-Glycero-3-Phosphocholine	3.54E + 08	MSBNK-RIKEN_ReSpect-PS081402
**36**	10.86075	382.2	[M+H]+	trans-Zeatin-9-glucoside	2.06E + 08	MSBNK-RIKEN-PR020114
**37**	13.29873	158.04	[M+2H]2+	N-Tigloylglycine	2.06E + 08	MSBNK-RIKEN_ReSpect-PT110020
**38**	13.29873	158.16	[M+2H]2+	N-Tigloylglycine	2.07E + 08	
**39**	26.03918	341.04	[M+H]+	alpha-D-Glucose-1,6-diphosphate	2.10E + 08	MSBNK-RIKEN-PR100628
**40**	26.03918	341.16	[M+H]+	alpha-D-Glucose-1,6-diphosphate	2.41E + 08	
**41**	28.33378	224.04	[M+H]+	Cerulenin	2.07E + 08	MSBNK-RIKEN-PR100440

The targeted metabolic profiling of the extract revealed the presence of rutin 609/299.9 (0.4 ug/g) and chlorogenic acid 353/191 (17217.8 ug/g).

### *S. mannii* aqueous roots extract reduced the viability of HeLa cells

**Fig 4:**
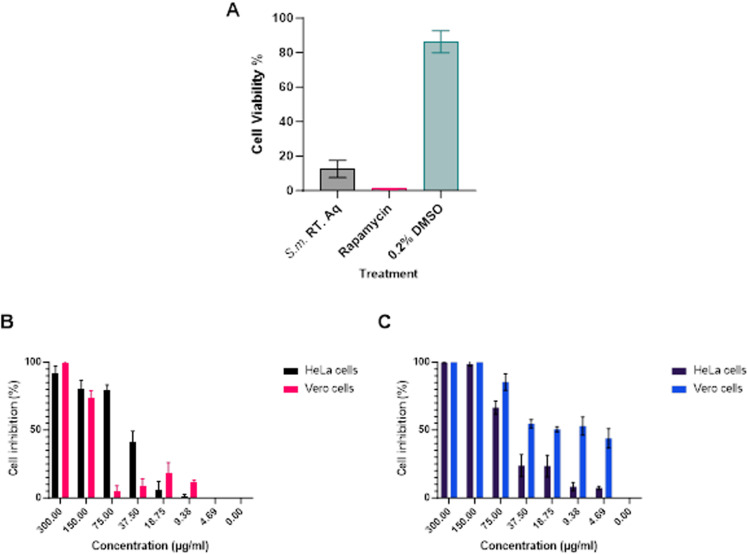
Cytotoxicity on HeLa cells upon treatment for 48h using CCK8 assay. **A.** Cell viability of HeLa cells after treatment at 200ug/ml. **B.** Dose-dependent cell inhibition upon treatment with *Solanecio mannii* aqueous roots extract. **C.** Dose-dependent cell inhibition upon treatment with rapamycin. S.m. RT. Aq: *Solanecio mannii* aqueous roots extract. Data is presented as mean ± SD.

Our study investigated the effect of *S. mannii* aqueous extract of the roots on the viability of human cervical cancer cell lines, HeLa cells, upon treatment, over a period of 48h, with 200 µg/ml of the extract. *S. mannii* aqueous roots extract induced a significant inhibition of cell growth, corresponding with a notable reduction in cell viability to 12.53 ± 4.983 μg/ml, compared to the viability of 86.27 ± 6.314 μg/ml noted in the negative control group (S1 Table in S1 File). These findings suggest that the aqueous extract of *S. mannii* roots possesses cytotoxic properties against human cervical cancer cell lines (HeLa cells). Fig 4A shows the cell viability recorded upon treatment of HeLa cells with 200 µg/ml for 48h. Furthermore, by applying a dose-dependent study, we evaluated the potency of *Solanecio mannii* aqueous roots extract to induce inhibition of HeLa and Vero cells, compared to the positive control, rapamycin (Fig 4B and 4C).

Our study showed that the plant extract exhibits a potent cytotoxic effect against HeLa cells, with an IC_50_ of 51.28 ± 1.91 μg/ml. Concurrently, this study employed the cytotoxicity assay to gauge the toxicity levels against non-cancerous cells, in which *Solanecio mannii* aqueous roots extract exhibited a selectivity index >2 with a CC_50_ of 121.53 ± 6.29 μg/ml. These findings highlight, for the first time, the anti-cancer potency of *Solanecio mannii* aqueous roots extract and its safe therapeutic window. On the other hand, our study revealed the non-selectivity of rapamycin with an IC_50_ of 58.41 ± 0.44 μg/ml and a CC_50_ of 20.09 ± 1.01 μg/ml. Indeed, our study reveals *S. mannii* extract as a safe, potent alternative for the management of cervical cancer, mitigating drug resistance mechanisms. The cytotoxicity analysis on other cell lines (SiHa, CaSki, and C33A) was not conducted in this study due to their unavailability.

### Computational-based investigation of the molecular mechanism of *Solanecio mannii* aqueous roots extract against cervical cancer

#### Drug-likeness and medicinal properties of the identified compounds.

Our study adhered to the Lipinski rule to identify the potential candidates that play a significant role in the efficacy of *S. mannii* multi-component extract. To achieve the desired therapeutic effect, the compounds should have physicochemical properties that enhance their absorption and permeation in the body, including 1) a maximum of 5 hydrogen bond donors (OH and NH groups), 2) a maximum of 10 hydrogen bond acceptors (N and O atoms), 3) a molecular weight of maximum 500 Daltons, and 4) a good solubility in aqueous and lipid milieus indicated by the octanol-water partition coefficient (logP) of less than 5. Table 7 presents a summary of the Lipinski Rule-based drug-likeness of metabolites, together with their PubChem IDs and their Simplified Molecular-Input Line-Entry System (SMILES) chemical identifiers. Our findings revealed 8 compounds unsuitable for oral drug development and have been excluded from our computational study (baicalein-7-O-glucuronide, peonidine-3-O-glucoside chloride, NADP^+^, inosine-5′-triphosphate trisodium salt, alpha-D-glucose-1,6-diphosphate, trans-zeatin-9-glucoside, sucrose, and rutin).

**Table 7:** Drug-likeness of the identified compounds.

Metabolite name	PubChem ID	SMILES	Lipinski Rule Eligibility
L-Methionine sulfone	445282	CS(=O)(=O)CCC(C(=O)O)N	Yes
Caffeic acid	689043	C1 = CC(=C(C = C1C=CC(=O)O)O)O	Yes
Baicalein-7-O-glucuronide	1549028	C1 = CC = C(C = C1)C2 = CC(=O)C3 = C(C(=C(C = C3O2)OC4C(C(C(C(O4)C(=O)O)O)O)O)O)O	No
Glycyl-L-proline	3013625	C1CC(N(C1)C(=O)CN)C(=O)O	Yes
Citrate	31348	C(C(=O)[O-])C(CC(=O)[O-])(C(=O)[O-])O	Yes
Peonidine-3-O-glucoside chloride	14311151	COC1 = C(C = CC(=C1)C2=[O+]C3 = CC(=CC(=C3C=C2OC4C(C(C(C(O4)CO)O)O)O)O)O)O	No
cis-Aconitate	643757	C(C(=CC(=O)O)C(=O)O)C(=O)O	Yes
DL-Glyceraldehyde 3-phosphate	729	C(C(C = O)O)OP(=O)(O)O	Yes
N-Formyl-L-Methionine	439750	CSCCC(C(=O)O)NC = O	Yes
L-beta-homotryptophan-HCl	2761549	C1 = CC = C2C(=C1)C(=CN2)CC(CC(=O)O)N.Cl	Yes
NADP^+^	5886	C1 = CC(=C[N+](=C1)C2C(C(C(O2)COP(=O)(O)OP(=O)(O)OCC3C(C(C(O3)N4C=NC5 = C(N = CN = C54)N)OP(=O)(O)O)O)O)O)C(=O)N	No
Thymidine-5’-triphosphate sodium salt	5311491	CC1 = CN(C(=O)NC1 = O)C2CC(C(O2)COP(=O)([O-])OP(=O)([O-])OP(=O)(O)[O-])O.[Na+].[Na+].[Na+]	Yes
Farnesol (mixture of isomers)	445070	CC(=CCCC(=CCCC(=CCO)C)C)C	Yes
Thymidine-5’-monophosphate	9700	CC1 = CN(C(=O)NC1 = O)C2CC(C(O2)COP(=O)(O)O)O	Yes
Inosine-5’-triphosphate trisodium salt	135742691	C1 = NC2 = C(C(=O)N1)N = CN2C3C(C(C(O3)COP(=O)([O-])OP(=O)([O-])OP(=O)(O)[O-])O)O.[Na+].[Na+].[Na+]	No
Guanine	135398634	C1 = NC2 = C(N1)C(=O)NC(=N2)N	Yes
L-Proline	145742	C1CC(NC1)C(=O)O	Yes
2-(4-isobutylphenyl) propionic acid	3672	CC(C)CC1 = CC = C(C = C1)C(C)C(=O)O	Yes
Nicotinamide	936	C1 = CC(=CN = C1)C(=O)N	Yes
alpha-D-Glucose-1,6-diphosphate	82400	C(C1C(C(C(C(O1)OP(=O)(O)O)O)O)O)OP(=O)(O)O	No
N-Tigloylglycine	6441567	CC = C(C)C(=O)NCC(=O)O	Yes
N, N-dimethylaniline	949	CN(C)C1 = CC = CC = C1	Yes
1-Decanoyl-2-Hydroxy-sn-Glycero-3-Phosphocholine	22851442	CCCCCCCCCC(=O)OCC(COP(=O)([O-])OCC[N+](C)(C)C)O	Yes
Cerulenin	5282054	CC = CCC = CCCC(=O)C1C(O1)C(=O)N	Yes
trans-Zeatin-9-glucoside	9842892	CC(=CCNC1 = C2C(=NC = N1)N(C = N2)C3C(C(C(C(O3)CO)O)O)O)CO	No
(-)-Riboflavin	493570	CC1 = CC2 = C(C = C1C)N(C3 = NC(=O)NC(=O)C3 = N2)CC(C(C(CO)O)O)O	Yes
D-Fructose	2723872	C1C(C(C(C(O1)(CO)O)O)O)O	Yes
L-Sorbopyranose	439192	C1C(C(C(C(O1)(CO)O)O)O)O	Yes
Galactopyranose	6036	C(C1C(C(C(C(O1)O)O)O)O)O	Yes
Myo-Inositol	892	C1(C(C(C(C(C1O)O)O)O)O)O	Yes
Sucrose	5988	C(C1C(C(C(C(O1)OC2(C(C(C(O2)CO)O)O)CO)O)O)O)O	No
Rutin	5280805	CC1C(C(C(C(O1)OCC2C(C(C(C(O2)OC3 = C(OC4 = CC(=CC(=C4C3=O)O)O)C5 = CC(=C(C = C5)O)O)O)O)O)O)O)O	No
Chlorogenic acid	1794427	C1C(C(C(CC1(C(=O)O)O)OC(=O)C = CC2 = CC(=C(C = C2)O)O)O)O	Yes

#### Protein-Protein interaction network.

**Fig 5:**
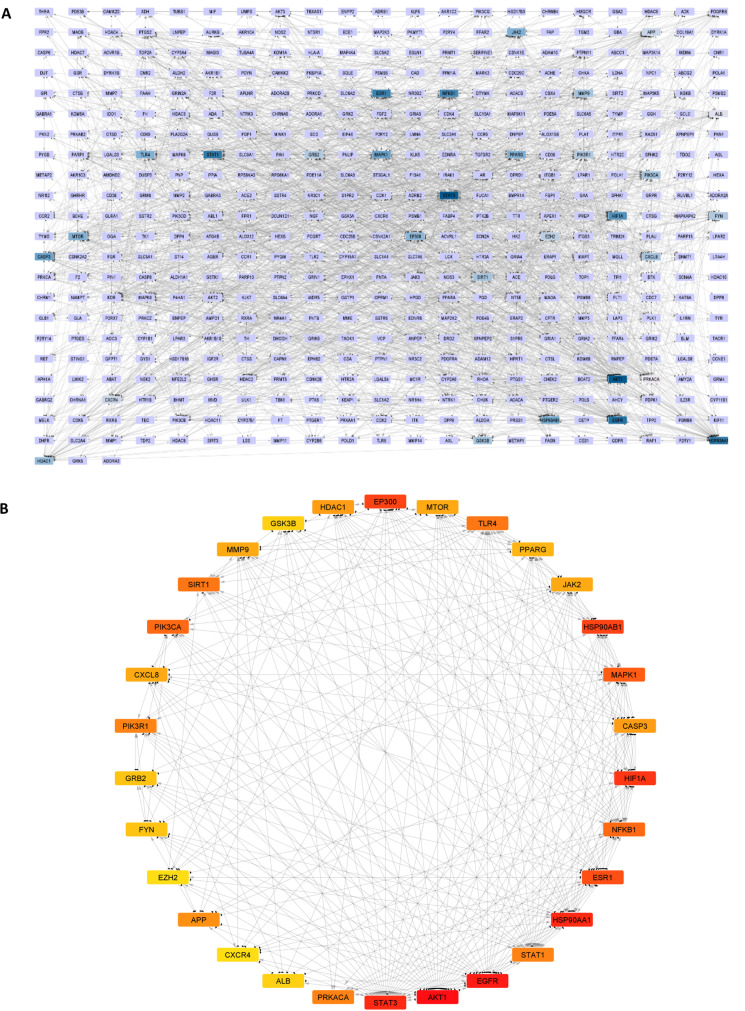
The protein-protein interaction (PPI) network diagram of *Solanecio mannii* aqueous roots extract targets associated with cervical cancer. **A.** PPI network of 465 cervical cancer-associated targets of the studied extract. **B.** PPI network of top 30 targets based on degree of connectivity. A node represents one target, while the edge represents the interaction. The colour degree intensifies according to the number of edges connected to a node in the network.

Our study revealed that the molecular mechanism of *Solanecio mannii* involved a protein-protein interaction network (PPI) of 465 nodes and 2230 edges (Fig 5A). According to the degree of connectivity (DC), the core targets were AKT1 (DC = 81),EGFR (DC = 70), HSP90AA1 (DC = 67),STAT3 (DC = 67), HIF1A (DC = 48), HSP90AB1 (DC = 47), EP300 (DC = 47), ESR1 (DC = 44), MAPK1 (DC = 43), PIK3CA (DC = 42), NFKB1 (DC = 42), TLR4 (DC = 41), SIRT1 (DC = 41), PIK3R1 (DC = 40), PRKACA (DC = 40), STAT1 (DC = 40), APP (DC = 38), CASP3 (DC = 37), HDAC1 (DC = 37), MMP9 (DC = 36), JAK2 (DC = 36),CXCL8 (DC = 36), MTOR (DC = 36), PPARG (DC = 35),FYN (DC = 34), GRB2 (DC = 34), ALB (DC = 31), GSK3B (DC = 31), CXCR4 (DC = 29), and EZH2 (DC = 29). Fig 4B shows the PPI network between the 30 core targets (Fig 5B).

#### Gene ontology annotation.

**Fig 6:**
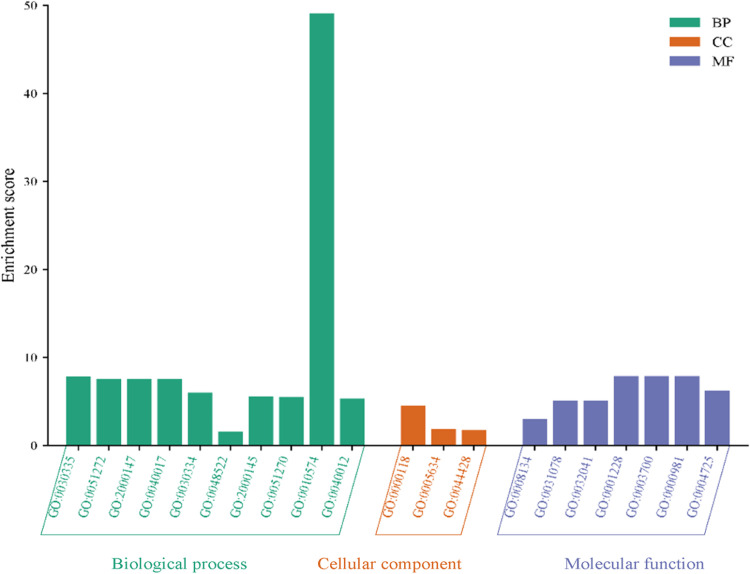
Gene ontology annotation of cervical cancer-associated targets of *S. mannii* aqueous roots extract.

The gene ontology (GO) study revealed that *S. mannii* aqueous roots extract was targeting proteins involved in 48 biological processes (BP). The studied extract was mainly targeting cell migration pathways, including the regulation and positive regulation of cell migration (GO:0030334, GO:0030335), regulation and positive regulation of cell motility (GO:2000145, GO:2000147), regulation and positive regulation of cellular component movement (GO:0051270, GO:0051272), positive regulation of locomotion (GO:0040017), and mononuclear cell migration and its regulation pathway (GO:0071674, GO:0071675). Additionally, the extract showed a potential to target pathways related to angiogenesis, including the regulation and positive regulation of vascular endothelial growth factor production (GO:0010574, GO:0010575), regulation and the positive regulation of angiogenesis (GO:0045765, GO:0045766), and the regulation and the positive regulation of vasculature development (GO:1901342, GO:1904018). *S. mannii* aqueous roots extract exerts its therapeutic activity against cervical cancer by targeting signalling pathways implicated in transcription from RNA polymerase II promoter in response to oxidative stress, stress, and hypoxia (GO:0043619, GO:0036003, GO:0061419), regulation of metabolic (GO:0019222, GO:0031323, GO:0060255, GO:0080090) and cellular (GO:0048522) processes, and peptidyl-tyrosine dephosphorylation (GO:0035335). In addition, the enrichment analysis revealed that the most significant cellular components (CC) of targets of *S. mannii* aqueous roots extract include histone deacetylase complex (GO:0000118), nucleus (GO:0005634), and nuclear part (GO:0044428). The molecular functions targeted by *S. mannii* aqueous roots extract included: transcription factor binding (GO:0008134), histone deacetylase activity (H3-K14 specific) (GO:0031078), NAD-dependent histone deacetylase activity (H3-K14 specific) (GO:0032041), RNA polymerase II-specific- DNA-binding transcription activator activity (GO:0001228), DNA-binding transcription factor activity (GO:0003700), RNA polymerase II-specific- DNA-binding transcription factor activity (GO:0000981), and protein tyrosine phosphatase activity (GO:0004725). Fig 6 shows the FDR-based top 10 GO terms and their enrichment scores.

#### Pathways enrichment.

**Fig 7:**
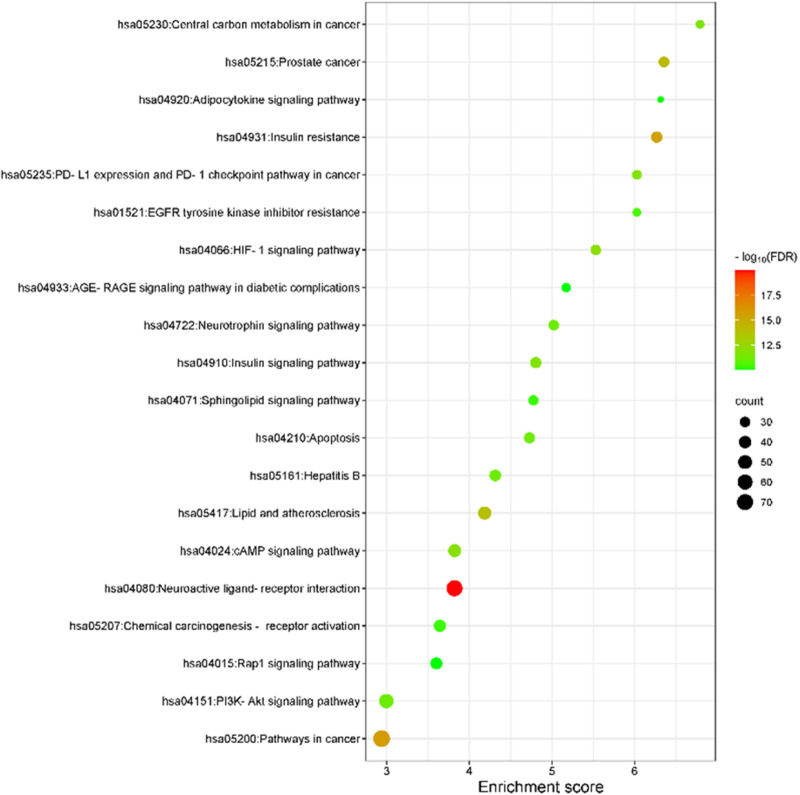
KEGG pathway enrichment of cervical cancer-associated targets of *S. mannii* aqueous roots extract.

The KEGG enrichment analysis in this study revealed the main signalling pathways, by which *S. mannii* aqueous roots extract exhibits its therapeutic activity against cervical cancer. The FDR-based top 20 KEGG pathways are shown in Fig 7. The genes, enrichment scores, and other information related to the KEGG enrichment analysis are shown in S2 Table in S1 File.

This study identified 178 KEGG pathways associated with the therapeutic activity of *S. mannii* aqueous roots extract, including pathways in cancer (hsa05200), central carbon metabolism in cancer (hsa05230), PD-L1 expression and PD-1 checkpoint pathway in cancer (hsa05235), viral carcinogenesis (hsa05203), proteoglycans in cancer (hsa05205), choline metabolism in cancer (hsa05231), microRNAs in cancer (hsa05206), transcriptional misregulation in cancer (hsa05202), chemical carcinogenesis (hsa05207 and hsa05208), apoptosis (hsa04210), and autophagy (hsa04140). In addition, the enrichment study indicated that *S. mannii* aqueous roots extract exerts its anticancer activity against cervical cancer by targeting several signalling pathways associated with cell survival, growth, proliferation, regulation of cell cycle, apoptosis, and migration, including HIF-1 (hsa04066), mTOR (hsa04150), PI3K-Akt (hsa04151), Rap1 (hsa04015), MAPK (hsa04010), ErbB (hsa04012), Ras (hsa04014), cAMP (hsa04024), AMPK (hsa04152), FoxO (hsa04068), chemokine (hsa04062), JAK-STAT (hsa04630), NF-kappa B (hsa04064) and P53 (hsa04115) signalling pathways. Additionally, the molecular mechanism of the studied extract included targeting metabolic pathways (hsa01100), nucleotide metabolism (hsa01232), and lysosomes (hsa04142). Moreover, this study indicated that the studied extract modulates the migration and metastasis potential of cervical cancer cells by targeting focal adhesion (hsa04510), Gap and adherent junctions (hsa04540 and hsa04520), VEGF and cGMP-PKG signalling pathway (hsa04370 and hsa04022), and the regulation of actin cytoskeleton (hsa04810). Furthermore, the extract is implicated in modulating the immune response by targeting IL-17 (hsa04657), T cell receptor (hsa04660), NOD-like receptor (hsa04621), Toll-like receptor (hsa04620), C-type lectin receptor (hsa04625), RIG-I-like receptor (hsa04622), and TNF (hsa04668) signalling pathways, Th17 and Th1/Th2 cell differentiation (hsa04659, hsa04658), and Natural killer cell-mediated cytotoxicity (hsa04650).

Notably, the studied extract targeted molecular mechanisms and signalling pathways related to drug resistance of EGFR tyrosine kinase inhibitors (hsa01521), platinum drugs (hsa01524), and antifolate drugs (hsa01523). Other pathways frequently related to drug resistance in cancer treatment are targeted by *S. mannii* aqueous roots extract, including proteoglycans in cancer (hsa05205), microRNAs in cancer (hsa05206), and chemical carcinogenesis - reactive oxygen species (hsa05208). *S. mannii* aqueous roots extract targets pathways related to oncogenic and non-oncogenic viral infections, including viral carcinogenesis (hsa05203), human papillomavirus (hsa05165), Kaposi sarcoma-associated herpesvirus (hsa05167), human T-cell leukaemia virus 1 (hsa05166), human immunodeficiency virus 1 (hsa05170), Epstein-Barr virus (hsa05169), and Hepatitis B and C (hsa05161 and hsa05160). Intriguingly, the pathway enrichment revealed the therapeutic activities of *S. mannii* aqueous roots extract against other metabolic, endocrine, infectious, neurodegenerative, and neurological diseases.

**Fig 8:**
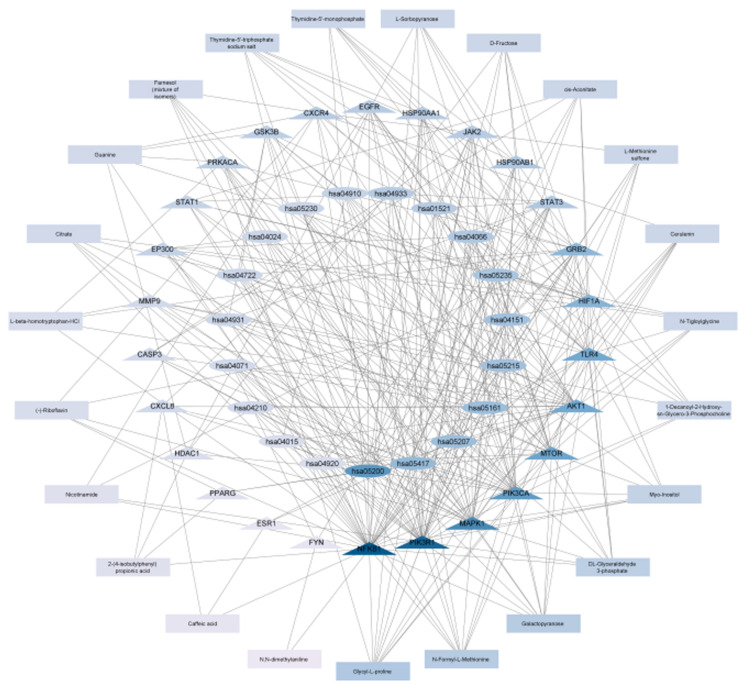
“Multi-component, multi-target, multi-pathway” molecular mechanism of *S. mannii* aqueous roots extract against cervical cancer. Rectangles represent compounds identified in *S. mannii* aqueous roots extract. Triangles represent the core targets. Ellipses represent the most enriched KEGG pathways. Intensity of the colour depends on the degree of connectivity.

Fig 8 shows *S. mannii* aqueous roots extract phytocompounds’ core targets-pathway interaction network, involving 23 compounds (chlorogenic acid and L-proline are not presented), core targets (SIRT, APP, ALB, and EZH are not presented), and top 20 FDR-enriched pathways (hsa04080 is not presented).

Our findings indicated glycyl-L-proline, galactopyranose, N-formyl-L-methionine, DL-glyceraldehyde 3-phosphate, and Myo-inositol as the core pharmacological phytocompounds of *S. mannii* aqueous roots extract by connecting to 8 or more core targets. Additionally, “pathways in cancer” (hsa05200) was the most enriched pathway, with a degree of connectivity (DC) = 24 core targets. All other pathways were enriched with a degree of connectivity >5 core targets. AKT1, PIK3R1, PIK3CA, MAPK1, NFKB1, and mTOR were implicated in 10 or more KEGG pathways. In addition, our study revealed that NFKB1 was targeted by all the compounds of the extract, whereas ESR1 was targeted only by caffeic acid. Our findings confirmed the theory that the compounds regulated multiple pathways through different targets, and different compounds can target the same targets and pathways. Therefore, *S. mannii* aqueous roots extract exhibits a multi-compound, multi-target, and multi-pathway molecular mechanism against cervical cancer.

#### Effect of *S. mannii* aqueous roots extract on the expression of core targets.

**Fig 9:**
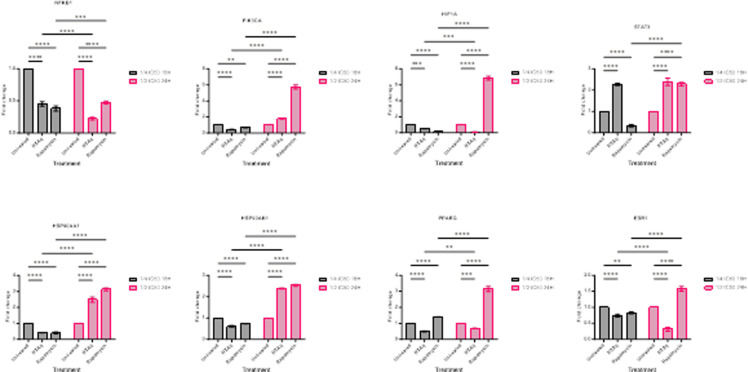
Effect of *S. mannii* aqueous roots extract on the expression of core targets.

To validate the efficacy of *S. mannii* aqueous roots extract against cervical cancer, qRT-PCR was used to assess the expression of some of the core targets identified in the network pharmacology analysis (NFKB1, PIK3CA, HIF1A, STAT3, HSP90AA1, HSP90AB1, PPARG, and ESR1), following treatment of HeLa cells with *S. mannii* aqueous roots extract in a time- and dose-dependent manner (S3 Table in S1 File). The extract induced downregulation of NFKB1, HIF1A, PPARG, and ESR1 in a dose- and time-dependent manner. A down-regulation of PIK3CA, HSP90AA1, and HSP90AB1 was observed when treated at a lower dosage (¼ IC_50_ for 18h), but significantly increased when treated with ½ IC_50_ for 24h, compared to the untreated group. The expression of STAT3 was found to be significantly increased at both dosages, compared to the untreated group (Fig 9).

#### Molecular docking.

**Fig 10:**
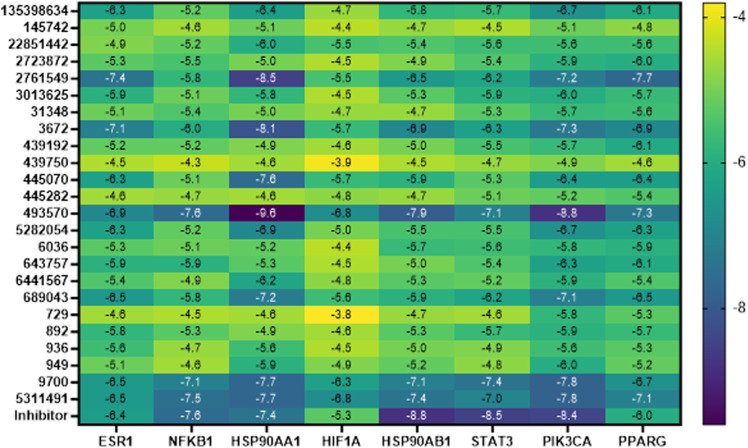
Binding affinity upon interaction between compounds and the studied core targets. The binding affinity represents in molecular docking score represented in kcal/mol.

According to the molecular docking study, our findings indicated strong intracomplex binding affinities as indicated by low docking scores, suggesting the potential of spontaneous binding. Fig 10 shows the binding affinities between core compounds and selected core targets, compared to their FDA-approved inhibitors. The lower the docking score, the stronger the interaction.

S27–S34 Figs in S1 File show the optimal binding patterns between the compounds and the studied core targets. S35 Fig in S1 File shows the interaction patterns between the studied targets and their FDA-approved inhibitors.

This study identified NFKB1 as the most frequently targeted protein with a strong binding affinity with (-)-riboflavin. In addition, the molecular docking study complemented the network pharmacology by revealing the interaction potency between the core targets and drug-like identified compounds, with the strongest binding affinity observed between HSP90AA1 interacted with (-)-riboflavin.

#### Dynamic simulation.

To further investigate the interaction of (-)-riboflavin with HSP90AA1 and evaluate their binding stability, a 100 ns molecular dynamics simulation was performed. This study employed the protein RMSD, binding pocket RMSD, ligand RMSD, RMSF, radius of gyration, total energy, and the secondary structure content (Define Secondary Structure of Proteins/ DSSP). The MMPBSA analysis was conducted to assess the binding free energy and complex stability.

**Fig 11:**
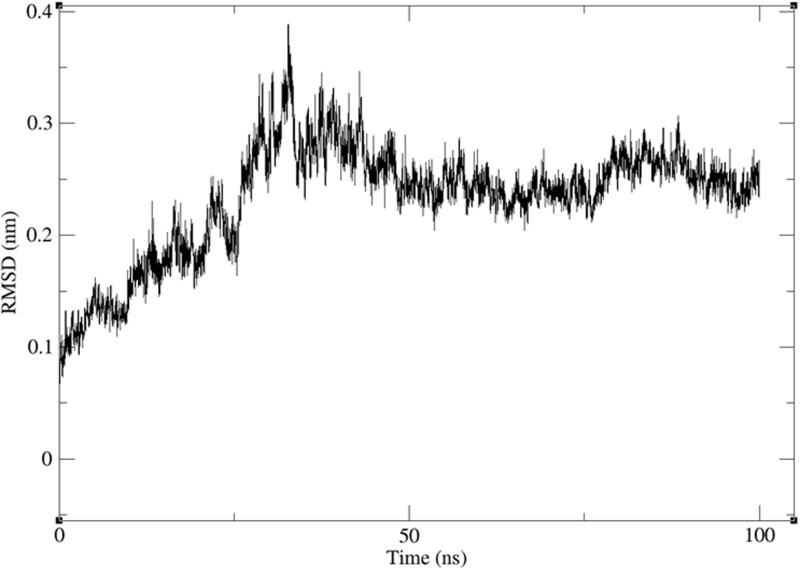
100ns-based RMSD of HSP90AA1 upon binding with (-)-riboflavin.

The interaction between HSP90AA1 and (-)-riboflavin induced conformational stability in the target protein with a RMSD of 2.352 ± 0.516 A° (0.2352 ± 0.0516 nm) with RMSD values ranging from 0.4 to 3.944 A° (Fig 11). The RMSD of the residues 22–186 involved in the intermolecular interactions showed an RMSD of 2.336 ± 0.563 A° with 0.387 and 4.156 A° as extreme RMSD of the residue’s backbone, whereas the ligand showed an RMSD of 4.271 ± 0.605 A°.

**Fig 12:**
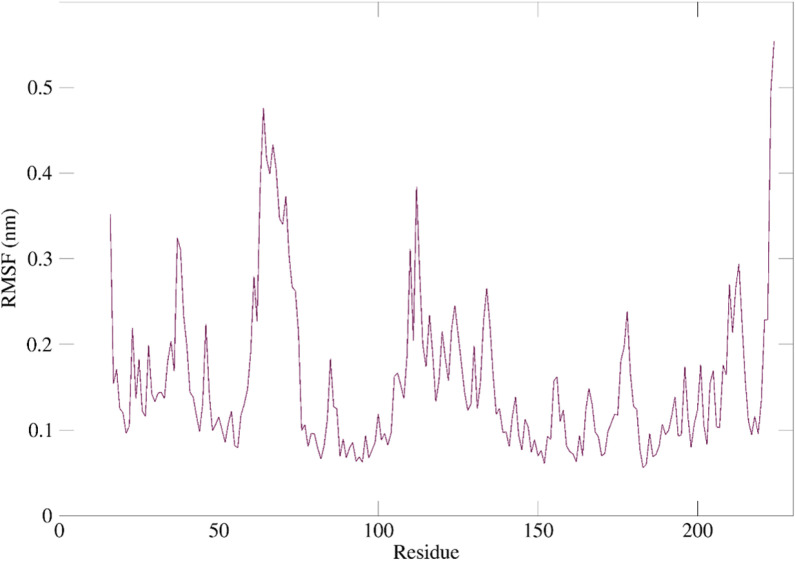
100ns-based RMSF of HSP90AA1 upon binding with (-)-riboflavin.

The study investigated the flexibility of each residue of HSP90AA1. The findings showed the residues 17–24, 26–37, 42–45, 48–61, 76–86, 91–109, 118–126, 128–137, 141–149, 153–159, 170–181, 183–200, 202–206, 210–213, 215–219, 220–222 have lower RMSF values (RMSF < 0.3 A°), which indicate more rigid/stable residues, while RMSF > 0.3 A°, suggesting regions with greater flexibility, were recorded in the residues 63–67, 69–72, 110–113, and 223–224 (Fig 12).

**Fig 13:**
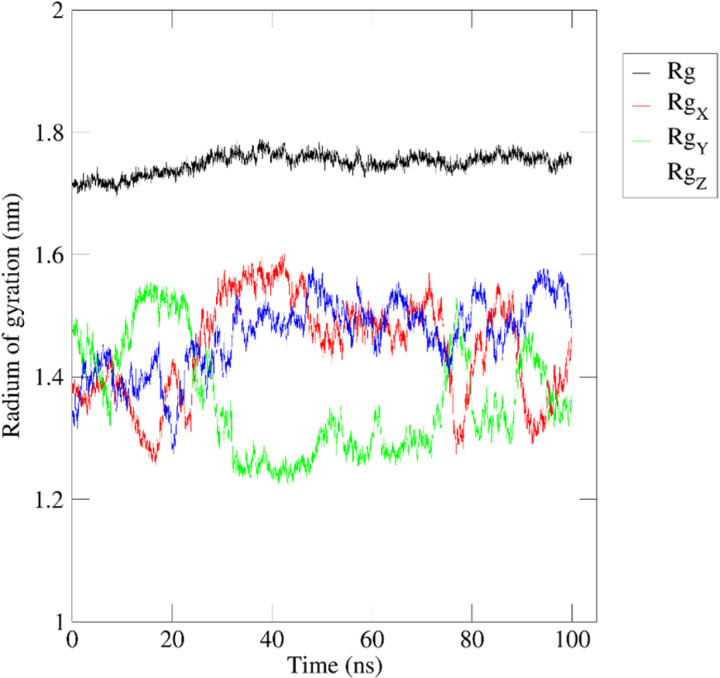
100ns-based radius of gyration of HSP90AA1 upon binding with (-)-riboflavin.

The average radius of gyration over the 100ns time frames was 1.749 ± 0.017 A° with an initial and final radius of gyration of 1.705 A° and 1.76594 A°, respectively, indicating a stable structure with minor expansion and fluctuation of the compactness (Fig 13).

Throughout the simulation timeframe, the average total energy was -302,392.378 ± 881.538 kJ/mol, indicating a stably bound and properly equilibrate system, with favourable interactions maintaining structural integrity and no major conformational changes (unfolding or structural rearrangements) upon binding of (-)-riboflavin to HSP90AA1. Additionally, the average number of intramolecular hydrogen bonds and pairs within 0.35 nm were 159.74 ± 7.117 and 888.868 ± 14.657, respectively, while the intermolecular hydrogen bonds averaged 1.135 ± 0.823 and 4.106 ± 1.804, respectively. Furthermore, the analysis of the secondary structure of HSP90AA1 throughout the simulation indicated a predominance of structured elements with 0.60 Secondary Structure Proportion (SS pr.) (including 0.25 SS pr. α-helix, 0.23 SS pr. β-sheet, and 0.12 SS pr. turn), 0.11 SS pr. bend, SS pr. 0.20 coil. These findings suggest a stable and well-organized HSP90AA1 secondary structure upon binding with (-)-riboflavin.

Table 8 provides detailed binding energy components that contributed to the stability of the HSP90AA1- (-)-riboflavin complex.

**Table 8:** MMPBSA analysis results.

Energy Component	Complex	Receptor	Ligand	Δ (Complex - Receptor - Ligand)
**BOND**	620.30 ± 18.36	605.53 ± 18.07	14.78 ± 2.94	0.00 ± 2.66
**ANGLE**	1676.10 ± 31.11	1572.27 ± 30.09	103.83 ± 4.06	-0.00 ± 3.04
**DIHED**	-4047.32 ± 36.85	-4067.74 ± 36.30	20.42 ± 2.25	0.00 ± 1.69
**VDWAALS**	-1597.64 ± 25.56	-1552.97 ± 24.75	-2.91 ± 1.02	-41.76 ± 0.21
**EEL**	-14671.44 ± 115.19	-14641.69 ± 114.97	-22.71 ± 1.61	-7.04 ± 1.39
**1-4 VDW**	1071.40 ± 14.32	1032.47 ± 13.77	38.93 ± 3.08	0.00 ± 2.53
**1-4 EEL**	5780.06 ± 22.06	5765.32 ± 22.09	14.74 ± 0.63	0.00 ± 0.60
**EPB**	-2842.85 ± 90.87	-2859.24 ± 90.72	-8.81 ± 0.60	25.20 ± 0.45
**ENPOLAR**	55.48 ± 1.05	56.48 ± 1.02	2.77 ± 0.03	-3.77 ± 0.00
**EDISPER**	0.00 ± 0.00	0.00 ± 0.00	0.00 ± 0.00	0.00 ± 0.00
**GGAS**	-11168.53 ± 131.44	-11286.81 ± 130.61	167.08 ± 6.61	-48.80 ± 1.40
**GSOLV**	-2787.37 ± 90.88	-2802.75 ± 90.72	-6.03 ± 0.60	21.42 ± 0.45
**TOTAL**	-13955.90 ± 159.80	-14089.56 ± 159.02	161.04 ± 6.63	-27.38 ± 1.47

**BOND:** bond Energy. **ANGLE**: angle energy. **DIHED**: dihedral energy. **VDWAALS**: Van der Waals energy. **EEL**: electrostatic energy. **1–4 VDW**: 1–4 Van der Waals energy. **1–4 EEL**: 1–4 electrostatic energy. **EPB**: electrostatic potential energy (Poisson-Boltzmann). **ENPOLAR**: non-polar contribution to solvation energy. **EDISPER**: Disperse Contribution to Solvation Energy. **GGAS**: gas-phase free energy. **GSOLV**: solvation free energy. **TOTAL**: total free energy.

**Fig 14:**
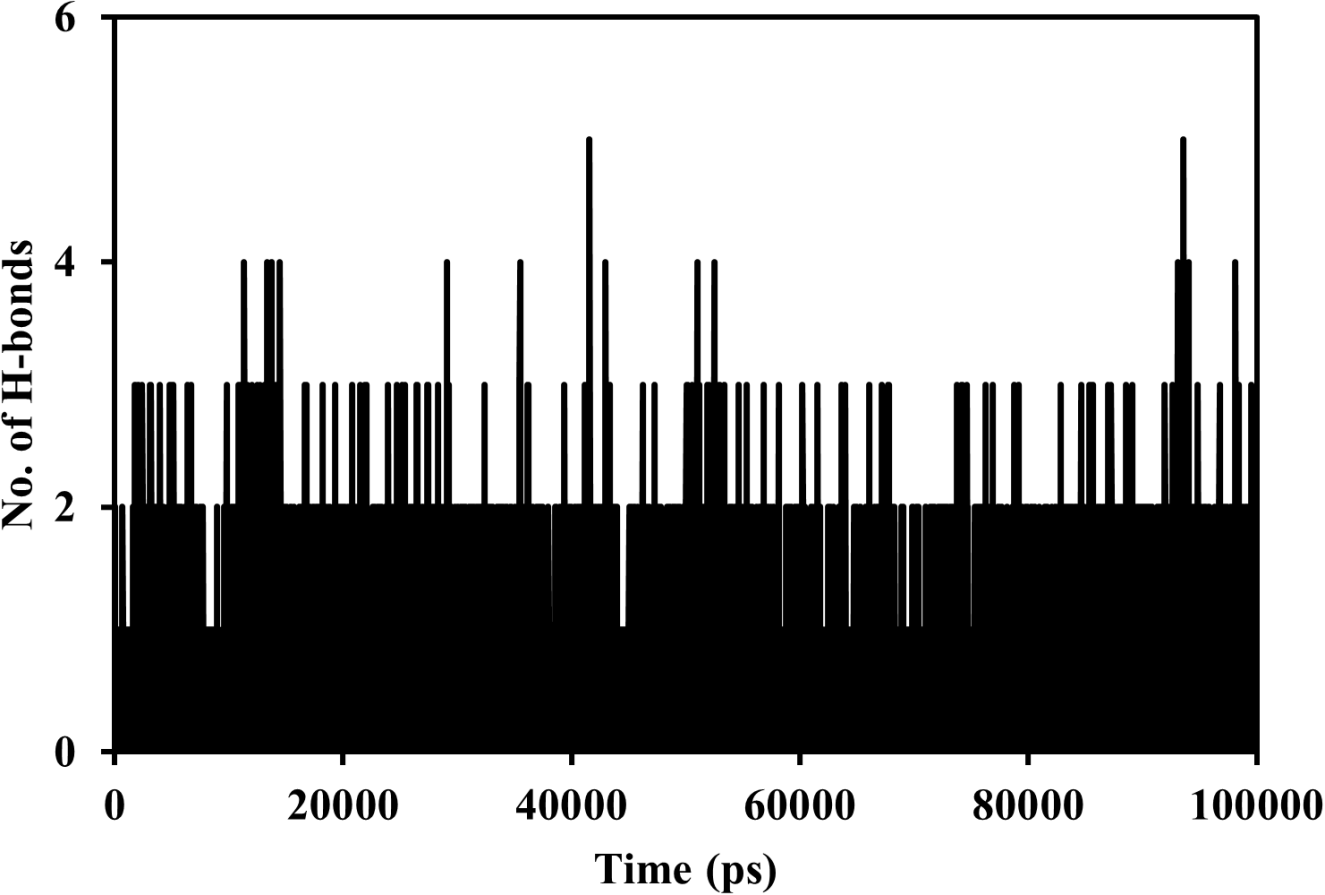
Inter hydrogen bonds between HSP90AA1 and (-)-riboflavin.

The intermolecular hydrogen bonds between protein and the ligand involved three H-bonds initially, increasing to 4-5 H-bonds; however, on average, only ~2-3 H-bonds were maintained, which stabilised the complex throughout the MD run (Fig 14).

The study that showed Phe138 was the only residue that maintained an occupancy of 2.94% H-bond (Main-Main) and 28.81% H-bond (Side-Side) with the ligand. Residue Asn51 has maintained 24.85% H-bond (Side-Side) occupancy with the ligand. Table 9 shows the hydrogen bond occupancy pattern between HSP90AA1 and (-)-riboflavin.

**Table 9:** Hydrogen bond occupancy analysis.

	Donor	Acceptor	Occupancy
Main (Donor) -Main (Acceptor) H-bond	VAL136	(-)-riboflavin	0.30%
	GLY137	(-)-riboflavin	0.76%
	PHE138	(-)-riboflavin	2.94%
Main (Donor) -side (acceptor) H-bond	PHE138	(-)-riboflavin	0.02%
	GLY97	(-)-riboflavin	0.18%
Side (Donor) -side (acceptor) H-bond	(-)-riboflavin	PHE138	28.81%
	(-)-riboflavin	TYR139	1.24%
	(-)-riboflavin	ASP93	0.18%
	LEU107	(-)-riboflavin	0.14%
	ASN51	(-)-riboflavin	24.85%
	(-)-riboflavin	ASN51	0.52%
	(-)-riboflavin	ILE96	1.06%
	LEU103	(-)-riboflavin	0.02%
	TYR139	(-)-riboflavin	0.02%
	(-)-riboflavin	ASP54	0.08%
	VAL150	(-)-riboflavin	0.06%
	ASN106	(-)-riboflavin	3.92%
	(-)-riboflavin	ALA55	0.08%
	ILE110	(-)-riboflavin	0.02%
	MET98	(-)-riboflavin	0.18%
	(-)-riboflavin	ILE110	0.02%
	LYS58	(-)-riboflavin	0.34%
	(-)-riboflavin	MET98	0.10%
	(-)-riboflavin	ASN106	1.40%
	(-)-riboflavin	ASP102	0.16%
	ALA55	(-)-riboflavin	0.02%
Side (Donor) - Main (acceptor) H-bond	ALA111	(-)-riboflavin	0.02%
	LEU107	(-)-riboflavin	0.06%
	TYR139	(-)-riboflavin	0.08%
	(-)-riboflavin	GLY135	1.62%
	(-)-riboflavin	GLY97	7.10%
	VAL136	(-)-riboflavin	0.02%
	ILE110	(-)-riboflavin	0.02%
	ASN51	(-)-riboflavin	0.72%
	PHE138	(-)-riboflavin	0.20%
	(-)-riboflavin	VAL136	0.02%

**Fig 15:**
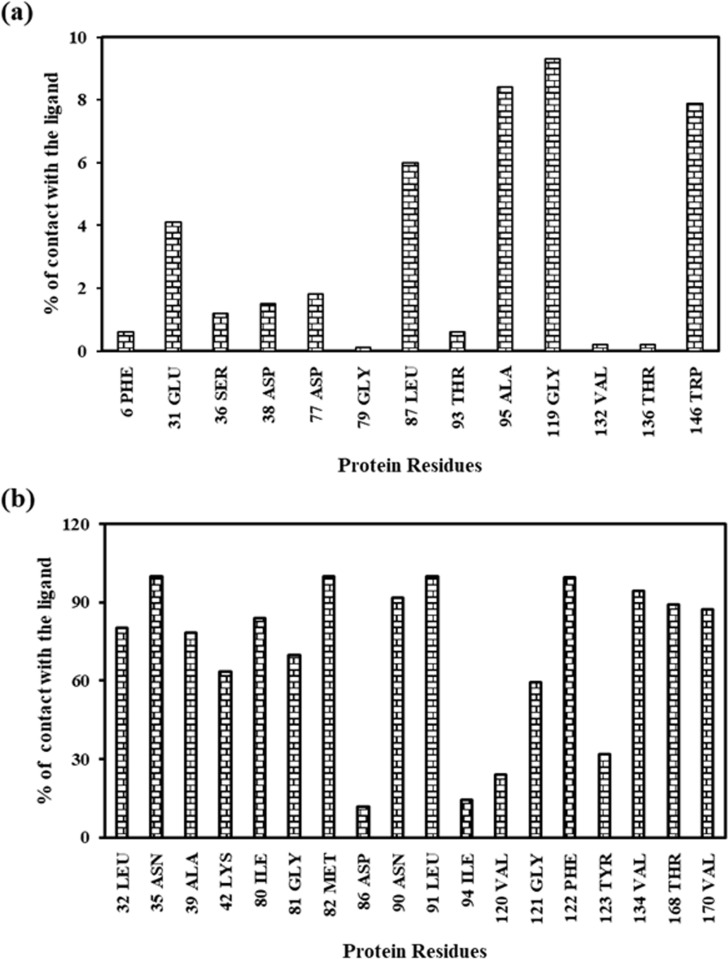
Contact map analysis. **(a)** Contact less than 10%. **(b)** contact greater than 10%.

The contact map showed other non-bonded interactions that maintained contact frequencies greater than 5%. These included Glu31, Leu167, Ala95, Gly119, and Trp146 (Fig 15). In addition, Asn35, Met82, Leu91, Phe122, Val134, Asn90, Thr168, Val170 residues exhibited higher contact frequencies with the ligand (greater than 90%).

**Fig 16:**
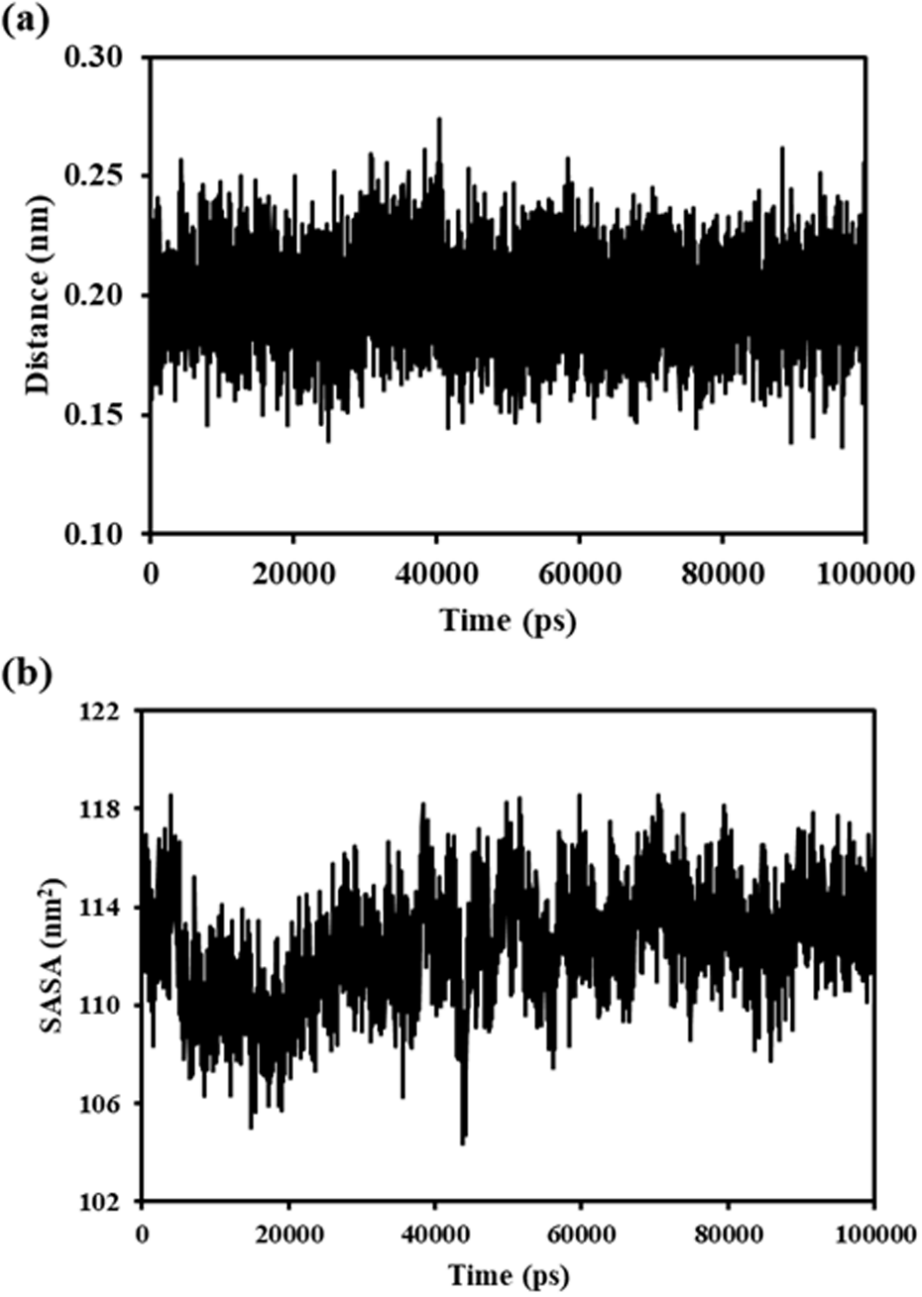
(a) Distance plot between HSP90AA1 and (-)-riboflavin (b) Solvent accessible surface area (SASA).

Throughout the MD run, the ligand and the protein showed a minimal average distance of ~ 0.20 nm (Fig 16a). The SASA plot (Fig 16b) showed a decrease from ~118 nm2–114 nm2 initially, which was maintained throughout the MD run; this infers that the complex is highly compact with the least SASA.

### *S. mannii* aqueous roots extract triggered endoplasmic reticulum (ER) stress-induced mitochondrial apoptosis in HeLa cells

**Fig 17:**
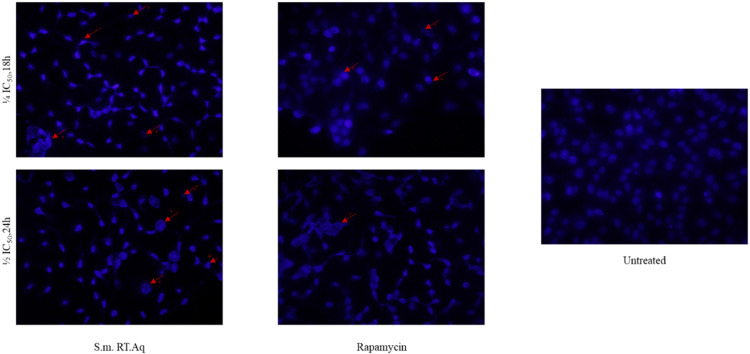
Effect of *S. mannii* aqueous roots extract on Hela cells nuclear morphology using DAPI under fluorescent microscopy, 40X. S.m. RT. Aq: *Solanecio mannii* aqueous roots extract.

Herein, we investigated the molecular mechanisms underlying the pro-apoptotic potential of the extract. First, we examined the potential of the extract to induce apoptosis-related morphological hallmarks through DAPI staining, at a dosage of ¼ IC_50_ for 18h and ½ IC_50_ for 24 hours. Our study depicted apoptosis-related morphological changes in HeLa cells upon treatment with *Solanecio mannii* aqueous roots extract which intensified in a dose- and time-dependent manner (Fig 17), including bright and condensed chromatin (arrow **1**), condensation of nuclear chromatin into sharply delineated masses, accompanied by a reduction in the circularity/nuclear shape index (arrow **2**), disrupted nuclear membrane due to membrane lysis or rupture, resulting in nucleocytoplasmic mixture (arrow **3**), DNA fragmentation (arrow **4**), ring-like structure (arrow **5**), crescent structure with fragmentation (arrow **6**), apoptotic bodies (arrow **7**), ring-like structure with fragmentation (arrow **8**), enlarged/swollen chromatin (arrow **9**), and/or invagination or herniation (arrow **10**).

**Fig 18:**
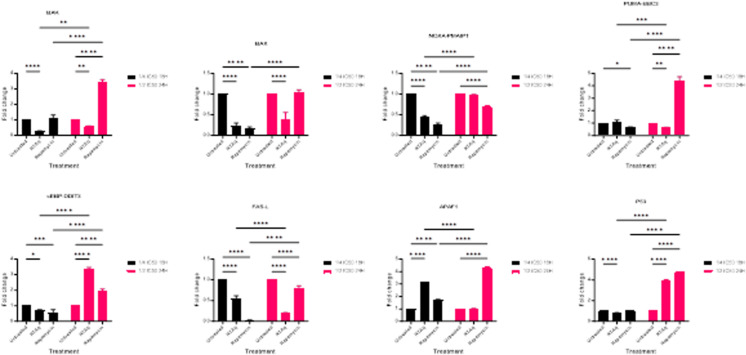
Effect of *S. mannii* aqueous roots extract on the expression of apoptosis-related genes.

Second, we investigated the effect of the studied extract on the expression level of apoptosis-related genes. Our findings indicated that *Solanecio mannii* aqueous roots extract significantly increased the expression of cEBP-DDIT3 and P53 (Fig 18), in a dose- and time-dependent manner, suggesting ER stress-induced apoptosis that does not involve Bcl-2 family members, which are implicated in the classical mitochondrial apoptotic pathway.

**Fig 19:**
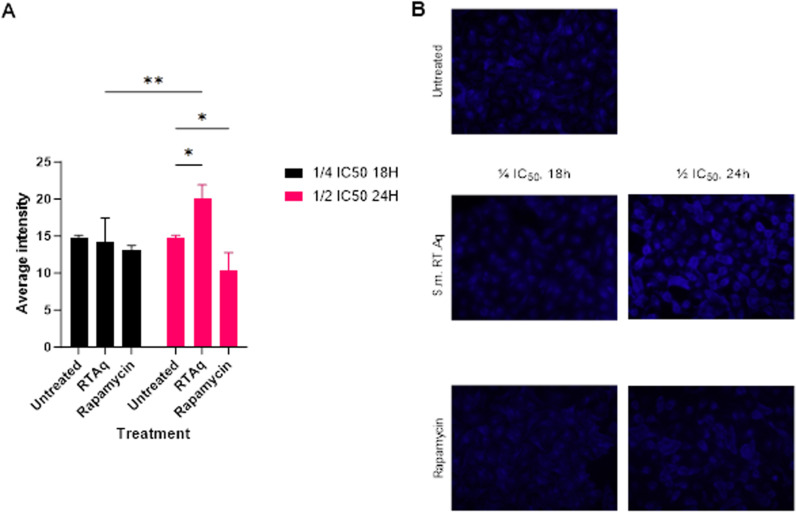
Effect of *S. mannii* aqueous roots extract on autophagosomes. **A.** MDC staining intensity. **B.** Autophagosomes stained with MDC under fluorescent microscopy, 40X. **S.m. RT. Aq:** *Solanecio mannii* aqueous roots extract.

To further confirm the induced ER stress, we performed MDC staining to analyse the formation of autophagosomes. At lower dosages, the average MDC intensity did not change significantly upon treatment; however, as the dosage increased, the average MDC intensity increased significantly, which correlated with an increased formation (upsurge) of autophagosomes in HeLa cells (Fig 19, S4 Table in S1 File).

**Fig 20:**
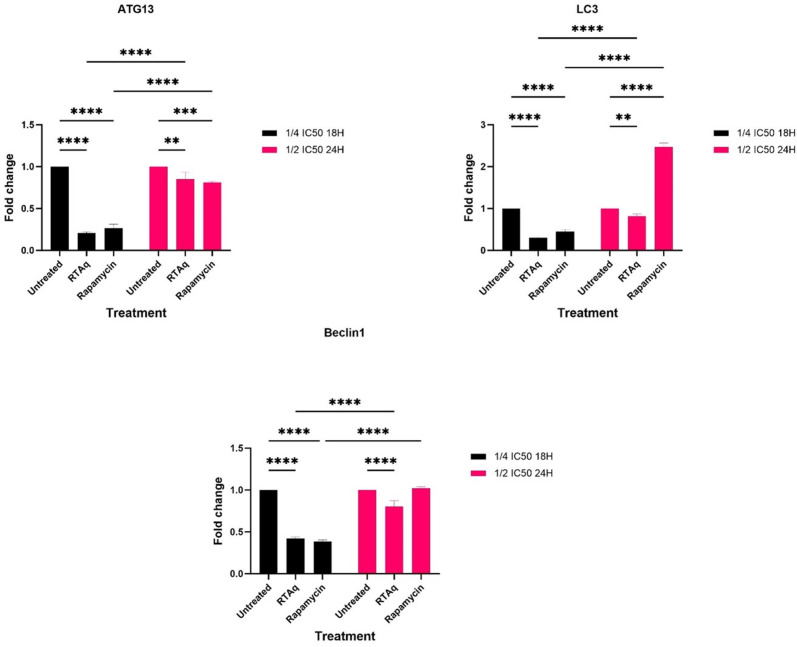
Effect of *S. mannii* aqueous roots extract on the expression of autophagy-related genes.

Additionally, we investigated the effect of *S. mannii* aqueous roots extract on the expression of autophagy-related genes (ATG13, LC3, and Beclin1). We noted a significant downregulation of autophagy-related genes upon treatment with *S. mannii* aqueous roots extract at both dosages (Fig 20).

### *S. mannii* aqueous roots extract induced cell cycle arrest in HeLa cells

**Fig 21:**
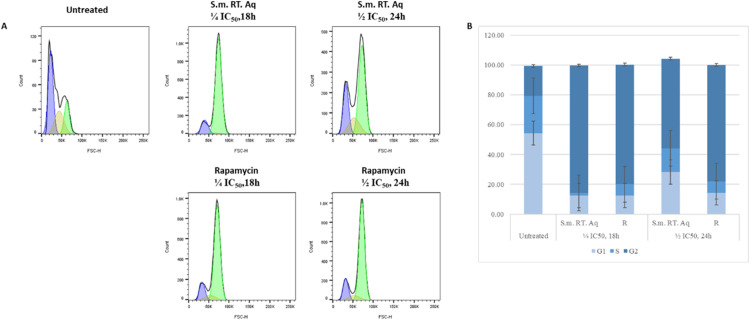
Distribution of cell cycle phases upon dose- and time-dependant treatments. **A.** cell cycle phase distribution displayed by DNA histogram. **B.** Cell cycle distribution presented as mean ± SD. **S.m. RT. Aq:** *Solanecio mannii* aqueous roots extract.

Compared to the untreated group, our findings revealed a cell cycle arrest at the G2/M phase following treatment with *Solanecio mannii* aqueous roots extract and rapamycin. The percentage of cells in the G1 phase decreased significantly in all the treatments compared to the untreated group (p < 0.0001). While the percentage of cells in phase the S decreased significantly when treated with RTAq at ¼ IC_50_ for 18h (p < 0.0001) and rapamycin (both dosages, p = 0.0002), the decrease of the percentage of cells in S phase was non-significant when treated with RTAq (p = 0.0657). Our findings show that 85.5 ± 1.14% and 60.17 ± 6.06% of HeLa cells were arrested in G2 when treated with *S. mannii* aqueous roots extract at ¼ IC_50_ for 18h and ½ IC_50_ for 24h, respectively, compared to 20.1 ± 12.39% of HeLa cells in G2 when untreated (p < 0.0001). Similarly, rapamycin, the positive control, induced a cell cycle arrest with 80.34 ± 1.45% and 78.1 ± 1.22% of HeLa cells in the G2/M phase (Fig 21 and Table 10). Our findings suggest that *Solanecio mannii* aqueous roots extract impedes the progression of the cell cycle at the G2/M checkpoint, resulting in HeLa cells accumulation at the G2 phase (S5 Table in S1 File).

**Table 10:** Time- and dose-dependent post-treatment cell cycle analysis.

	Untreated	*S. mannii* RT. Aq		Rapamycin	
		¼ IC_50_ for 18h	½ IC_50_ for 24h	¼ IC_50_ for 18h	½ IC_50_ for 24h
**G1**	54.33 ± 7.31	12.53 ± 1.30	28.27 ± 2.09	12.53 ± 1.58	14.37 ± 2.49
**S**	25 ± 6.13	1.74 ± 0.37	15.87 ± 2.97	7.48 ± 0.47	7.65 ± 1.34
**G2**	20.1 ± 12.39	85.5 ± 1.14	60.17 ± 6.06	80.34 ± 1.45	78.1 ± 1.22

Data is presented as mean (mean of percentage, %) ± SD. *S. mannii* RT. Aq: *Solanecio mannii* aqueous roots extract.

**Fig 22:**
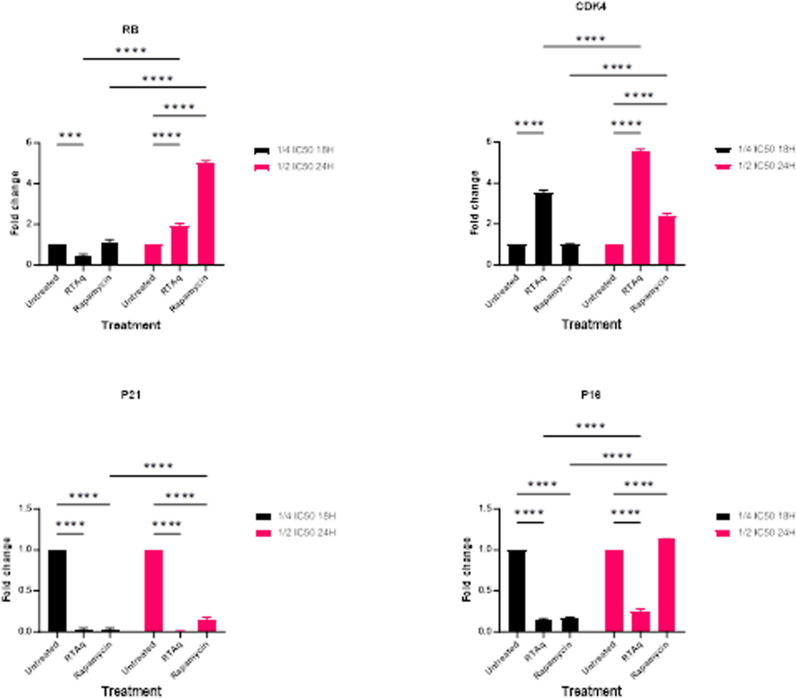
Effect of *S. mannii* aqueous roots extract on the expression of CDK4, RB, P21, and P16.

We observed a significant elevation in the expression of CDK4, and RB proteins. Concurrently, there was a noticeable depletion in the expression of the cell cycle inhibitors P21 and P16 (Fig 22).

### Effect of *S. mannii* aqueous roots extract on HPV-related proteins

**Fig 23:**
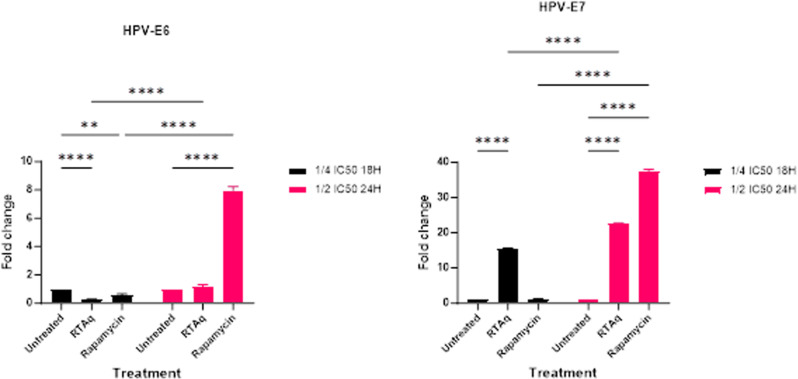
Effect of *S. mannii* aqueous roots extract on the expression of genes related to HPV oncogenic proteins (E6 and E7).

We investigated the effect of *Solanecio mannii* aqueous roots extract on the expression of E6 and E7 HPV-related genes. Upon treatment of HeLa cells, our study revealed that the expression levels of HPV E6 remained unchanged (Fig 23).

## Discussion

The incidence of cervical cancer is increasing globally, especially in low and middle-income countries. In 2020, there were 604,127 new cases and 341,831 fatalities worldwide [14]. Cervical cancer is the most common cancer in women in sub-Saharan Africa, the second most common cancer in Northern Africa after breast cancer, and the leading cause of cancer-related deaths in Eastern Africa among females [5,15]. Transitioning countries have higher rates due to socioeconomic factors, poverty rates, and access to HPV vaccination. Cervical cancer therapeutics offer a variety of approaches, including radiation, chemotherapy, and surgery. Immunotherapies and targeted medicines are promising forms of treatment. Monoclonal antibodies and immune checkpoint inhibitors offer promising strategies for treating cervical cancer, increasing patients quality of life and survival rates [16,17]. However, these therapeutic strategies face significant challenges due to side effects and drug resistance. Common side effects include fatigue, nausea, vomiting, diarrhoea, hair loss, skin irritation, and infections. In addition, several mechanisms contribute to drug resistance including mutations, evading apoptosis (mediated by upregulating anti-apoptotic proteins and downregulating pro-apoptotic proteins), increased efflux of drugs (mediated by the overexpression of ATP-binding cassette (ABC) transporters), and increased expression of genes implicated in DNA repair mechanisms. Moreover, the tumour microenvironment factors, including hypoxia, play a crucial role in drug resistance and cell survival [18].

Phytotherapy has shown promise in treating cancer by integrating ethnobotanical knowledge with modern drug discovery techniques. The medicinal properties of traditional plants have led to the identification of bioactive compounds with anticancer properties, including alkaloids, flavonoids, terpenoids, and phenolics, which demonstrate anticancer activities through various mechanisms. A notable paradigm shift in cancer treatment strategies has been the transition from traditional monotargeted approaches to the exploration of herbal-based extracts and formulations [19,20]. Multi-component herbal medicines have gained attention for their potential therapeutic benefits in cancer management due to their ability to modulate multiple targets and pathways simultaneously by acting on various molecular mechanisms, including inhibiting proliferation, inducing apoptosis, blocking angiogenesis, and boosting the immune system to disrupt the complex network of molecular pathways involved in cancer. Unlike monotargeted therapies, herbal-based treatments offer a unique advantage by presenting a diverse array of bioactive compounds that act synergistically to exert multi-component, multi-target, and multi-pathway therapeutic potential, thereby enhancing treatment efficacy, overcoming drug resistance, and mitigating the heterogeneity of cancer cells within tumours. Therefore, integrating ethnobotanical knowledge with drug discovery pipelines accelerates the identification and development of new multi-target, multi-pathway anticancer extracts, aimed at addressing these challenges and improving outcomes for cervical cancer patients. In this context, network pharmacology serves as an effective method for multi-target, multi-pathway drug discovery. It predicts potential interactions between drugs and various targets within biological networks, guiding the identification of key pathways and molecular targets involved in disease pathogenesis. Moreover, network pharmacology takes into account the diversity of disease phenotypes by capturing the various molecular mechanisms underlying disease development and progression. In this study, we investigated the “multi-compound, multi-target, multi-pathway” anti-cervical cancer potential of the aqueous extract of *Solanecio mannii* (*S. mannii*) roots by integrating computational and experimental methods, thereby elucidating the underlying molecular mechanisms.

The findings of this study revealed the complex molecular mechanism behind the selective cell inhibition and cytotoxicity recorded against human cervical cancer cell lines (HeLa cells) using the CCK8 assay. The findings indicated that the drug-like compounds identified in *Solanecio mannii* aqueous roots extract targeted 493 genes/proteins associated with cervical cancer, including NFKB1, STAT3, HIF1A, ESR1, PIK3CA, PPARG, HSP90AA1, and HSP90AB1, which were among the top 30 core targets. This study indicated that treatment of HeLa cells with *Solanecio mannii* aqueous roots extract downregulated the expression of NFKB1 in a dose- and time-dependent manner. In cervical cancer, NFKB1, induced by E6-HPV, regulates the expression of proteins implicated in inflammation (IL-6, IL-8, TNF-α, and CCL2), cell survival and evading apoptosis (Bcl-2 and Bcl-xL anti-apoptotic proteins, cIAP1, cIAP2, and XIAP inhibitors of apoptosis proteins, and survivin), proliferation (Cyclin D1 and Myc), energy metabolism and metabolic switch (glucose transporters and hexokinases), tumour microenvironment (matrix metalloproteinases, Vascular Endothelial Growth Factor/VEGF), and adhesion and migration (Intercellular Adhesion Molecule 1/ICAM-1 and integrins) [21]. Therefore, targeting NFKB1 is a promising approach for the management of cervical cancer.

Currently available strategies targeting NFKB1 signalling pathway included targeting NFKB1 subunits using the Nuclear Factor-kappa B Essential Modulator (NEMO)-Binding Domain (NBD) peptide (inhibits the activation of NFKB by blocking the interaction between NEMO (IKKγ) and the IKK complex); dehydroxymethylepoxyquinomicin, which inhibits NFKB1 DNA-binding activity, preventing its transcriptional activity, and IKK inhibitors (BAY 11-7082 and BMS-345541). However, they are associated with immunosuppression and increased infection risk [22]. Other targets include proteasome inhibitors (Bortezomib) to prevent the degradation of IκBα and thus the activation of NFKB which is associated with hematologic toxicity, cardiotoxicity, and neuropathy. RNA interference (RNAi), short hairpin RNAs (shRNAs), and antisense oligonucleotides are other approaches to knock down NFKB1 expression, often associated with off-target effects, non-specific suppression of other genes, and unintended innate immune stimulation. Additionally, these NFKB1 targeting strategies are associated with drug resistance mechanisms, including drug efflux, compensatory pathways, and delivery challenges [22,23].

Our findings indicated that compounds of *Solanecio mannii* aqueous roots extract offer a promising strategy for the management of cervical cancer through targeting NFKB1. Interestingly, the multi-component, multi-target, multi-pathway analysis indicated that NFKB1 was targeted by all the drug-like components of the extract (except chlorogenic acid), with strong binding scores. Our study indicated a strong (the strongest) binding affinity between NFKB1 and (-)-riboflavin with a binding score of -7.6 Kcal/mol. The interaction involved Van der Waals bonds (Cys C:105, Leu C:104, Asp C:103, Ala C:102, Asp C:94, Phe C:113, Lys C:93, Asn C:115, Glu E:222, Lys E:221, Ile E:224, and Glu E:224), conventional hydrogen bonds (Arg C:108, Gln E:241, Gln C:114, Ser C:112, and Tyr C:100), three unfavourable donor-donor bonds, carbon hydrogen bond (His C:111 and Arg C:108), Pi alkyl and Pi-sigma bonds with Phe E:239. The interaction pattern involved residues in chains C and E (P65), involved in P65 structural maintenance and NFKB1 complex formation and integrity. In addition, Lys C:93, Asp C:94, His C:111, Arg C:108, and Glu E:222 are involved in the regulation of NFKB activation, and the recruitment of coactivators/corepressors [24]. Collectively, the residues involved in NFκB1 p65 binding to (-)-riboflavin are implicated in the regulation of DNA binding, protein-protein interactions, activation of NFKB1, dimerization, and interactions with coactivators/corepressors [25].

These functions collectively contribute to the regulation of gene expression and cellular responses mediated by the NFKB pathway in cervical cancer. Similar binding affinity (-7.6 Kcal/mol) was recorded when NFKB1 bound to its inhibitor (sulfasalazine), involving one unfavourable positive-positive bond (Lys F:402), Van der Waals bonds (Val F:509, Asp F:506, His F:407, Thr F399, Met F:505, Gln F:501, Asn F:403, His F:470, Gln F:500, Glu F:504, and Gly F:401), carbon hydrogen bond (Asp F:506, His F:405, Gln F:500, Glu F:504, and Asn F:400), Pi-cation interaction (Lys F:402), amide-Pi stacked interaction (Asn F:400), and Pi-alkyl interaction with His F405. This interaction pattern involved residues of chain F (P105) implicated in maintaining the structural integrity of P105, crucial in the stabilization and folding of the protein, and importantly its processing into P50 [24].

Similarly, HIF1A was one of the core targets and enriched pathways of the aqueous extract of *Solanecio mannii* roots in exerting its therapeutic activity against cervical cancer. HIF1A plays a role in tumorigenesis and cancer progression. An increased level of expression of HIF1A is associated with tumour’s adaptation to low oxygen levels and hypoxia, promoting the Warburg effect by upregulating glycolytic enzymes and glucose transporters (Lactate Dehydrogenase A/LDHA and GLUT1), and increasing migration and angiogenesis potential of cancer cells (through the upregulation and production of VEGF and matrix metalloproteinases) [26–28]. Current strategies in targeting HIF1A include direct inhibitors of its synthesis (PX-478, small interfering RNA or short hairpin RNA), inhibition of its DNA-binding, or its dimerization, indirect inhibitors, and inhibitors of metabolic pathways, including 2-Deoxy-D-glucose, a glucose analogue, that inhibits glycolysis and reduces the Warburg effect driven by HIF1A. Organ and haematological toxicities, metabolic changes (lactic acidosis and low blood pressure), and drug resistance are often associated with these drugs [29]. Our study indicated that treatment of HeLa cells with *S. mannii* aqueous roots extract downregulated the expression of HIF1A in a dose- and time-dependent manner. At a proteomic level, the molecular docking analysis indicated the strongest binding affinity of HIF1A with (-)-riboflavin and thymidine-5′-triphosphate with a binding affinity of -6.8 Kcal/mol. The interaction pattern between HIF1A and (-)-riboflavin involved one unfavourable donor-donor interaction with Arg B:170, Van der Waals bonds (Gly B:298, Trp B:189, Gln B:299, Ile B:227, Thr B:322, His B:229, and Ser B:330), carbon hydrogen bond with Lys B:190, Pi sigma bond (Tyr B:325), Pi-Pi stacked, Pi-sigma, amide-Pi stacked and Pi-alkyl bounds with Tyr B:325 and Lys B:297. These residues are implicated in maintaining the structural stability of HIF1A, binding stability, ligand specificity and stabilization, as well as its dimerization [30,31]. Therefore, (-)-riboflavin could be a potent inhibitor of HIF1A through these interactions, which can effectively reduce hypoxia-induced gene expression.

Similarly, the thymidine-5′-triphosphate-HIF1A complex exhibited an interaction characterized by repulsive positive-positive and donor-donor bonds with Lys B:190 and Arg B:170, Van der Waals bonds (Val B:191, Trp B:189, Gly B:298, Gln B:299, Asn B:232, Ser B:231, and Pro B:228), conventional hydrogen bonds (Lys B:190, Thr B:176, and Ile B:227), Pi-cation bond with His B:229, and Pi-sigma and Pi-Pi stacked bonds with Tyr B:325. The repulsive force, due to the unfavourable interactions, induces HIF1A disruption and destabilisation. In addition, this interaction hinders HIF1A-specific ligands/binders binding in the binding pocket, thereby inhibiting HIF1A’s function, blocking the binding/active site, and inducing conformational changes. The molecular docking of HIF1A and its inhibitor (PX-478) revealed a binding affinity of -5.3Kcal/mol. The interaction involved Van der Waals bonds (Gly B:298, Lys B:297, Val B:191, Ile B:227, Cys B:173, and Thr B:188), conventional hydrogen bond (Arg B:170, Trp B:189, and Lys B:190), Pi-cation, Pi-Pi stacked, and Pi-alkyl with His B:229. PX-478 exerts its inhibitory effect through binding to the binding pocket/active site, thereby inducing a conformational change and blocking HIF1A’s functional site.

During hypoxia, HIF1A directly activates the peroxisome proliferator-activated receptor (PPARG) [32], a transcription factor that was shown to be downregulated upon treatment with *S. mannii* aqueous roots extract in a dose- and time-dependent manner, probably due to: 1) the depletion of HIF1A and/or 2) a direct downregulation effect exhibited by the extract. PPARG is implicated in metabolism reprogramming, increased ATP levels, and enhanced mitochondrial biogenesis activities, thereby favouring cell proliferation, growth, survival, and metastasis [33]. In addition, PPARG expression enhances immunosuppression and modulates the tumour microenvironment [34]. Our findings showed that PPARG was targeted by (-)-riboflavin and 2-(4-isobutylphenyl) propionic acid with a binding affinity of -7.3 and -7.1 Kcal/mol, respectively. The molecular docking showed the highest binding affinity was recorded when PPARG interacted with L-beta-homotryptophan, with a binding affinity of -7.7 Kcal/mol, compared to -6.0 Kcal/mol when it interacted with its inhibitor (mifobate). The interaction between PPARG and L-beta-homotryptophan involved Cys B:314, Gly B:313, Ile B:310, Met B:377, Ile B:370, Arg B:317, Phe B:293, His B:295, Lys B:294, Ile B:296, Ser B:371, Glu B:320 and Phe B:316. These residues are crucial for maintaining the conformation of the ligand-binding domain of PPARG, thereby hindering ligand binding and PPARG activation [35]. Therefore, L-beta-homotryptophan stabilizes the inactive conformation of PPARG necessary for DNA binding and transcriptional activation of target genes. Mifobate was shown to interact with PPARG via Ser A:273, Leu A:448, Leu A:240, Gln A:444, Ser B:458, Gln B:459, Lys B:451, Pro B:455, Ser B:457, Leu B:460, Leu B:450, and Phe B461. Attractive electrostatic interactions were detected when Mifobate interacted with Glu A:447 and Glu B:447, preventing PPARG from transitioning to the active conformation. The aqueous extract of *S. mannii* roots was found to target the lipolysis pathway (hsa04923) and PPAR signalling pathway (hsa03320), where PPARG regulates triacylglycerol (TAG) synthesis and facilitates the uptake of fatty acids by upregulating fatty acid binding proteins FABP3, 7, and 4 during hypoxia [36,37]. In this study, FABP4 was also targeted by *S. mannii* aqueous roots extract.

Notably, PPARG plays a context-dependent role in cancer by playing both oncogenic and tumour suppressor roles. In cervical cancer, overexpression of PPARG is implicated in tumourigenesis, cell growth and survival, proliferation and drug resistance in cervical cancer cell lines (HeLa, SiHa, and Me180). Inhibition of PPARG has been shown to trigger apoptosis, induce G2/M arrest and mitotic catastrophe, and reduce the migratory and invasive potential of pancreatic, colorectal, oesophageal, hepatocellular, and cervical carcinomas [38,39]. Monotherapies targeting PPARG are not clinically used due to their ineffectiveness.

Furthermore, this study indicated that oestrogen receptor alpha (ERα), encoded by ESR1 gene, was targeted by the studied extract, specifically by caffeic acid, which showed a binding affinity of -6.5 Kcal/mol, involving eight Van der Waals interactions with Met A:338, Leu A:384, Leu A:346, Thr A:347, Met A:343, Leu A:525, and Leu: 349; one conventional hydrogen bond with Glu A:353; Pi-Pi T-shaped bond with Phe A:404; and three Pi-Alkyl bond with Ala A:350, Leu A:387, and Leu A:391. Caffeic acid displayed a stronger interaction with ESR1, compared to ESR1 inhibitor (Elacestrant), which showed a binding affinity of -6.4 Kcal/mol. Notably, the molecular docking study showed L-beta-homotryptophan and 2-(4-isobutylphenyl) propionic acid displaying the strongest binding affinities with ESR1 (-7.4 and -7.1 Kcal/mol, respectively). Both intra-interaction complexes involved Glu A:353, Arg A:394, Leu A:384, Ile A:424, His A:524, Leu A:525, Met A:421, and Leu A:346 with Van der Waals bonds. When interacting with L-beta-homotryptophan, the interaction implicated five more residues in Van der Waals bonds (Met A:388, Leu A:428, Gly A:521, Thr A:347, and Leu A:349). Amino acid residues Trp A:383, Met:343, and Leu A:346 of ESR1 were implicated in Van der Waals bonds only when interacting with 2-(4-isobutylphenyl) propionic acid. While it was involved in the Van der Waals bond when interacting with L-beta-homotryptophan, the residue Met A:388 was implicated in alkyl/Pi-alkyl interaction with 2-(4-isobutylphenyl) propionic acid, in addition to Ala A:350, Phe A:404, Leu A:387, and Leu A:391. Phe A:404 was involved in a Pi-Pi T-shaped bond when ESR1 interacted with L-beta-homotryptophan. Being part of the ligand-binding domain, these residues are particularly involved in ligand binding to ESR1, conformational changes upon ligand binding, and ESR1 dimerization and activation [40].

These findings demonstrated that caffeic acid, L-beta-homotryptophan, and 2-(4-isobutylphenyl)propionic acid are potent inhibitors of ESR1 and effectively modulate its activation. The oestrogen receptor, upon binding to oestrogen, functions a transcription factor that regulates genes involved in cell cycle progression, proliferation, and survival, all of which contribute to tumorigenesis and cancer progression. On one hand, activation of oestrogen signalling pathway have been shown to upregulate c-MYC, and Cyclin D1, thereby promoting the G1/S transition. This observation aligns with our cell cycle analysis, where no arrest was detected at the G1 phase despite the overexpression of RB1. On the other hand, oestrogen stimulates the transcription of HPV-related proteins. Substantial evidence indicates that HPV-associated cervical carcinomas express high levels of ERα, which in turn mediates the MAPK/ERK signalling pathway, enhancing the invasive, migratory, and proliferative potential of cervical cancer cell lines, including CaSki and HeLa [41–43]. Moreover, the overexpression of the 17β-hydroxysteroid dehydrogenase type 1 (HSD17B1) in HeLa, SiHa, and CaSki, indicates the ability of HPV-positive cervical cancer cells to convert estrone to oestrogen locally [43,44]. The oestrogen signalling pathway (hsa04915) was among the KEGG pathways implicated in the therapeutic activity of *S. mannii* aqueous roots extract, targeting 23 genes associated with cervical cancer. Notably, ESR1 expression was transcriptionally downregulated by the extract in a time- and dose-dependent manner.

Our study demonstrated the ability of *S. mannii* aqueous roots extract in targeting autophagy (hsa04140) in cervical cancer. Our findings indicated a potent inhibition of autophagy as evidenced by significant downregulation of genes involved in autophagosome formation, maturation, and fusion with lysosomes (ATG13, Beclin1, and LC3). *S. mannii* modulated autophagy primarily at the nucleation and maturation stages at a lower dosage, as indicated by reduced autophagosomes staining intensity. However, HeLa cells adapt to the increased dosage by forming more autophagic vacuoles, as reflected by an increase in MDC staining. Nevertheless, the relative expression of ATG13, Beclin 1, and LC3 is persistently significantly reduced, demonstrating a stronger regulatory effect of *S. mannii* aqueous roots extract on autophagosomes fusion with lysosomes, thereby an incomplete or impaired autophagy. This study indicated that treatment of HeLa cells with *S. mannii* aqueous roots extract inhibits the degradation and recycling of damaged proteins and organelles mediated by autophagy, thereby leading to accumulation, cellular stress, and activation of stress response pathways, which ultimately trigger apoptotic cell death, evidenced by chromatin condensation and other apoptosis-associated nuclear changes.

The upregulation of APAF-1 in HeLa cells upon treatment with the extract at a lower dosage indicated the activation of the intrinsic apoptotic pathway, suggesting that inhibition of autophagy and resultant cellular stress activated stress-induced apoptotic signalling. Additionally, at a lower dosage, HSP90AA and HSP90AB were both downregulated, which suggested impairment in protein homeostasis and contributed to cellular stress through the impaired folding of functional proteins. HSP90AA and HSP90AB were targeted by several phytocompounds, including glycyl-L-proline, 1-decanoyl-2-hydroxy-sn-glycero-3-phosphocholine, D-fructose, L-sorbopyranose, thymidine-5`-monophosphate, and thymidine-5`- triphosphate sodium salt. The molecular docking study indicated that (-)-riboflavin is an effective inhibitor of HSP90AA1 and HSP90AB1 with binding affinities of -9.6 and -7.9 Kcal/mol, respectively. The interaction involved attractive charges with Asp A:93, conventional hydrogen bond with Leu A:48, Pi-sulfuric bond with Phe A:138, and other Van der Waals, alkyl, and Pi-alkyl intra-complex bonds to strengthen the interaction between HSP90AA1 and (-)-riboflavin. Notably, the interaction between HSP90AB1 and (-)-riboflavin implicated the D chain, and was characterized by the formation of an unfavourable electrostatic repulsion (positive-positive interaction) with Lys D:36, attractive charge with Glu C:115, four conventional hydrogen bonds (Tyr D:33, Ser D:34, Gln C:118, and Gln D:189). Additional interactions with HSP90AB1 included Van der Waals and carbon-hydrogen bond. These interactions stabilize (-)-riboflavin within the ATP-binding site, enhancing the inhibitory effect by altering the conformation of the active site [45]. (-)-Riboflavin likely disrupted the normal chaperone function of HSP90AA1 and HSP90AB1, essential for protein folding. The interaction between HSP90 proteins and their inhibitor, Geldanamycin, demonstrated comparable binding interactions with a binding affinity of -7.4 and -8.5 Kcal/mol when interacting with HSP90AA1 and HSP90AB1, respectively.

In addition, phosphatidylinositol-4,5-bisphosphate 3-kinase catalytic subunit alpha (PIK3CA) and signal transducer and activator of transcription 3 (STAT3), involved in cell survival and proliferation pathways, were significantly downregulated in HeLa cells when treated at a lower dosage of the extract. KEGG pathway enrichment analysis revealed that PI3K-Akt (hsa04151) and JAK-STAT (hsa04630) signalling pathways, enriched with 54 and 21 targeted genes, respectively, are among the pathways targeted by *S. mannii* aqueous roots extract in exerting its therapeutic effect against cervical cancer cells. PIK3CA, implicated in PI3K-Akt and other pathways, was targeted by glycyl-L-proline, galactopyranose, myo-inositol, cerulenin, and farnesol, whereas STAT3, implicated in several enriched pathways, targeted by *S. mannii*, was targeted solely by glycyl-L-proline. With increasing dosage, the apoptosis-related nuclear morphological changes intensified, involving endoplasmic reticulum (ER) stress response.

Our findings suggested that HeLa cells experienced severe ER stress resulting from the accumulation of unfolded proteins, non-recycled biomolecules, and damaged organelles, as a result of autophagy inhibition. Additionally, HeLa cells significantly increased the level of expression of autophagy-related proteins, the formation of autophagosomes, heat shock proteins, PIK3CA, and STAT3 as cytoprotective compensatory approach to counteract heightened cellular stress. Though this compensatory activation eventually contributes to further cellular stress and DNA damage, as evidenced by the upregulation of cEBP/DDIT3 and p53, respectively. At this point, the cellular response shifts to a non-transcriptional non-classical P53 pathway, where p53 can translocate to the mitochondria directly and interact with mitochondrial proteins to trigger apoptosis independently of pro-apoptotic factor expression (NOXA, PUMA, BAK, BAX, and APAF1). Moreover, cEBP/DDIT3 overexpression indicates both elevated oxidative stress and prolonged ER stress within the cell, which marks a shift of the cellular response to apoptosis. Our findings suggested an interplay between the p53 pathway (resulting from DNA damage) and the ER stress pathway (resulting from cellular stress) in promoting apoptosis in HeLa cells upon treatment, including oxidative stress amplification, direct mitochondrial effects, crosstalk between the ER and mitochondria, and eventually, cell death when autophagy is insufficient in mitigating the severe induced stress and DNA damage [46–50].

Upon treatment, HeLa cells were arrested in the G2/M cell cycle phase. The study suggested that inhibiting autophagy and subsequent cellular stress at lower drug dosages can lead to the buildup of reactive oxygen species (ROS), resulting in DNA damage and subsequent cell cycle arrest. APAF-1 upregulation further indicates apoptotic signalling resulting from DNA damage, which can also contribute to G2 arrest. Upon induction of p53, mediated by DNA damage, and CHOP activation via ER stress, arresting the cell cycle could occur via several mechanisms. Notably, P53 can induce the expression of P21, a cyclin-dependent kinase inhibitor that blocks the activity of CDK1, leading to cell cycle arrest at the G2/M checkpoints, preventing cells from entering mitosis. However, our findings showed a depletion of P21 upon treatment with *S. mannii* aqueous roots extract at both dosages. Nevertheless, the cell cycle arrest in G2 can still occur through alternative mechanisms, including the growth arrest and DNA damage-inducible pathway (GADD45 and 153, also known as cEBP/DDIT3) and 14-3-3σ, both of which disrupt of the CDK1/cyclin B complex, inhibiting its nuclear translocation and thereby preventing G2/M transition.

In addition, suggested alternative pathways include cEBP/DDIT3-mediated upregulation of GADD34, which is involved in the dephosphorylation of eIF2α, and eventually cell cycle arrest [46–49,51–55]. Alternatively, a feedback loop created by calcium signalling enhances the apoptotic response mediated by DNA damage and ER stress [47]. The calcium signalling pathway (hsa04020) is one of the KEGG pathways targeted by the *S. mannii* aqueous roots extract and enriched with 42 cervical cancer-associated targets.

Upon treatment with *S. mannii* aqueous roots extract, our findings showed the extract’s potential to inhibit the expression of p16 and P21. P16 is a diagnostic marker for cervical neoplasia and essential for cell survival, indicating potential for targeted treatments, as normal cell proliferation is not required for p16 expression [56–58]. The role of p16 in cancer is cellular context dependent. Although p16 functions as a tumour suppressor in the majority of cellular contexts, the tumour suppressor role of p16 is abolished in HPV-transformed cervical malignancies due to the depletion of retinoblastoma protein (Rb) by oncoprotein E7, which is a target of the CDK4/6 kinase [59]. It has been reported that p16 demonstrates oncogenic activity in cervical carcinoma cell lines by promoting proliferation, and its silencing reduced the cell viability and proliferation of CaSki and HeLa cell lines [60]. Notably, RB protein was upregulated upon treatment with the studied extract at ½ IC_50_ for 24h despite the increased expression of CDK4 and E7 in a dose- and time-dependent manner. In accordance with cell cycle analysis results, the expression of CDK4 enabled the transition to the S phase and the replication of DNA material, however, we hypothesize that *S. mannii* extract facilitated G1/S transition, induced a replicative stress and DNA damage, and enhanced ER stress, and ultimately triggered cell cycle arrest in G2/M and cell death. The depletion of p16 and p21 might initially allow cell cycle progression, but the downstream effects of DNA damage and stress activate robust G2/M checkpoint. On the other hand, we hypothesize that HeLa cells upregulated E7 and CDK4 as a compensatory approach to counteract the cytotoxic activity of the extract. The upregulation of P53 and RB indicates the ability of HPV-positive-transformed cells of the cervix to restore tumour suppressor expression and homeostasis despite the non-significant change of the level of expression of E6 and the significant increase of E7 and downstream oncogenic pathways when treated at a higher dosage.

Overall, the pro-apoptotic effect of *S. mannii* appears to be DNA damage-mediated, which activates the DNA damage response pathways, including the tumour suppressor p53. In addition, endoplasmic reticulum stress, due the accumulation of misfolded or unfolded proteins within the ER, triggers the unfolded protein response. Initially, the UPR works to restore normal protein folding; however, prolonged or severe stress can shift the cellular response towards apoptosis. In this context, the depletion of p21 is critical. Under normal conditions, p21 inhibits CDK4 and helps arrest the cell cycle at the G1/S transition checkpoint. However, when p21 is depleted, CDK4 activity is maintained and the cell cycle progresses.

Additionally, p53, while typically inducing apoptosis through transcription-dependent mechanisms (activating PUMA and BAX), can also trigger apoptosis through a transcription-independent pathway. Despite the overexpression of RB, HeLa cells arrest in the G2 phase rather than earlier in G1 or S-phase upon treatment with *S. mannii* extract. This paradox arises due to the depletion of p21 and unchecked CDK4 activity which led to the inactivation of RB through hyperphosphorylation. Nevertheless, cumulative signals from DNA damage and ER stress activated the G2/M checkpoint control. Thus, although RB overexpression should theoretically halt the cell cycle earlier, the depletion of p21 and activation of stress-induced checkpoints lead to G2 arrest and eventual apoptosis through p53-mediated non-transcriptional mechanisms. CDC25C, CDK1, and Cyclin B are key regulators of the G2/M transition; however, changes in the expression of these genes were not incorporated in the present study due to financial constraints.

Eventually, this study revealed the multi-target and multi-pathway therapeutic activity of *S. mannii* aqueous roots multi-compound extract for cervical cancer management. Consequently, we identified the core phytocompounds through which the extract exerted its therapeutic activity against cervical cancer through the construction of multi-component, multi-target, multi-pathway interaction network. While all the phytocompounds were implicated in the therapeutic activity of the studied extract, the core pharmacodynamic substances, were glycyl-L-proline (targeting 11 core targets), and galactopyranose and N-formyl-L-methionine (targeting 10 targets, each). Based on the molecular docking study, (-)-riboflavin, thymidine 5’-triphosphate, 2-(4-isobutylphenyl) propionic acid, caffeic acid, and L-beta homotryptophan were identified as potent inhibitors of the core cervical cancer-associated targets.

This study identified NFKB1 as the most targeted protein, exhibiting a strong binding affinity with (-)-riboflavin. Additionally, the molecular docking results supported the network pharmacology findings, demonstrating significant interactions between core target proteins and drug-like compounds. Notably, the highest binding affinity was observed between HSP90AA1 and (-)-riboflavin.

With a global RMSD of 2.352 ± 0.516 Å and a ligand RMSD of 4.271 ± 0.605 Å, the RMSD values revealed that binding of (-)-riboflavin to HSP90AA1 caused conformational stability in both the overall protein and the binding pocket. The modest variations in the ligand RMSD imply that (-)-riboflavin preserves a stable but slightly dynamic contact with the protein, indicating a flexible binding mechanism within the pocket. Similar stability (RMSD of 2.336 ± 0.563 Å) was shown by the residues engaged in intermolecular interactions (22–186), so corroborating the observation that the binding area was steady across the simulation. While some areas of the HSP90AA1 protein displayed increasing flexibility (RMSF > 0.3 Å), especially residues 63–67 and 223–224, the RMSF analysis revealed that some areas of the protein remained rigid (RMSF < 0.3 Å), indicating stable structural elements. Furthermore, with minimal expansion, the radius of gyration (Rg) remained quite constant (1.749 ± 0.017 Å) across the 100 ns simulation, suggesting that the HSP90AA1 structure retained its compactness upon binding with (-)-riboflavin with a well-equilibrated system and no significant structural rearrangement. With a notable fraction of α-helices and β-sheets, the secondary structure analysis revealed that the general architecture of HSP90AA1 remained unaltered. With 0.60 total secondary structure content, the preservation of the secondary structure across the simulation emphasized the stabilizing influence of (-)-riboflavin binding on the structural organization of the protein.

Furthermore, the stability of the protein-ligand complex is emphasised by favourable van der Waals and electrostatic interactions as well as by intermolecular hydrogen bonding with residues of the active site (ALA A: 111, VAL A:136, ILE A: 110, GLY A:135, LYS A:58, SER A:52, ALA A:55, ASP A:93, LEU A:48, THR A:184, VAL A:186, ASN A:51, MET A:98, PHE A:138, VAL A:150 LEU A:103, TRP A:162, TYR A:139, and PHE A:22). The MMPBSA revealed a favourable binding free energy for the HSP90AA1-(-)-riboflavin complex (-27.38 ± 1.47 kJ/mol). The strength of the interaction between the protein and the ligand is emphasised by the major contribution from electrostatic interactions (EEL: -14671.44 ± 115.19 kJ/mol) and van der Waals forces (VDWAALS: -1597.64 ± 25.56 kJ/mol). Thus, (-)-riboflavin forms a stable complex with HSP90AA1 marked by conformational stability, strong electrostatic and van der Waals contacts, and low structural disturbance. The preserved secondary structure and compactness underline even more the structural integrity of HSP90AA1 upon ligand interaction. Riboflavin exhibits promising anticancer potential, largely due to its role as a precursor of flavin mononucleotide (FMN) and flavin adenine dinucleotide (FAD), which are essential cofactors in redox and metabolic reactions. The isoalloxazine ring undergoes reversible redox reactions and electron transfer in oxidoreductases, elevating ROS levels, and inducing apoptosis. The ribitol side chain of riboflavin plays a role in its solubility, cellular uptake, and phosphorylation into FMN and FAD. Additionally, riboflavin impacts mitochondrial metabolism and energy production and disrupts the Warburg effect in cancer cells [61–64].

Due to computational constraints, this study focused on the interaction between HSP90AA1 and (-)-riboflavin. It is worth noting that the aqueous fraction of *S. mannii* contained multiple components, each of which showed a high binding affinity with cervical cancer-associated targets, and contributed to the overall anti-cancer effect of *S. mannii* against the human cervical cancer cell line (HeLa) in vitro.

## Conclusion

The aqueous extract of *Solanecio mannii* (*S. mannii*) roots demonstrated significant potential for the treatment and management of cervical cancer through a “multi-compound, multi-target, multi pathway” molecular mechanism. It selectively targeted key proteins and pathways associated with cervical cancer, induced ER-stress, and DNA damage-mediated, transcription-independent apoptosis, inhibited hypoxia and autophagy, and arrested the cell cycle in the G2/M phase. Additionally, these findings highlighted the potential of *S. mannii* extract in synergy with conventional treatments to overcome drug resistance in cervical cancer therapy and management. While our study provides valuable insights into the molecular mechanism of *S. mannii* aqueous roots extract against human cervical cancer cells, the analysis of target effects was conducted at the transcriptomic level due to financial limitations. Further validation via Western blot is required to provide a deeper understanding at the metabolomics level.

## Supporting information

S1 File S1-35_Figs_and_S1-5_Table. (PDF)
