# Supplementary material for: Computational investigation and experimental validation of the molecular mechanism of Solanecio mannii aqueous roots extract against cervical cancer
Source: PLoS One. 2025 May 30;20(5):e0323680. doi: 10.1371/journal.pone.0323680 (PMC12124759; doi:10.1371/journal.pone.0323680)
Supplement: S1 File — (PDF) [file pone.0323680.s001.pdf]

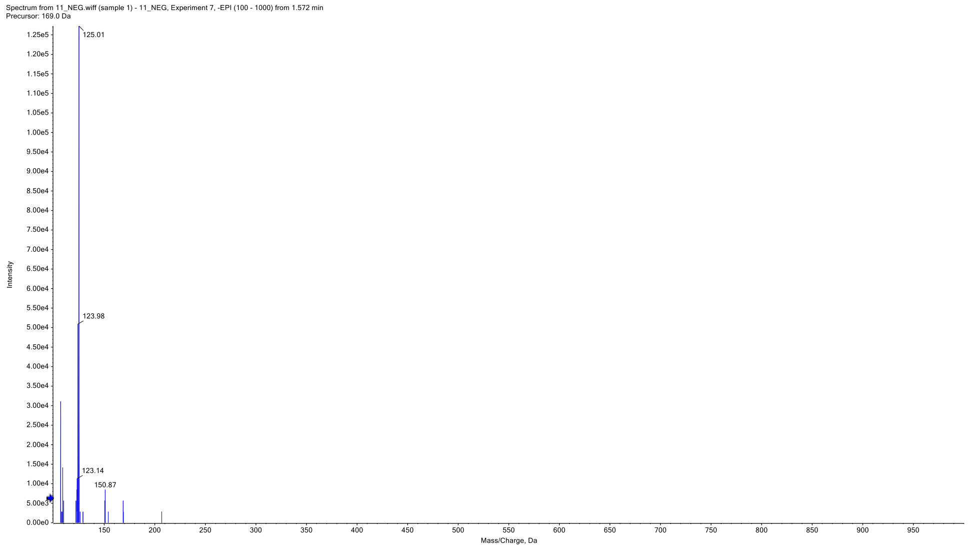

S1 Fig. Fragmentation patterns of DL-Glyceraldehyde 3-phosphate

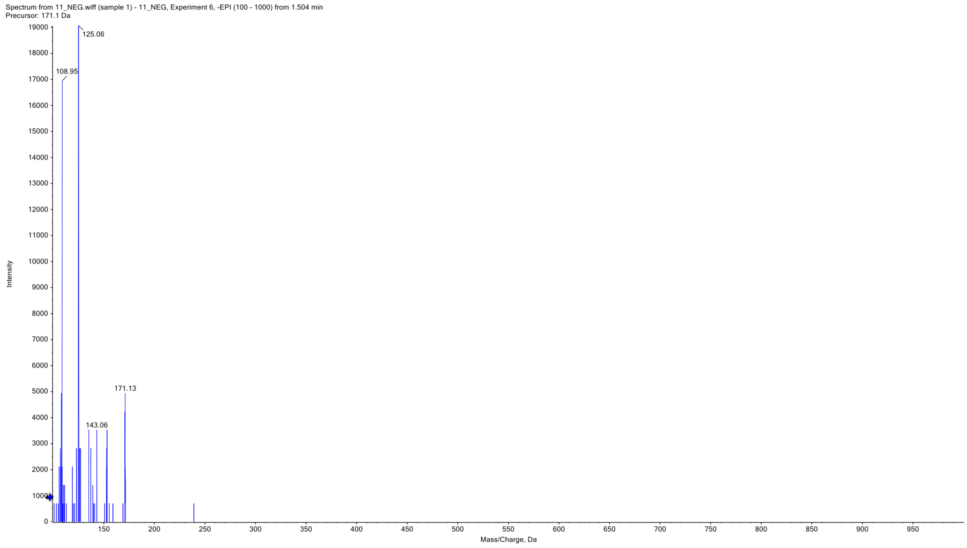

S2 Fig. Fragmentation patterns of Glycyl-L-proline

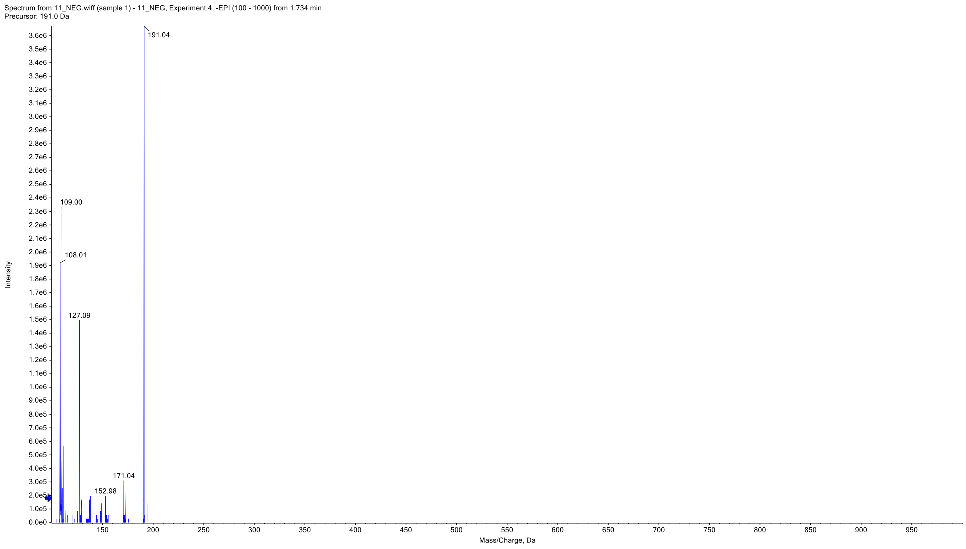

S3 Fig. Fragmentation patterns of Citrate

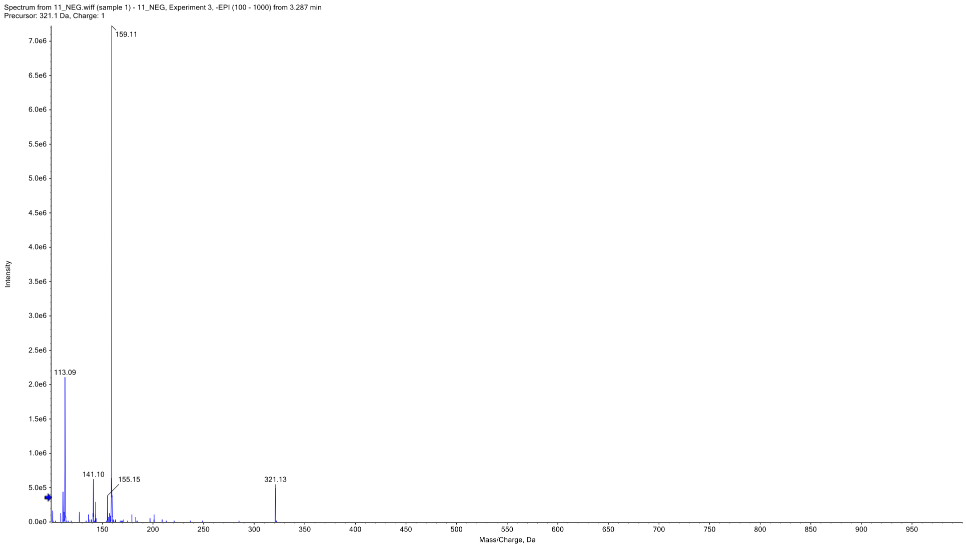

S4 Fig. Fragmentation patterns of Thymidine-5'-monophosphate

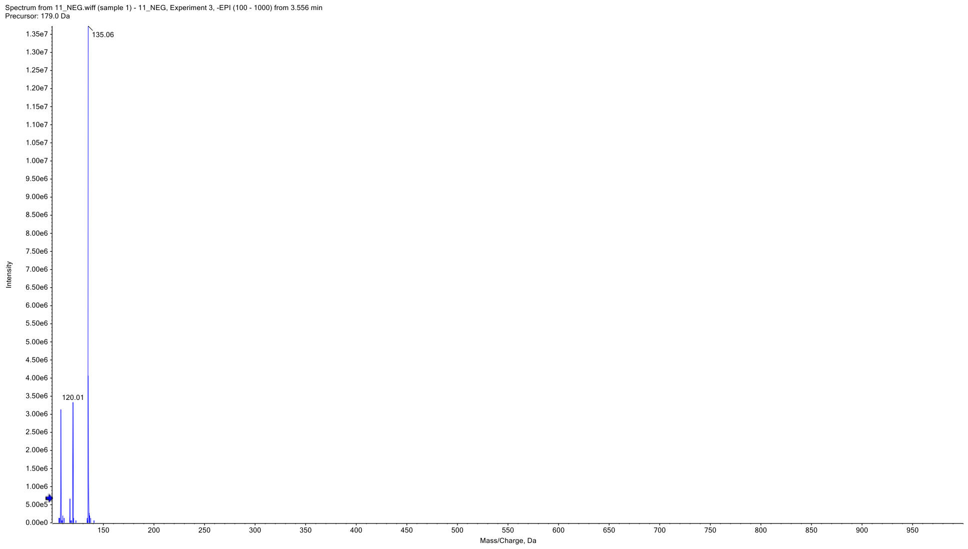

S5 Fig. Fragmentation patterns of CAFFEIC ACID

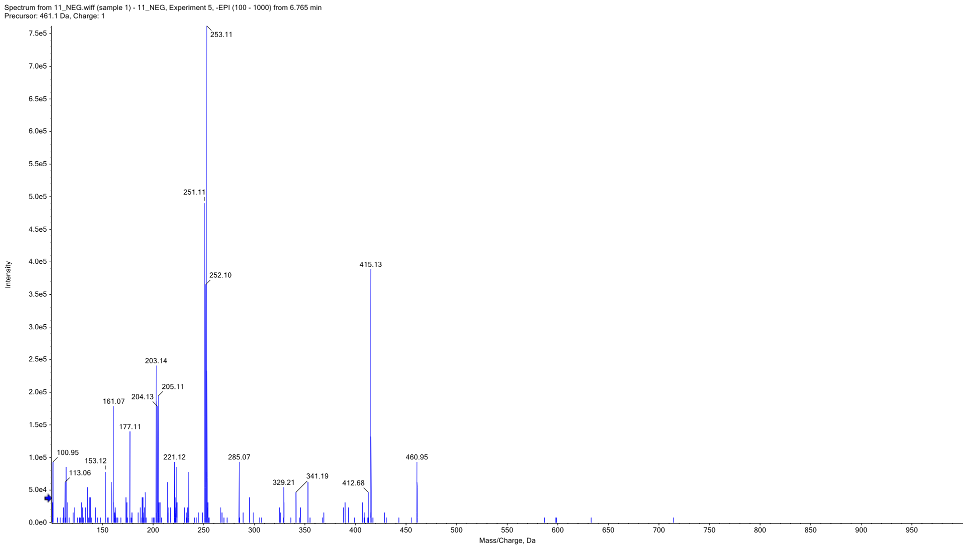

S6 Fig. Fragmentation patterns of Peonidine-3-O-glucoside chloride

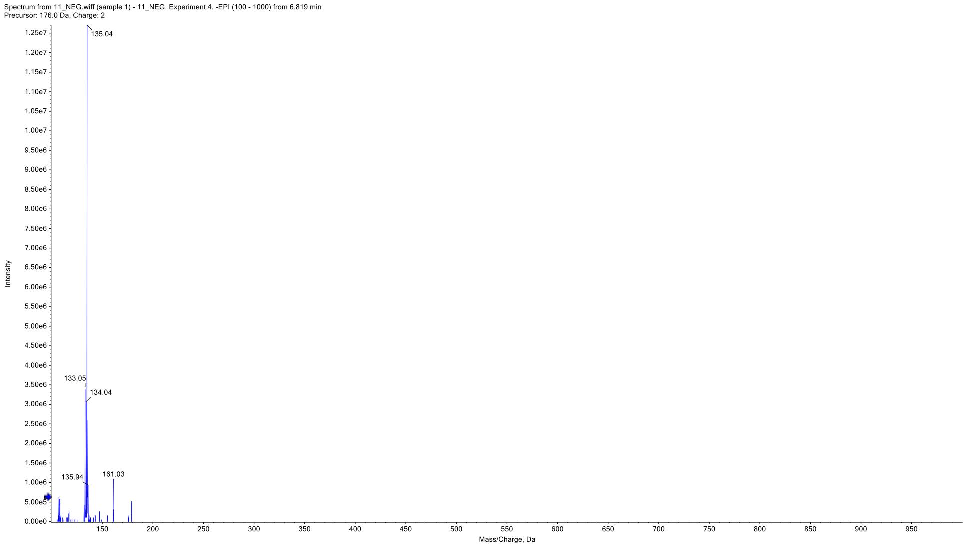

S7 Fig. Fragmentation patterns of N-Formyl-L-Methionine

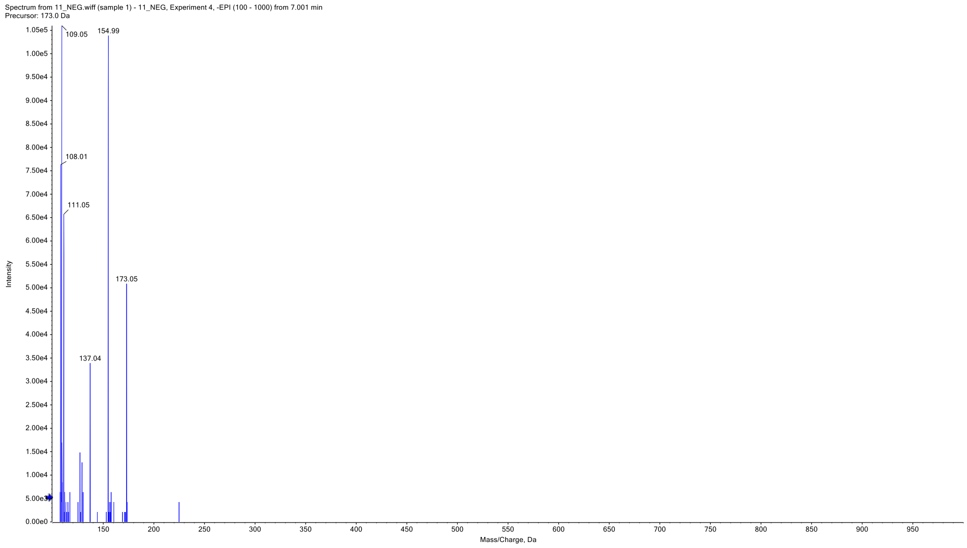

S8 Fig. Fragmentation patterns of cis-Aconitate

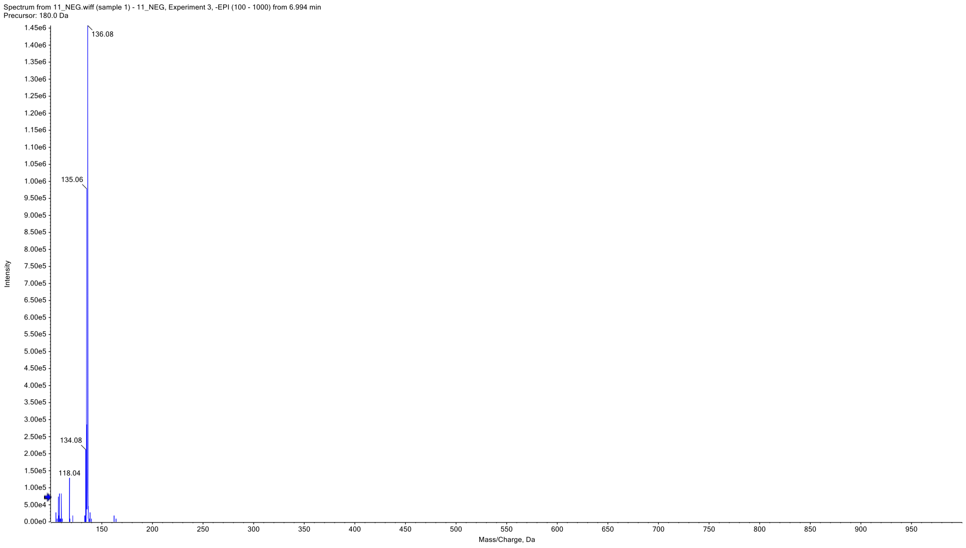

S9 Fig. Fragmentation patterns of L-Methionine sulfone

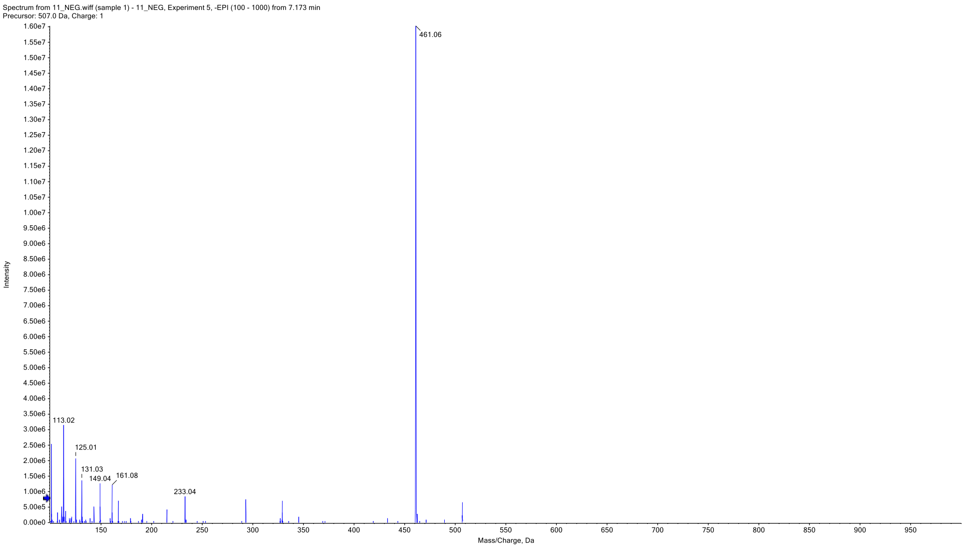

S10 Fig. Fragmentation patterns of Inosine-5'-triphosphate trisodium salt

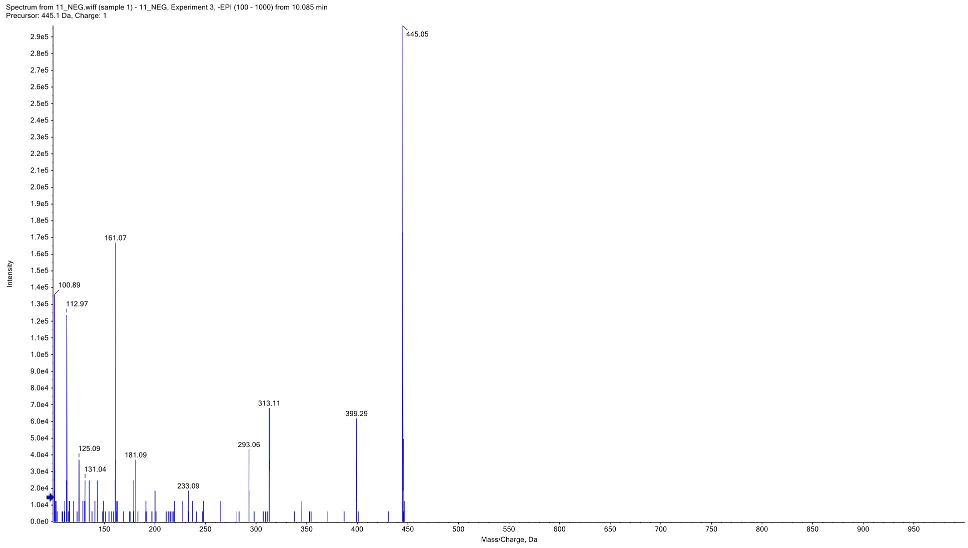

S11 Fig. Fragmentation patterns of Baicalein-7-O-glucuronide

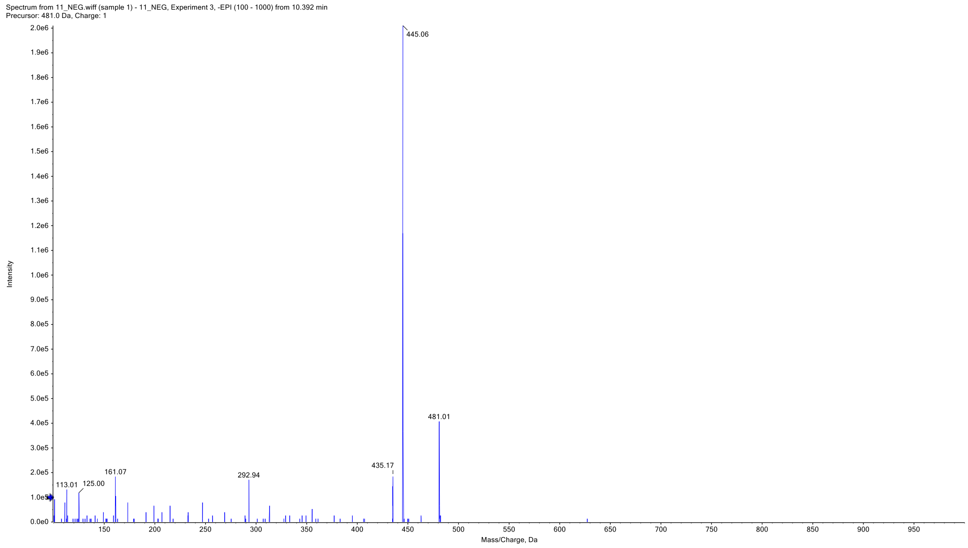

S12 Fig. Fragmentation patterns of Thymidine-5'-triphosphate sodium salt

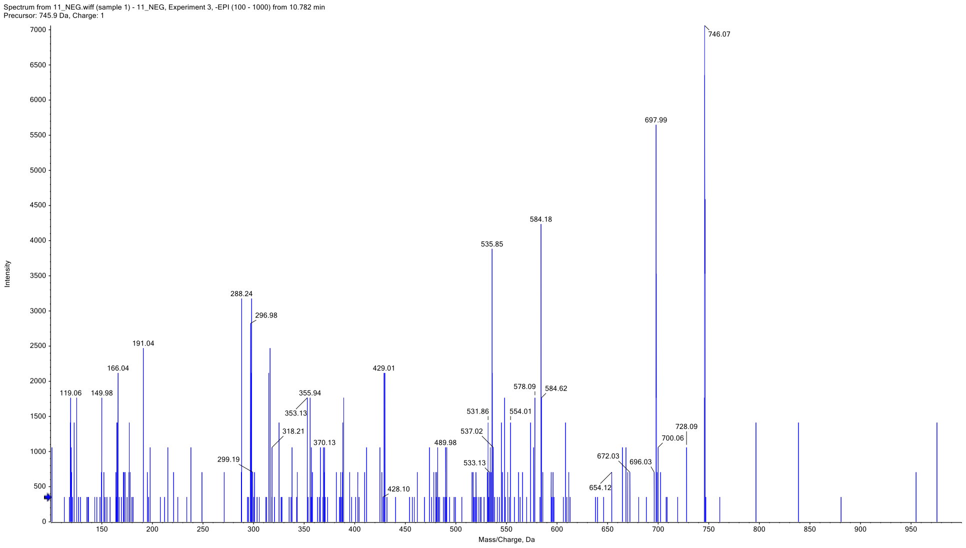

S13 Fig. Fragmentation patterns of NADP+

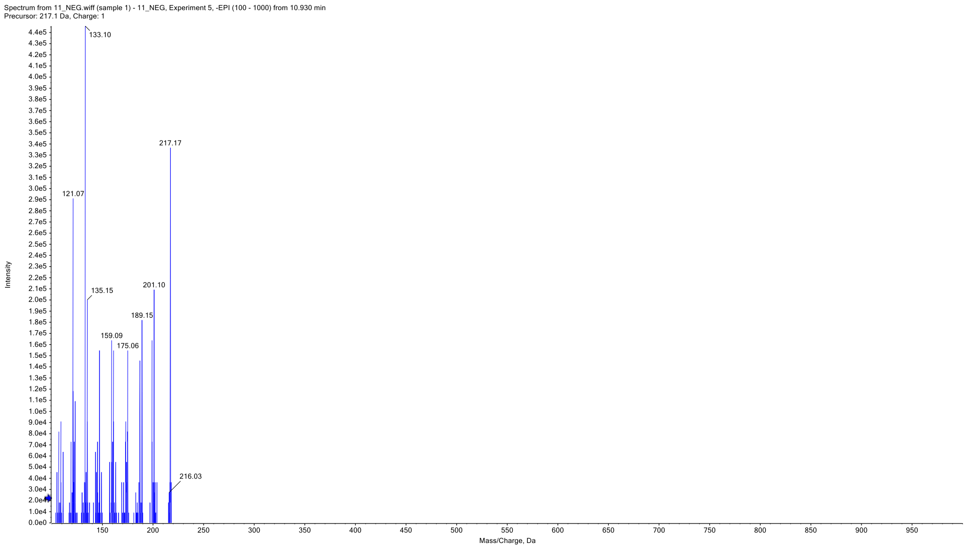

S14 Fig. Fragmentation patterns of L-beta-homotryptophan-HCl

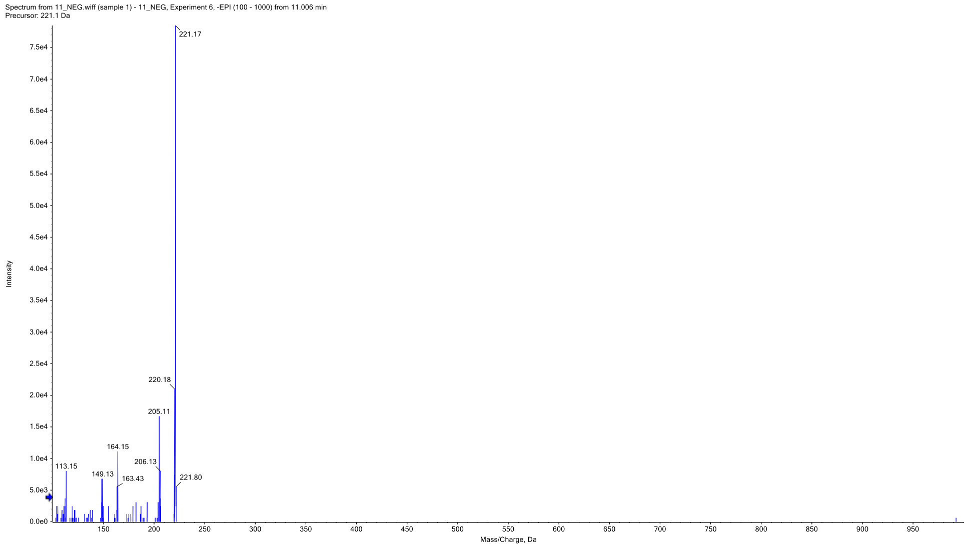

S15 Fig. Fragmentation patterns of Farnesol (mixture of isomers)

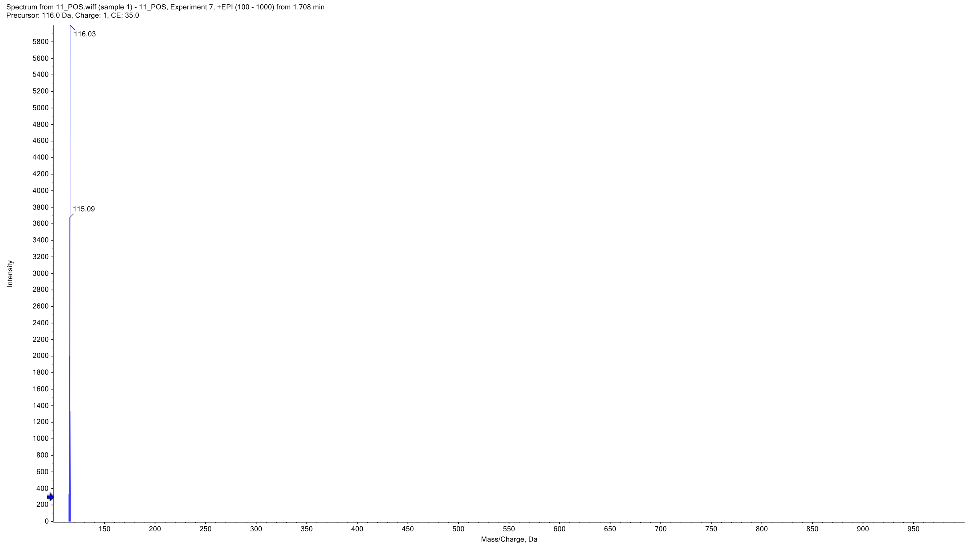

S16 Fig. Fragmentation patterns of L-PROLINE

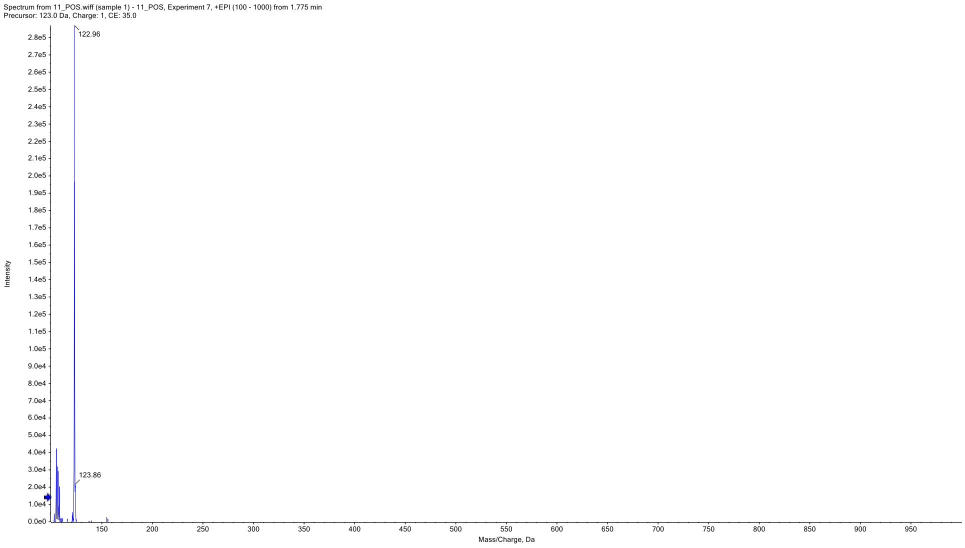

S17 Fig. Fragmentation patterns of NICOTINAMIDE

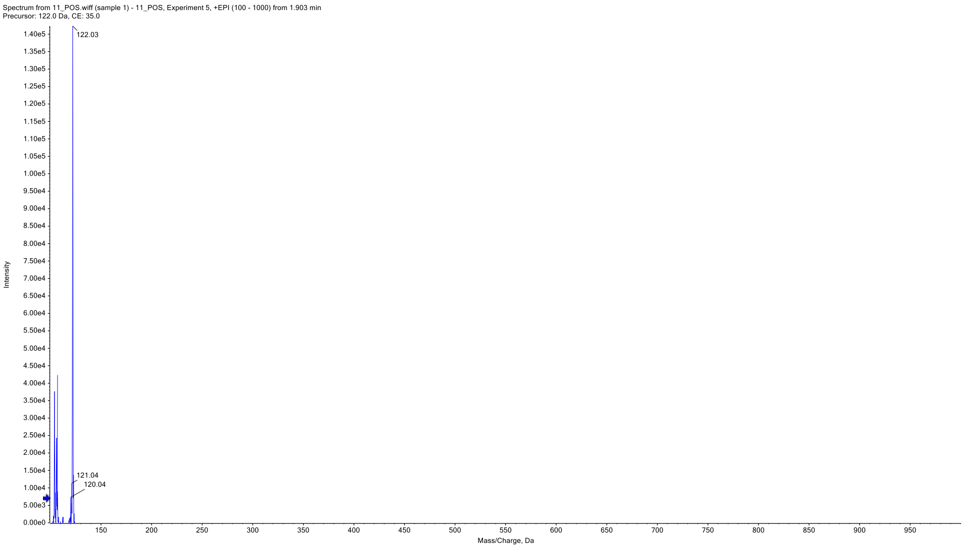

S18 Fig. Fragmentation patterns of N,N-DIMETHYLANILINE

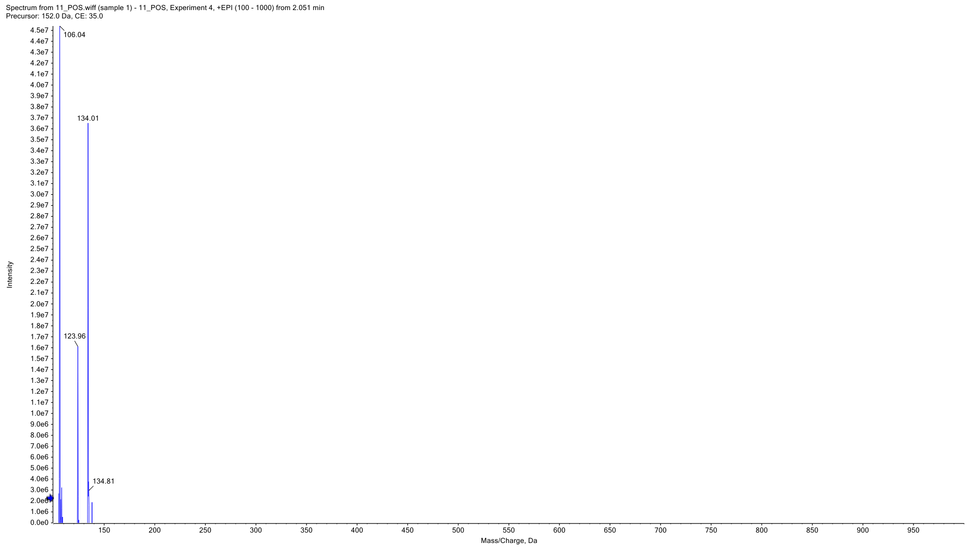

S19 Fig. Fragmentation patterns of Guanine

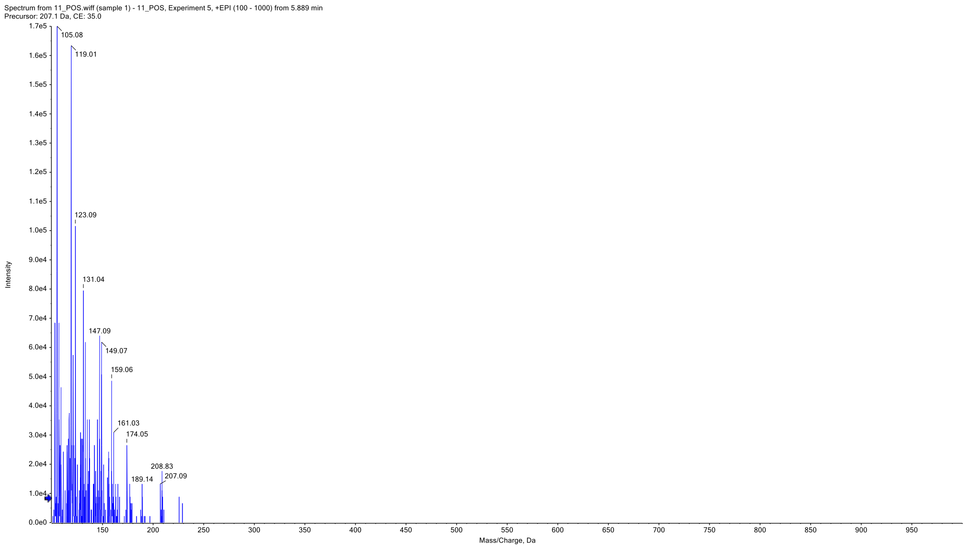

S20 Fig. Fragmentation patterns of 2-(4-ISOBUTYLPHENYL)PROPIONIC ACID

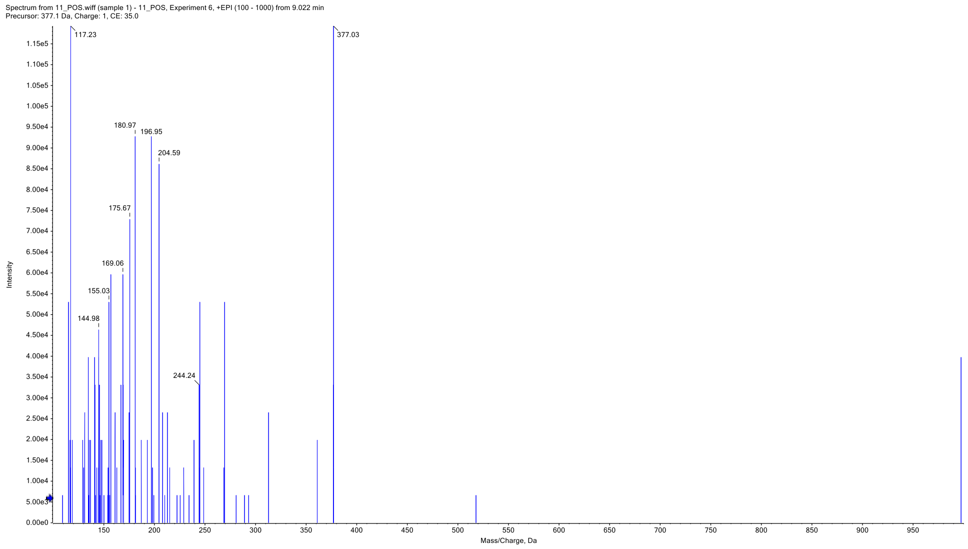

S21 Fig. Fragmentation patterns of (-)-RIBOFLAVIN

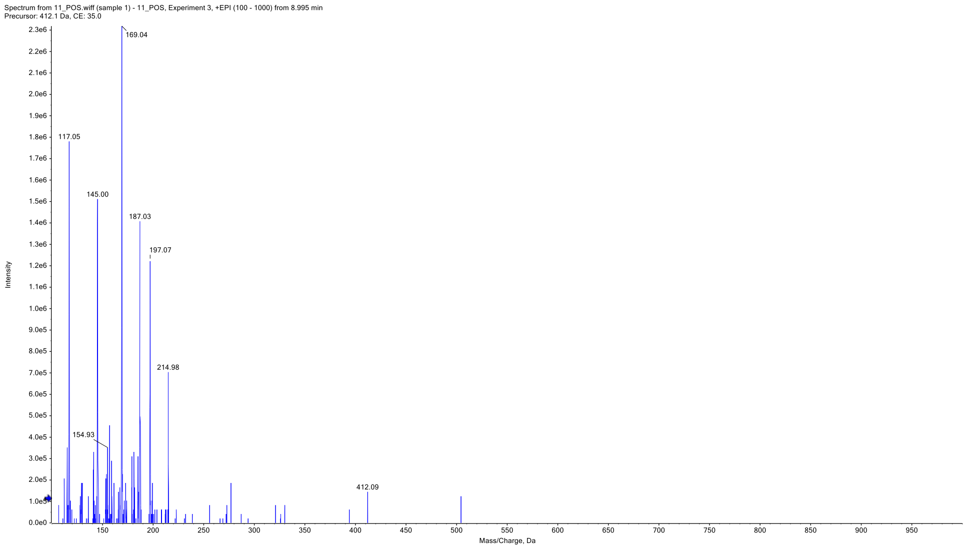

S22 Fig. Fragmentation patterns of 1-Decanoyl-2-Hydroxy-sn-Glycero-3-Phosphocholine

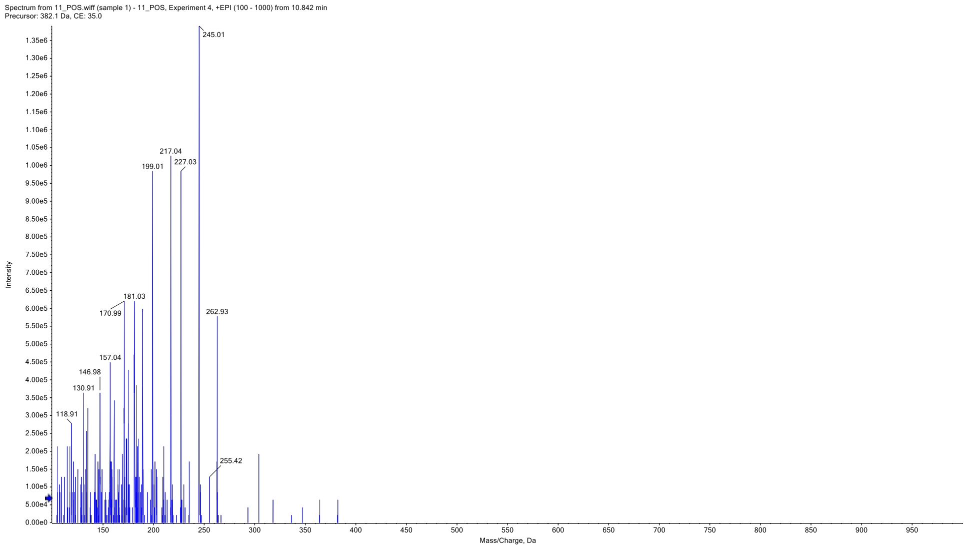

S23 Fig. Fragmentation patterns of trans-Zeatin-9-glucoside

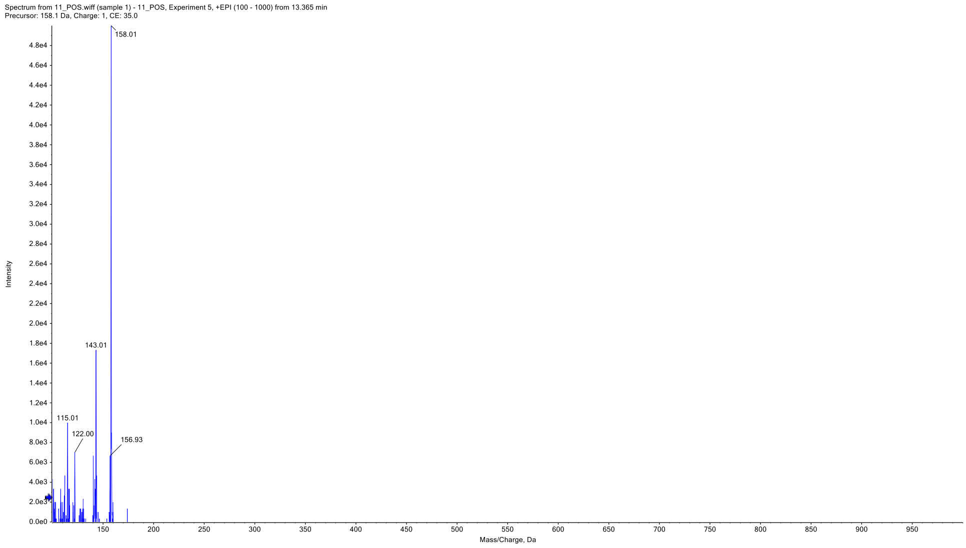

S24 Fig. Fragmentation patterns of N-Tigloylglycine

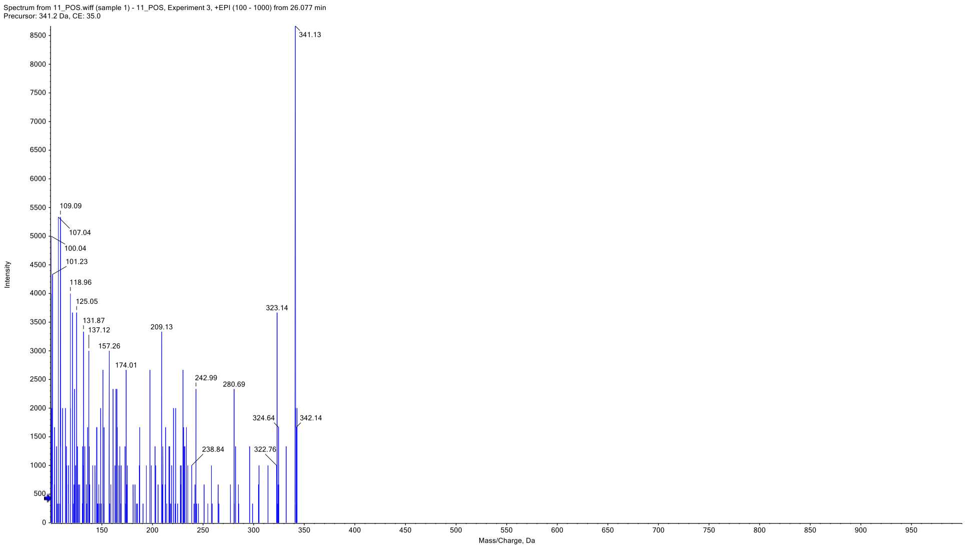

S25 Fig. Fragmentation patterns of alpha-D-Glucose-1,6-diphosphate

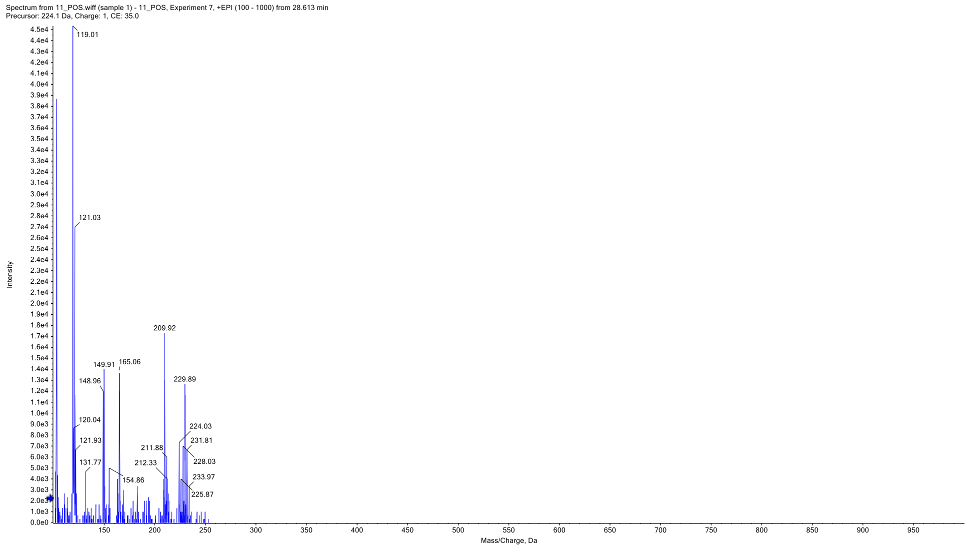

S26 Fig. Fragmentation patterns of Cerulenin

S1 Table. Cell viability of HeLa cells upon treatment for 48h using CCK8 assay (raw data)

	rep 1	Rep 2	rep 3	mean	SD
RTAq	6.87	18.99	11.72	1.53	4.98
Rapamycin	0.52	1.18	0.87	0.86	0.27
0.2% DMSO	77.38	91.42	90.01	86.27	6.31

S2 Table. KEGG pathways enrichment of the molecular mechanism of RTAq extract

Term	Genes	Fold Enrichment	FDR
hsa05200:Pathways in cancer	RET, ITGB1, GSK3B, CXCL8, HSP90AB1, IL23R, SLC2A1, PIK3CD, KEAP1, PIK3CB, FGF1, FGF2, IKBKB, EDNRA, CASP8, EDNRB, CASP3, AKT2, AKT3, AKT1, EP300, JAK2, PRKACA, JAK3, PDGFRB, PDGFRA, HSP90AA1, MAP2K2, CHUK, MMP1, MMP2, F2R, PRKCA, F2, MMP9, RHOA, TGFBR2, AR, PIK3CA, CCNE1, PPARG, RAF1, CAMK2D, FH, HDAC2, PTGER1, HDAC1, GSTP1, PTGER2, LPAR1, CXCR4, LPAR2, LPAR3, PIK3R1, PTGS2, HIF1A, EGFR, RXRB, MAPK9, MAPK8, RXRA, ABL1, MAPK1, HES1, NTRK1, EGLN1, NOS2, STAT1, STAT3, ESR1, MTOR, NFKB1, VEGFA, RAD51, CDK4, CDK2, GRB2, NFE2L2	2.940133	1.66E-16
hsa04931:Insulin resistance	PYGB, GSK3B, PRKAA1, SLC2A1, PIK3CD, PYGM, PIK3CB, PIK3R1, SLC2A4, PRKCZ, ACACB, IKBKB, MAPK9, RPS6KA3, GYS1, MAPK8, AKT2, AKT3, RPS6KA1, AKT1, CD36, PTPN1, PRKAB2, NOS3, PDPK1, PRKCD, GFPT1, STAT3, OGA, PTPN11, MTOR, NFKB1, PIK3CA, PPARA	6.266889	3.32E-16
hsa05215:Prostate cancer	GSK3B, HSP90AB1, GSTP1, PIK3CD, PLAT, PIK3CB, PIK3R1, EGFR, IKBKB, PLAU, AKT2, AKT3, AKT1, MAPK1, EP300, PDGFRB, PDGFRA, HSP90AA1, MAP2K2, CHUK, PDPK1, MMP3, MMP9, MTOR, NFKB1, AR, PIK3CA, CCNE1, CDK2, GRB2, RAF1	6.355288	5.82E-15
hsa05417:Lipid and atherosclerosis	GSK3B, CAMK2D, CXCL8, HSP90AB1, ITPR1, PIK3CD, PIK3CB, PIK3R1, AGER, IKBKB, RXRB, MAPK9, MAPK8, CASP8, CYP2B6, RXRA, TBK1, IRAK1, CASP6, CASP3, AKT2, AKT3, AKT1, MAPK1, CD36, JAK2, MAP3K5, MAP2K3, HSP90AA1, CHUK, MMP1, NOS3, PDPK1, MMP3, STAT3, PRKCA, MMP9, RHOA, NFKB1, CYP2A6, PIK3CA, PPARG, TLR4, NFE2L2, TLR2	4.185606	1.20E-14
hsa04066:HIF-1 signaling pathway	CAMK2D, FLT1, SERPINE1, SLC2A1, PIK3CD, PIK3CB, PIK3R1, HIF1A, HK2, EGFR, LDHB, LDHA, AKT2, AKT3, AKT1, MAPK1, EP300, EIF4E, EGLN1, MAP2K2, NOS2, NOS3, STAT3, PRKCA, MTOR, NFKB1, VEGFA, PIK3CA, ALDOA, TLR4	5.529608	8.51E-13
hsa04024:cAMP signaling pathway	GRIA1, GRIA2, CAMK2D, CHRM1, PTGER2, PDE3B, PIK3CD, ADRB1, ADRB2, PIK3CB, PIK3R1, SLC9A1, MAPK9, GRIN2A, MAPK8, EDNRA, AKT2, AKT3, ADORA1, PDE4B, AKT1, MAPK1, EP300, FFAR2, DRD2, PRKACA, GRIA3, GRIA4, GHSR, MAP2K2,	3.822607	1.09E-12

	F2R, NPY1R, HTR1B, SSTR2, RHOA, NFKB1, GRIN1, SSTR5, ADORA2A, PIK3CA, RAF1, PPARA, CFTR		
hsa05235:PD-L1 expression and PD-1 checkpoint pathway in cancer	PIK3CD, PIK3CB, PIK3R1, HIF1A, EGFR, IKBKB, AKT2, AKT3, AKT1, MAPK1, JAK2, MAP2K3, MAP2K2, CSNK2A1, CHUK, STAT1, CSNK2A2, STAT3, PTPN11, MTOR, NFKB1, PIK3CA, LCK, CSNK2B, RAF1, TLR4, TLR2	6.027273	1.65E-12
hsa04910:Insulin signaling pathway	PYGB, GSK3B, PRKAA1, PDE3B, PIK3CD, PYGM, PIK3CB, PIK3R1, SLC2A4, PRKCZ, ACACB, HK2, ACACA, IKBKB, MAPK9, GYS1, MAPK8, AKT2, AKT3, AKT1, MAPK1, PRKACA, EIF4E, PTPN1, PRKAB2, MAP2K2, PDPK1, MTOR, PIK3CA, FASN, GRB2, RAF1, FBP1	4.804348	1.74E-12
hsa05230:Central carbon metabolism in cancer	PDGFRB, NTRK1, RET, PDGFRA, MAP2K2, NTRK3, SLC2A1, PIK3CD, PIK3CB, PIK3R1, HIF1A, EGFR, MTOR, HK2, SIRT3, LDHB, SLC7A5, LDHA, PIK3CA, AKT2, AKT3, AKT1, MAPK1, RAF1	6.791293	2.41E-12
hsa04210:Apoptosis	ITPR1, PIK3CD, PIK3CB, PIK3R1, CTSS, IKBKB, MAPK9, MAPK8, CASP8, CASP6, CTSL, CASP3, AKT2, AKT3, LMNA, AKT1, MAPK1, CAPN1, CTSD, CTSB, MAP3K5, NTRK1, MAP2K2, PARP1, CHUK, PDPK1, NGF, TUBA4A, NFKB1, PIK3CA, RAF1, MAP3K14	4.727273	5.75E-12
hsa05161:Hepatitis B	CXCL8, PIK3CD, PIK3CB, PIK3R1, IKBKB, MAPK9, MAPK8, CASP8, TBK1, IRAK1, CASP3, AKT2, AKT3, PTK2B, AKT1, MAPK1, EP300, JAK2, JAK3, MAP2K3, MAP2K2, CHUK, STAT1, STAT3, PRKCA, MMP9, NFKB1, TGFBR2, PIK3CA, CCNE1, CDK2, GRB2, RAF1, TLR4, TLR2	4.313999	5.79E-12
hsa04722:Neurotrophin signaling pathway	GSK3B, CAMK2D, PIK3CD, PIK3CB, PIK3R1, IKBKB, MAPK9, RPS6KA3, MAPK8, IRAK1, AKT2, AKT3, RPS6KA1, ABL1, AKT1, MAPK1, MAP3K5, NTRK1, MAP2K2, PDPK1, NTRK3, PRKCD, PTPN11, NGF, RHOA, NFKB1, PIK3CA, MAPKAPK2, GRB2, RAF1	5.022727	5.79E-12
hsa04151:PI3K-Akt signaling pathway	RET, ITGB1, GSK3B, FLT1, CHRM1, HSP90AB1, ITGB3, PIK3CD, PIK3CB, FGF1, FGF2, PIK3CG, IKBKB, GYS1, AKT2, AKT3, KDR, AKT1, JAK2, JAK3, PDGFRB, PDGFRA, HSP90AA1, MAP2K2, CHUK, PDPK1, F2R, PRKCA, NGF, PIK3CA, CCNE1, RAF1, TLR4, TLR2, PRKAA1, LPAR1, LPAR2, LPAR3, PIK3R1, EGFR, RXRA, MAPK1, EIF4E, NTRK1, NOS3, MTOR, NFKB1, VEGFA, NR4A1, CDK4, CDK2, GRB2, PKN2, PKN1	2.996986	6.12E-12
hsa01521:EGFR tyrosine kinase inhibitor resistance	PDGFRB, PDGFRA, GSK3B, MAP2K2, STAT3, PIK3CD, PRKCA, PIK3CB, PIK3R1, FGF2, EGFR, MTOR, VEGFA, PIK3CA, AXL,	6.027273	2.73E-11

	AKT2, AKT3, KDR, AKT1, MAPK1, GRB2, JAK2, RAF1, EIF4E		
hsa05207:Chemical carcinogenesis - receptor activation	HSP90AB1, CACNA1B, PIK3CD, ADRB1, ADRB2, PIK3CB, PIK3R1, CYP3A4, FGF2, EGFR, RXRB, RPS6KA3, CYP2B6, RXRA, AKT2, AKT3, RPS6KA1, CYP1B1, AKT1, MAPK1, JAK2, PRKACA, CHRNB4, HSP90AA1, MAP2K2, VDR, EPHX1, STAT3, PRKCA, ESR1, MTOR, NFKB1, VEGFA, AR, KLF5, PIK3CA, GRB2, RAF1, PPARA	3.644397	4.08E-11
hsa04071:Sphingolipid signaling pathway	OPRD1, PIK3CD, PIK3CB, PIK3R1, PRKCZ, MAPK9, MAPK8, ADORA3, AKT2, AKT3, ADORA1, AKT1, MAPK1, FYN, S1PR2, S1PR5, CTSD, MAP3K5, ABCC1, MAP2K2, SPHK2, NOS3, PDPK1, SPHK1, PRKCA, RHOA, NFKB1, PIK3CA, RAF1	4.775708	4.38E-11
hsa04920:Adipocytokine signaling pathway	PRKAA1, PRKAB2, CHUK, STAT3, SLC2A1, PTPN11, SLC2A4, ACACB, MTOR, NFKB1, CAMKK2, RXRB, IKBKB, MAPK9, MAPK8, RXRA, AKT2, AKT3, AKT1, CD36, JAK2, PPARA	6.314286	8.30E-11
hsa04933:AGE-RAGE signaling pathway in diabetic complications	CXCL8, SERPINE1, PIK3CD, PIK3CB, PIK3R1, PRKCZ, AGER, MAPK9, MAPK8, CASP3, AKT2, AKT3, AKT1, MAPK1, JAK2, NOS3, STAT1, MMP2, PRKCD, STAT3, PRKCA, NFKB1, TGFBR2, VEGFA, PIK3CA, CDK4	5.171917	8.95E-11
hsa04015:Rap1 signaling pathway	ITGB1, FLT1, ITGB3, LPAR1, FPR1, LPAR2, PIK3CD, LPAR3, PIK3CB, PIK3R1, FGF1, FGF2, PRKCZ, EGFR, GRIN2A, CNR1, AKT2, AKT3, P2RY1, KDR, AKT1, MAPK1, DRD2, MAP2K3, PDGFRB, PDGFRA, MAP2K2, MAGI3, F2R, PRKCA, NGF, RHOA, GRIN1, VEGFA, ADORA2A, PIK3CA, ADORA2B, RAF1	3.601201	8.95E-11
hsa04020:Calcium signaling pathway	RET, CAMK2D, FLT1, CHRM1, PTGER1, CACNA1B, ITPR1, HTR2C, CXCR4, ADRB1, ADRB2, HTR2A, FGF1, GRPR, FGF2, EGFR, GRIN2A, EDNRA, EDNRB, KDR, PTK2B, CD38, PRKACA, NTSR1, PDGFRB, NTRK1, PDGFRA, NOS2, SPHK2, NOS3, SPHK1, NTRK3, F2R, TACR2, PRKCA, TACR1, NGF, GRIN1, VEGFA, P2RX7, ADORA2A, ADORA2B	3.322119	8.95E-11
hsa05205:Proteoglycans in cancer	ITGB1, CAMK2D, ITGB3, ITPR1, PIK3CD, PIK3CB, PIK3R1, FGF2, HIF1A, EGFR, SLC9A1, PLAU, CTSL, CASP3, AKT2, AKT3, KDR, AKT1, MAPK1, PRKACA, MAP2K2, PDPK1, MMP2, STAT3, PRKCA, PTPN11, MMP9, ESR1, RHOA, MTOR, VEGFA, PIK3CA, GRB2, HPSE, RAF1, TLR4, TLR2	3.643939	1.17E-10
hsa05163:Human cytomegalovirus infection	GSK3B, CXCL8, PTGER1, PTGER2, ITGB3, ITPR1, CXCR4, PIK3CD, PIK3CB, PIK3R1, PTGS2, EGFR, IKBKB, CASP8, TBK1, CASP3,	3.467015	1.36E-10

	AKT2, AKT3, PTK2B, AKT1, MAPK1, CCR5, PRKACA, CCR1, PDGFRA, MAP2K2, CHUK, STAT3, PRKCA, HLA-A, RHOA, MTOR, NFKB1, VEGFA, STING1, PIK3CA, CDK4, GRB2, RAF1		
hsa05167:Kaposi sarcoma-associated herpesvirus infection	GSK3B, CXCL8, ITPR1, PIK3CD, PIK3CB, PIK3R1, PTGS2, FGF2, HIF1A, PIK3CG, IKBKB, MAPK9, MAPK8, CASP8, TBK1, CASP3, AKT2, AKT3, AKT1, MAPK1, EP300, JAK2, CCR5, CCR1, MAP2K2, CHUK, STAT1, STAT3, HLA-A, MTOR, NFKB1, VEGFA, PIK3CA, CDK4, MAPKAPK2, RAF1	3.690167	1.46E-10
hsa04072:Phospholipase D signaling pathway	CXCL8, LPAR1, LPAR2, PIK3CD, LPAR3, PIK3CB, PIK3R1, EGFR, PIK3CG, GRM4, GRM6, AKT2, AKT3, PTK2B, AKT1, MAPK1, FYN, PDGFRB, PDGFRA, MAP2K2, SPHK2, SPHK1, F2R, PRKCA, PTPN11, F2, RHOA, MTOR, PIK3CA, GRB2, RAF1	4.179988	1.65E-10
hsa04919:Thyroid hormone signaling pathway	GSK3B, HDAC2, HDAC3, THRA, HDAC1, ITGB3, SLC2A1, PIK3CD, PIK3CB, PIK3R1, HIF1A, SLC9A1, RXRB, RXRA, AKT2, AKT3, AKT1, MAPK1, EP300, PRKACA, MAP2K2, PDPK1, STAT1, PRKCA, ESR1, MTOR, PIK3CA, RAF1	4.611028	1.65E-10
hsa04935:Growth hormone synthesis, secretion and action	GSK3B, ITPR1, PIK3CD, PIK3CB, PIK3R1, GHRHR, MAPK9, MAPK8, AKT2, AKT3, AKT1, MAPK1, EP300, JAK2, PRKACA, MAP2K3, GHSR, MAP2K2, STAT1, STAT3, PRKCA, SSTR2, SSTR3, MTOR, SSTR5, PIK3CA, GRB2, RAF1	4.611028	1.65E-10
hsa05418:Fluid shear stress and atherosclerosis	PRKAA1, HSP90AB1, GSTP1, ITGB3, KEAP1, PIK3CD, PLAT, PIK3CB, PIK3R1, PRKCZ, IKBKB, MAPK9, MAPK8, CTSL, AKT2, AKT3, KDR, AKT1, MAP3K5, HSP90AA1, CHUK, NOS3, MMP2, MMP9, RHOA, NFKB1, VEGFA, PIK3CA, BMPR1A, NFE2L2	4.274662	1.98E-10
hsa04062:Chemokine signaling pathway	GSK3B, ITK, GSK3A, CXCL8, CXCR4, PIK3CD, CXCR6, PIK3CB, PIK3R1, PRKCZ, PIK3CG, IKBKB, GRK2, GRK5, AKT2, AKT3, PTK2B, AKT1, MAPK1, JAK2, CCR5, PRKACA, JAK3, CCR2, CCR1, CHUK, STAT1, PRKCD, STAT3, RHOA, NFKB1, FGR, PIK3CA, GRB2, RAF1	3.643429	3.48E-10
hsa05212:Pancreatic cancer	CHUK, STAT1, STAT3, PIK3CD, PIK3CB, PIK3R1, EGFR, MTOR, NFKB1, TGFBR2, VEGFA, IKBKB, MAPK9, MAPK8, RAD51, PIK3CA, CDK4, AKT2, AKT3, AKT1, MAPK1, RAF1	5.74026	3.74E-10
hsa04068:FoxO signaling pathway	PRKAA1, PIK3CD, PIK3CB, PIK3R1, SLC2A4, EGFR, IKBKB, MAPK9, MAPK8, AKT2, AKT3, AKT1, MAPK1, EP300, PRKAB2, MAP2K2, CHUK, PRMT1, PDPK1, PLK1, STAT3, CSNK1E, SIRT1, TGFBR2, PIK3CA, CDK2, GRB2, RAF1	4.261708	9.81E-10

hsa05135:Yersinia infection	ITGB1, GSK3B, CXCL8, PIK3CD, PIK3CB, PIK3R1, IKBKB, MAPK9, RPS6KA3, MAPK8, TBK1, IRAK1, AKT2, AKT3, RPS6KA1, PTK2B, AKT1, MAPK1, MAP2K3, MAP2K2, CHUK, RHOA, NFKB1, PIK3CA, LCK, PKN2, PKN1, TLR4	4.076416	2.73E-09
hsa05162:Measles	GSK3B, PIK3CD, PIK3CB, PIK3R1, IKBKB, MAPK9, MAPK8, CASP8, TBK1, IRAK1, CASP3, AKT2, AKT3, AKT1, JAK3, CSNK2A1, CHUK, STAT1, CSNK2A2, STAT3, NFKB1, PIK3CA, CCNE1, CDK4, CSNK2B, CDK2, TLR4, TLR2	4.04709	3.14E-09
hsa04010:MAPK signaling pathway	RET, FLT1, CACNA1B, FGF1, FGF2, EGFR, IKBKB, MAPK9, RPS6KA3, MAPK8, IRAK1, CASP3, AKT2, AKT3, RPS6KA1, KDR, AKT1, MAPK1, PRKACA, MAP4K4, MAP3K5, MAP2K3, PDGFRB, NTRK1, PDGFRA, MAP2K2, DUSP3, CHUK, PRKCA, NGF, NFKB1, TGFBR2, CDC25B, VEGFA, NR4A1, PPM1A, TAOK1, MAPKAPK2, GRB2, MAPT, RAF1, MAP3K14, MAP3K11	2.879697	3.16E-09
hsa04926:Relaxin signaling pathway	PIK3CD, PIK3CB, PIK3R1, PRKCZ, RXFP1, EGFR, MAPK9, MAPK8, EDNRB, AKT2, AKT3, AKT1, MAPK1, PRKACA, MAP2K2, NOS2, MMP1, NOS3, MMP2, PRKCA, MMP9, NFKB1, TGFBR2, VEGFA, PIK3CA, GRB2, RAF1	4.172727	3.17E-09
hsa04917:Prolactin signaling pathway	GSK3B, MAP2K2, STAT1, STAT3, PIK3CD, PIK3CB, PIK3R1, ESR1, NFKB1, MAPK9, MAPK8, PIK3CA, TH, AKT2, AKT3, AKT1, MAPK1, GRB2, JAK2, RAF1	5.659411	3.60E-09
hsa04660:T cell receptor signaling pathway	GSK3B, ITK, PIK3CD, PIK3CB, PIK3R1, IKBKB, MAPK9, MAPK8, AKT2, AKT3, AKT1, MAPK1, FYN, MAP2K2, CHUK, PDPK1, PTPN11, RHOA, NFKB1, TEC, PIK3CA, LCK, CDK4, GRB2, RAF1, MAP3K14	4.281669	3.75E-09
hsa05223:Non-small cell lung cancer	RET, MAP2K2, PDPK1, STAT3, PIK3CD, PRKCA, PIK3CB, PIK3R1, EGFR, RXRB, RXRA, PIK3CA, CDK4, AKT2, AKT3, AKT1, MAPK1, GRB2, RAF1, JAK3	5.504359	5.71E-09
hsa04936:Alcoholic liver disease	GSK3B, PRKAA1, CXCL8, ACACB, ACACA, CAMKK2, IKBKB, MAPK9, MAPK8, CASP8, TBK1, IRAK1, ALDH2, CASP3, AKT2, AKT3, AKT1, MAP3K5, MAP2K3, PRKAB2, CHUK, SIRT1, NFKB1, SCD, FASN, PPARA, TLR4, MAP3K14	3.906566	6.05E-09
hsa04370:VEGF signaling pathway	MAP2K2, NOS3, SPHK2, SPHK1, PIK3CD, PRKCA, PIK3CB, PIK3R1, PTGS2, VEGFA, PIK3CA, AKT2, MAPKAPK2, AKT3, KDR, AKT1, MAPK1, RAF1	6.027273	9.65E-09
hsa05220:Chronic myeloid leukemia	HDAC2, MAP2K2, CHUK, HDAC1, PIK3CD, PTPN11, PIK3CB, PIK3R1, NFKB1, TGFBR2,	5.218418	1.40E-08

	IKBKB, PIK3CA, CDK4, AKT2, AKT3, ABL1, AKT1, MAPK1, GRB2, RAF1		
hsa04152:AMPK signaling pathway	PRKAA1, PIK3CD, HMGCR, PIK3CB, PIK3R1, SLC2A4, ACACB, ACACA, CAMKK2, GYS1, AKT2, AKT3, AKT1, CD36, PRKAB2, PDPK1, SIRT1, MTOR, PIK3CA, SCD, FASN, ULK1, PPARG, FBP1, CFTR	4.11699	1.71E-08
hsa04625:C-type lectin receptor signaling pathway	CHUK, STAT1, PRKCD, ITPR1, PIK3CD, PTPN11, PIK3CB, PIK3R1, PTGS2, RHOA, NFKB1, IKBKB, MAPK9, MAPK8, CASP8, PIK3CA, AKT2, MAPKAPK2, AKT3, AKT1, MAPK1, RAF1, MAP3K14	4.400866	2.16E-08
hsa04620:Toll-like receptor signaling pathway	MAP2K3, MAP2K2, CXCL8, CHUK, STAT1, PIK3CD, PIK3CB, PIK3R1, NFKB1, IKBKB, MAPK9, MAPK8, CASP8, TBK1, IRAK1, PIK3CA, AKT2, AKT3, TLR8, AKT1, MAPK1, TLR4, TLR2	4.239366	4.38E-08
hsa05170:Human immunodeficiency virus 1 infection	ITPR1, CXCR4, PIK3CD, PIK3CB, PIK3R1, IKBKB, MAPK9, MAPK8, CASP8, TBK1, IRAK1, CASP3, AKT2, AKT3, PTK2B, AKT1, MAPK1, CCR5, MAP2K3, MAP2K2, CHUK, LIMK2, PRKCA, HLA-A, CDC25C, MTOR, NFKB1, STING1, PIK3CA, CDK1, RAF1, TLR4, TLR2	3.112676	4.98E-08
hsa04923:Regulation of lipolysis in adipocytes	PDE3B, NPY1R, PIK3CD, ADRB1, PIK3CB, PIK3R1, ADRB2, PTGS2, PTGS1, FABP4, PIK3CA, AKT2, AKT3, ADORA1, AKT1, PRKACA, MGLL	5.788906	4.98E-08
hsa05145:Toxoplasmosis	MAP2K3, ITGB1, NOS2, CHUK, PDPK1, STAT1, STAT3, PIK3CG, NFKB1, IKBKB, MAPK9, MAPK8, CASP8, IRAK1, CASP3, AKT2, AKT3, AKT1, MAPK1, JAK2, CCR5, TLR4, TLR2	4.162981	5.71E-08
hsa04914:Progesterone-mediated oocyte maturation	HSP90AA1, HSP90AB1, PLK1, PDE3B, PIK3CD, PIK3CB, PIK3R1, PKMYT1, CDC25C, CDC25B, MAPK9, RPS6KA3, MAPK8, PIK3CA, AKT2, AKT3, RPS6KA1, CDK2, CDK1, AKT1, MAPK1, RAF1, PRKACA	4.162981	5.71E-08
hsa04014:Ras signaling pathway	FLT1, PIK3CD, PIK3CB, PIK3R1, FGF1, FGF2, EGFR, IKBKB, MAPK9, GRIN2A, MAPK8, TBK1, AKT2, AKT3, KDR, ABL1, AKT1, MAPK1, PRKACA, PDGFRB, NTRK1, PDGFRA, MAP2K2, CHUK, PLA2G2A, PRKCA, PTPN11, NGF, RHOA, NFKB1, GRIN1, VEGFA, PIK3CA, GRB2, RAF1	2.954545	5.92E-08
hsa05142:Chagas disease	ACE, CXCL8, NOS2, CHUK, SERPINE1, PIK3CD, PIK3CB, PIK3R1, NFKB1, TGFBR2, IKBKB, MAPK9, MAPK8, CASP8, IRAK1, PIK3CA, AKT2, AKT3, AKT1, MAPK1, TLR4, TLR2	4.291262	6.87E-08
hsa04664:Fc epsilon RI signaling pathway	MAP2K3, MAP2K2, PDPK1, PIK3CD, PRKCA, PIK3CB, PIK3R1, MAPK9, MAPK8,	5.241107	7.70E-08

	PIK3CA, AKT2, AKT3, BTK, AKT1, MAPK1, GRB2, FYN, RAF1		
hsa05211:Renal cell carcinoma	EGLN1, FH, MAP2K2, SLC2A1, PIK3CD, PTPN11, PIK3CB, PIK3R1, HIF1A, VEGFA, PIK3CA, AKT2, AKT3, AKT1, EP300, MAPK1, GRB2, RAF1	5.166234	9.53E-08
hsa04611:Platelet activation	P2RY12, ITGB1, NOS3, ITGB3, F2R, ITPR1, PIK3CD, PIK3CB, PIK3R1, F2, PRKCZ, RHOA, PIK3CG, PTGS1, PIK3CA, AKT2, TBXAS1, P2RY1, AKT3, BTK, AKT1, MAPK1, FYN, PRKACA	3.857455	1.07E-07
hsa04922:Glucagon signaling pathway	PYGB, CAMK2D, PRKAA1, PRKAB2, PRMT1, PDE3B, SLC2A1, ITPR1, PYGM, ACACB, SIRT1, ACACA, LDHB, GYS1, LDHA, AKT2, AKT3, AKT1, EP300, PPARA, PRKACA, FBP1	4.130841	1.28E-07
hsa05231:Choline metabolism in cancer	PDGFRB, PDGFRA, MAP2K2, CHKA, PDPK1, PIK3CD, PRKCA, PIK3CB, PIK3R1, HIF1A, EGFR, MTOR, MAPK9, MAPK8, PIK3CA, AKT2, AKT3, AKT1, MAPK1, GRB2, RAF1	4.261708	1.59E-07
hsa05208:Chemical carcinogenesis - reactive oxygen species	KEAP1, PIK3CD, PIK3CB, PIK3R1, HIF1A, EGFR, IKBKB, MAPK9, MAPK8, AKT2, AKT3, ABL1, CYP1B1, AKT1, MAPK1, MAP3K5, PTPN1, MAP2K2, CHUK, PDPK1, EPHX1, PRKCD, AKR1C3, AKR1C2, PTPN11, AKR1C4, NFKB1, VEGFA, PIK3CA, GRB2, RAF1, MAP3K14, NFE2L2	2.933628	1.69E-07
hsa04613:Neutrophil extracellular trap formation	HDAC4, HDAC2, HDAC3, HDAC10, HDAC1, ITGB3, HDAC11, FPR1, PIK3CD, PIK3CB, PIK3R1, FPR2, HDAC8, HDAC9, AGER, HDAC7, AKT2, AKT3, AKT1, MAPK1, CTSG, MAP2K2, PRKCA, MTOR, NFKB1, PIK3CA, TLR8, RAF1, TLR4, TLR2	3.139205	1.69E-07
hsa05415:Diabetic cardiomyopathy	GSK3B, CAMK2D, SLC2A1, PIK3CD, PIK3CB, PIK3R1, SLC2A4, PRKCZ, AGER, MAPK9, GYS1, MAPK8, AKT2, AKT3, AKT1, CD36, CTSD, ACE, PARP1, NOS3, MMP2, PRKCD, GFPT1, GSR, PRKCA, MMP9, MTOR, NFKB1, TGFBR2, PIK3CA, PPARA	3.038137	1.99E-07
hsa05203:Viral carcinogenesis	HDAC4, HDAC2, HDAC3, HDAC10, HDAC1, HDAC11, PIK3CD, PIK3CB, PIK3R1, HDAC8, HDAC9, HDAC7, POLB, CASP8, CASP3, MAPK1, EP300, CCR5, PRKACA, JAK3, STAT3, HLA-A, RHOA, NFKB1, PIK3CA, CCNE1, CDK4, MAPKAPK2, CDK2, CDK1, GRB2	3.038137	1.99E-07
hsa05214:Glioma	PDGFRB, PDGFRA, CAMK2D, MAP2K2, PIK3CD, PRKCA, PIK3CB, PIK3R1, EGFR, MTOR, PIK3CA, CDK4, AKT2, AKT3, AKT1, MAPK1, GRB2, RAF1	4.758373	3.04E-07
hsa04012:ErbB signaling pathway	GSK3B, CAMK2D, MAP2K2, PIK3CD, PRKCA, PIK3CB, PIK3R1, EGFR, MTOR, MAPK9, MAPK8, PIK3CA, AKT2, AKT3, ABL1, AKT1, MAPK1, GRB2, RAF1	4.438689	3.75E-07

hsa05031:Amphetamine addiction	GRIA1, GRIA2, CAMK2D, HDAC2, MAOB, MAOA, HDAC1, PRKCA, PDYN, SIRT1, SLC6A3, GRIN1, GRIN2A, TH, PRKACA, GRIA3, GRIA4	4.949934	4.07E-07
hsa05210:Colorectal cancer	GSK3B, MAP2K2, PIK3CD, PIK3CB, PIK3R1, RHOA, EGFR, MTOR, TGFBR2, MAPK9, MAPK8, PIK3CA, CASP3, AKT2, AKT3, AKT1, MAPK1, GRB2, RAF1	4.38767	4.38E-07
hsa04218:Cellular senescence	CXCL8, SERPINE1, ITPR1, PIK3CD, PIK3CB, PIK3R1, CHEK2, AKT2, AKT3, AKT1, MAPK1, CAPN1, MAP2K3, MAP2K2, HLA-A, SIRT1, MTOR, NFKB1, TGFBR2, PIK3CA, CCNE1, CDK4, MAPKAPK2, CDK2, CDK1, RAF1	3.327157	4.38E-07
hsa05169:Epstein-Barr virus infection	HDAC2, HDAC1, PIK3CD, PIK3CB, PIK3R1, IKBKB, MAPK9, MAPK8, CASP8, TBK1, IRAK1, CASP3, AKT2, AKT3, AKT1, HES1, JAK3, MAP2K3, CHUK, STAT1, STAT3, HLA-A, NFKB1, PIK3CA, CCNE1, CDK4, CDK2, BTK, MAP3K14, TLR2	2.9691	5.08E-07
hsa05010:Alzheimer disease	APP, GSK3B, CHRM1, ITPR1, PIK3CD, PIK3CB, PIK3R1, PTGS2, HSD17B10, AGER, IKBKB, APH1A, MAPK9, GRIN2A, MAPK8, CASP8, PSMB5, PSMB2, CASP3, AKT2, TUBB1, AKT3, PSMB1, AKT1, MAPK1, CAPN1, MAP3K5, MAP2K2, MME, NOS2, CSNK2A1, CHUK, CSNK2A2, ADAM10, CSNK1E, MTOR, TUBA4A, NFKB1, GRIN1, PIK3CA, CDK5, CSNK2B, ULK1, MAPT, RAF1	2.312253	5.17E-07
hsa04930:Type II diabetes mellitus	PRKCD, CACNA1B, PIK3CD, PIK3CB, PIK3R1, SLC2A4, PRKCZ, MTOR, HK2, IKBKB, MAPK9, MAPK8, PIK3CA, MAPK1	5.984526	6.02E-07
hsa01100:Metabolic pathways	CDA, AMY2A, ABAT, GBA1, GBA2, PNP, TDO2, ANPEP, NAMPT, ENPP2, GSTK1, ST6GAL1, MIF, PGD, FOLH1, LAP3, HPRT1, PNLIP, IDO1, PTGES, DNMT1, MAOB, MAOA, ALOX12, ACACB, CYP19A1, ACACA, LDHB, LDHA, CYP2B6, AMDHD2, LTA4H, ST3GAL1, CHKA, GFPT1, TYR, ALOX15B, DHFR, QDPR, SQLE, BHMT, GCLC, AKR1B10, TH, P4HA1, CYP11B1, FASN, PAH, TBXAS1, HPSE, ADA, EZH2, GPI, PYGB, HEXB, HEXA, PDE3B, ADK, PIK3CD, AKR1B1, PYGM, PIK3CB, HK2, PIK3CG, GYS1, ALDH2, PDE4B, CD38, TK1, GUSB, TPI1, SPHK2, SPHK1, GAA, CAD, AMPD1, SIRT1, LSS, SIRT2, SIRT3, CYP2A6, PIK3CA, ALDH1A1, PDE5A, MVD, UMPS, ALDOA, FBP1, MGLL, BCAT2, DTYMK, FH, AHCY, GSTP1, HSD17B3, HMGCR, CYP3A4, PTGS2, TYMS, HSD17B10, TYMP, PTGS1, NT5E, PDE11A, XDH, AOC3, DUT, NOS2, NOS3, AGL, GSR, PLA2G2A, AKR1C3,	1.544465	6.23E-07

	AKR1C2, AKR1C4, DHODH, GLB1, SCD, PDE7A, GLA		
hsa01522:Endocrine resistance	MAP2K2, MMP2, PIK3CD, PIK3CB, PIK3R1, ESR1, MMP9, EGFR, MTOR, MAPK9, MAPK8, PIK3CA, CDK4, AKT2, AKT3, AKT1, MAPK1, GRB2, RAF1, PRKACA	4.05877	6.37E-07
hsa04668:TNF signaling pathway	MAP2K3, CHUK, MMP3, PIK3CD, PIK3CB, PIK3R1, PTGS2, MMP9, NFKB1, IKBKB, MAPK9, MMP14, MAPK8, CASP8, PIK3CA, CASP3, AKT2, AKT3, AKT1, MAPK1, MAP3K14, MAP3K5	3.714286	6.64E-07
hsa04380:Osteoclast differentiation	CHUK, STAT1, ITGB3, ITPR1, PIK3CD, PIK3CB, PIK3R1, NFKB1, TGFBR2, IKBKB, MAPK9, MAPK8, TEC, PIK3CA, LCK, AKT2, AKT3, BTK, AKT1, MAPK1, GRB2, FYN, PPARG, MAP3K14	3.395647	8.96E-07
hsa05222:Small cell lung cancer	ITGB1, NOS2, CHUK, PIK3CD, PIK3CB, PIK3R1, PTGS2, NFKB1, RXRB, IKBKB, RXRA, PIK3CA, CCNE1, CDK4, CASP3, AKT2, AKT3, CDK2, AKT1	4.104594	1.10E-06
hsa05206:MicroRNAs in cancer	HDAC4, DNMT1, HDAC2, HDAC1, ITGB3, PIK3CD, PIK3CB, PIK3R1, PTGS2, EGFR, IKBKB, PLAU, CASP3, ABL1, CYP1B1, MAPK1, EP300, PDGFRB, PDGFRA, ABCC1, ST14, MAP2K2, STAT3, PRKCA, CDC25C, MMP9, SIRT1, RHOA, MTOR, NFKB1, CDC25B, VEGFA, PIK3CA, CCNE1, GRB2, MDM4, RAF1, EZH2	2.44697	1.25E-06
hsa04140:Autophagy - animal	PRKAA1, ITPR1, PIK3CD, PIK3CB, PIK3R1, HIF1A, CAMKK2, MAPK9, MAPK8, TBK1, CTSL, AKT2, AKT3, AKT1, MAPK1, PRKACA, CTSD, CTSB, MAP2K2, PDPK1, PRKCD, MTOR, PIK3CA, ATG4B, ULK1, RAF1	3.090909	1.59E-06
hsa05221:Acute myeloid leukemia	MAP2K2, CHUK, STAT3, PIK3CD, PIK3CB, PIK3R1, MTOR, NFKB1, IKBKB, PIK3CA, AKT2, AKT3, AKT1, MAPK1, GRB2, RAF1	4.727273	1.63E-06
hsa05160:Hepatitis C	GSK3B, PIK3CD, PIK3CB, PIK3R1, EGFR, IKBKB, CASP8, RXRA, TBK1, CASP3, AKT2, AKT3, AKT1, MAPK1, MAP2K2, CHUK, STAT1, STAT3, NFKB1, PIK3CA, CDK4, CDK2, GRB2, RAF1, PPARA	3.158948	1.78E-06
hsa04915:Estrogen signaling pathway	HSP90AA1, MAP2K2, HSP90AB1, NOS3, MMP2, PRKCD, ITPR1, PIK3CD, PIK3CB, PIK3R1, OPRM1, ESR1, MMP9, EGFR, PIK3CA, AKT2, AKT3, AKT1, MAPK1, GRB2, RAF1, PRKACA, CTSD	3.324395	2.21E-06
hsa04750:Inflammatory mediator regulation of TRP channels	MAP2K3, NTRK1, CAMK2D, PTGER2, PRKCD, ITPR1, PIK3CD, HTR2C, PRKCA, ALOX12, PIK3CB, PIK3R1, HTR2A, NGF, MAPK9, MAPK8, PIK3CA, P2RY2, PRKACA	3.855831	2.66E-06
hsa04728:Dopaminergic synapse	GRIA1, GRIA2, GSK3B, CAMK2D, GSK3A, MAOB, MAOA, CACNA1B, ITPR1, PRKCA, SLC6A3, MAPK9, GRIN2A, MAPK8, TH,	3.348485	3.46E-06

	AKT2, AKT3, AKT1, DRD2, PRKACA, GRIA3, GRIA4		
hsa05017:Spinocerebellar ataxia	GRIA1, GRIA2, ITPR1, PIK3CD, PRKCA, PIK3CB, PIK3R1, PDYN, MTOR, GRIN1, MAPK9, GRIN2A, MAPK8, PSMB5, PIK3CA, PSMB2, AKT2, AKT3, PSMB1, AKT1, ULK1, GRIA3, MAP3K5	3.208965	3.92E-06
hsa05218:Melanoma	PDGFRB, PDGFRA, MAP2K2, PIK3CD, PIK3CB, PIK3R1, FGF1, FGF2, EGFR, PIK3CA, CDK4, AKT2, AKT3, AKT1, MAPK1, RAF1	4.403487	3.93E-06
hsa04540:Gap junction	PDGFRB, PDGFRA, MAP2K2, LPAR1, ITPR1, HTR2C, PRKCA, ADRB1, HTR2A, EGFR, TUBA4A, TUBB1, CDK1, MAPK1, GRB2, RAF1, DRD2, PRKACA	3.93083	3.93E-06
hsa04150:mTOR signaling pathway	GSK3B, PRKAA1, MAP2K2, CHUK, PDPK1, PIK3CD, PRKCA, PIK3CB, PIK3R1, RHOA, MTOR, IKBKB, SLC7A5, RPS6KA3, PIK3CA, AKT2, AKT3, RPS6KA1, AKT1, MAPK1, GRB2, ULK1, RAF1, EIF4E	3.051784	5.21E-06
hsa05213:Endometrial cancer	GSK3B, MAP2K2, PDPK1, PIK3CD, PIK3CB, PIK3R1, EGFR, PIK3CA, AKT2, AKT3, AKT1, MAPK1, GRB2, RAF1	4.767334	7.99E-06
hsa04371:Apelin signaling pathway	HDAC4, PRKAA1, PRKAB2, MAP2K2, NOS2, NOS3, SPHK2, SPHK1, PDE3B, SERPINE1, ITPR1, PLAT, MTOR, PIK3CG, SLC9A1, AKT2, AKT3, APLNR, AKT1, MAPK1, RAF1, PRKACA	3.157143	8.46E-06
hsa04659:Th17 cell differentiation	HSP90AA1, HSP90AB1, CHUK, STAT1, IL23R, STAT3, HIF1A, MTOR, NFKB1, TGFBR2, RXRB, IKBKB, MAPK9, MAPK8, RXRA, LCK, MAPK1, JAK2, JAK3	3.534512	8.82E-06
hsa04211:Longevity regulating pathway	PRKAA1, PRKAB2, PIK3CD, PIK3CB, PIK3R1, SIRT1, MTOR, NFKB1, CAMKK2, PIK3CA, AKT2, AKT3, AKT1, ULK1, PPARG, PRKACA, EIF4E	3.794949	1.24E-05
hsa04148:Efferocytosis	P2RY12, CAMK2D, MAP2K2, SPHK2, PTGER2, ITGB3, SPHK1, SLC2A1, ADAM10, PTPN11, PTGS2, AGER, HIF1A, SIRT1, RXRA, AXL, CASP3, P2RY2, MAPKAPK2, MAPK1, PPARG, CD36, JAK2	2.962121	1.35E-05
hsa04213:Longevity regulating pathway - multiple species	PRKAA1, HDAC2, PRKAB2, HDAC1, PIK3CD, PIK3CB, PIK3R1, SIRT1, MTOR, PIK3CA, AKT2, AKT3, AKT1, PRKACA	4.536657	1.35E-05
hsa04614:Renin-angiotensin system	ACE2, ENPEP, ACE, MME, KLK1, ANPEP, PREP, CTSG, LNPEP	7.86166	1.41E-05
hsa04932:Non-alcoholic fatty liver disease	GSK3B, GSK3A, PRKAA1, PRKAB2, CXCL8, PIK3CD, PIK3CB, PIK3R1, NFKB1, IKBKB, MAPK9, MAPK8, CASP8, RXRA, PIK3CA, CASP3, AKT2, AKT3, AKT1, PPARG, PPARA, MAP3K11, MAP3K5	2.943254	1.46E-05
hsa05164:Influenza A	PRSS1, MAP2K2, CXCL8, CHUK, STAT1, PIK3CD, PRKCA, PIK3CB, PIK3R1, NFKB1, IKBKB, CASP8, TBK1, PIK3CA, CDK4,	2.803383	1.99E-05

	CASP3, AKT2, AKT3, AKT1, EP300, MAPK1, JAK2, RAF1, TLR4		
hsa04726:Serotonergic synapse	APP, MAOB, MAOA, CACNA1B, ITPR1, HTR1B, HTR2C, PRKCA, ALOX12, HTR3A, HTR2A, PTGS2, ALOX15B, SLC6A4, PTGS1, CASP3, MAPK1, RAF1, PRKACA	3.319368	1.99E-05
hsa04725:Cholinergic synapse	ACHE, CAMK2D, CHRNB4, CHRM1, CACNA1B, ITPR1, PIK3CD, PRKCA, PIK3CB, PIK3R1, PIK3CG, PIK3CA, AKT2, AKT3, AKT1, MAPK1, FYN, JAK2, PRKACA	3.319368	1.99E-05
hsa05166:Human T-cell leukemia virus 1 infection	SLC2A1, PIK3CD, PIK3CB, PIK3R1, IKBKB, POLB, MAPK9, MAPK8, CHEK2, AKT2, AKT3, AKT1, MAPK1, EP300, PRKACA, JAK3, MMP7, MAP2K2, CHUK, HLA-A, NFKB1, TGFBR2, PIK3CA, CCNE1, LCK, CDK4, CDK2, MAP3K14	2.522625	2.25E-05
hsa04657:IL-17 signaling pathway	GSK3B, HSP90AA1, CXCL8, HSP90AB1, CHUK, MMP1, MMP3, PTGS2, MMP9, NFKB1, IKBKB, MAPK9, MAPK8, CASP8, TBK1, CASP3, MAPK1	3.595215	2.30E-05
hsa05165:Human papillomavirus infection	ITGB1, GSK3B, HDAC2, HDAC1, ITGB3, PIK3CD, PIK3CB, PIK3R1, PTGS2, PRKCZ, EGFR, IKBKB, CASP8, TBK1, CASP3, AKT2, AKT3, AKT1, MAPK1, EP300, HES1, PRKACA, PDGFRB, MAP2K2, CHUK, STAT1, HLA-A, MTOR, NFKB1, VEGFA, PIK3CA, CCNE1, CDK4, CDK2, GRB2, RAF1	2.17199	2.89E-05
hsa04720:Long-term potentiation	GRIA1, GRIA2, CAMK2D, MAP2K2, ITPR1, PRKCA, GRIN1, RPS6KA3, GRIN2A, RPS6KA1, EP300, MAPK1, RAF1, PRKACA	4.1981	2.99E-05
hsa05030:Cocaine addiction	GRIA2, GRIN2A, MAOB, CDK5, TH, MAOA, PDYN, DRD2, PRKACA, SLC6A3, NFKB1, GRIN1	4.920223	3.05E-05
hsa05219:Bladder cancer	MAP2K2, CXCL8, MMP1, CDK4, MMP2, MAPK1, RAF1, MMP9, EGFR, TYMP, VEGFA	5.390244	3.31E-05
hsa05152:Tuberculosis	CAMK2D, NOS2, SPHK2, STAT1, VDR, SPHK1, RHOA, CTSS, NFKB1, MAPK9, MAPK8, CASP8, IRAK1, CASP3, AKT2, AKT3, AKT1, EP300, MAPK1, JAK2, RAF1, CTSD, TLR4, TLR2	2.678788	3.85E-05
hsa04662:B cell receptor signaling pathway	GSK3B, MAP2K2, CHUK, PIK3CD, PIK3CB, PIK3R1, NFKB1, IKBKB, PIK3CA, AKT2, AKT3, BTK, AKT1, MAPK1, GRB2, RAF1	3.532468	5.18E-05
hsa04912:GnRH signaling pathway	MAP2K3, CAMK2D, MAP2K2, MMP2, PRKCD, ITPR1, PRKCA, EGFR, MAPK9, MMP14, MAPK8, PTK2B, MAPK1, GRB2, RAF1, PRKACA	3.4565	6.61E-05
hsa05171:Coronavirus disease - COVID-19	CFD, CXCL8, PIK3CD, F13A1, PIK3CB, PIK3R1, EGFR, IKBKB, MAPK9, MAPK8, TBK1, IRAK1, MAPK1, ACE, CHUK, MMP1, STAT1, MMP3, STAT3, PRKCA, F2, NFKB1, ACE2, STING1, PIK3CA, TLR8, TLR4, TLR2	2.363636	6.61E-05
hsa05226:Gastric cancer	GSK3B, MAP2K2, PIK3CD, PIK3CB, PIK3R1, FGF1, FGF2, EGFR, MTOR, TGFBR2, RXRB,	2.812727	6.70E-05

	RXRA, PIK3CA, CCNE1, AKT2, AKT3, CDK2, AKT1, MAPK1, GRB2, RAF1		
hsa05034:Alcoholism	HDAC4, HDAC2, HDAC3, MAOB, HDAC10, MAOA, HDAC1, HDAC11, PDYN, HDAC8, HDAC9, SLC6A3, HDAC7, CAMKK2, GRIN1, GRIN2A, ADORA2A, TH, ADORA2B, MAPK1, GRB2, RAF1, DRD2, PRKACA	2.564797	7.35E-05
hsa05022:Pathways of neurodegeneration - multiple diseases	GRIA1, GRIA2, APP, GSK3B, CAMK2D, VCP, CHRM1, CACNA1B, ITPR1, PTGS2, HSD17B10, AGER, SLC6A3, MAPK9, GRIN2A, MAPK8, CASP8, TBK1, PSMB5, PSMB2, CASP3, TUBB1, PSMB1, MAPK1, CAPN1, GRIA3, GRIA4, MAP3K5, MAP2K3, MAP2K2, NOS2, CSNK2A1, CSNK2A2, PRKCA, CSNK1E, PDYN, MTOR, TUBA4A, NFKB1, GRIN1, CDK5, CSNK2B, ULK1, MAPT, RAF1	1.871824	8.11E-05
hsa04510:Focal adhesion	ITGB1, GSK3B, FLT1, ITGB3, PIK3CD, PIK3CB, PIK3R1, EGFR, MAPK9, MAPK8, AKT2, AKT3, KDR, AKT1, MAPK1, FYN, PDGFRB, PDGFRA, PDPK1, PRKCA, RHOA, VEGFA, PIK3CA, GRB2, RAF1	2.47425	8.68E-05
hsa01524:Platinum drug resistance	TOP2A, PDPK1, GSTP1, PIK3CD, PIK3CB, PIK3R1, CASP8, PIK3CA, CASP3, AKT2, AKT3, AKT1, MAPK1, MAP3K5	3.750303	9.32E-05
hsa01523:Antifolate resistance	DHFR, IKBKB, ABCC1, CHUK, GGH, ALOX12, TYMS, NFKB1, ABCG2	6.027273	1.01E-04
hsa05132:Salmonella infection	CXCL8, HSP90AB1, PIK3CD, PIK3CB, PIK3CG, IKBKB, MAPK9, MAPK8, CASP8, IRAK1, CASP3, AKT2, TUBB1, AKT3, AKT1, MAPK1, MAP2K3, HSP90AA1, MAP2K2, CHUK, RHOA, TUBA4A, NFKB1, PIK3CA, PKN1, RAF1, TLR4, TLR2	2.241217	1.55E-04
hsa05033:Nicotine addiction	GRIA1, GABRA1, GRIA2, GRIN2A, GABRA3, CACNA1B, GABRG2, GRIA3, GRIN1, GRIA4	4.900222	1.84E-04
hsa04724:Glutamatergic synapse	GRIA1, GRIA2, GRIK5, SLC1A1, SLC1A2, ITPR1, PRKCA, GRIK2, GRIN1, GRIN2A, GRM4, GRK2, GRM6, MAPK1, PRKACA, GRIA3, GRIA4	2.96996	2.12E-04
hsa04810:Regulation of actin cytoskeleton	ITGB1, CHRM1, ITGB3, LPAR1, LPAR2, CXCR4, PIK3CD, PIK3CB, PIK3R1, FGF1, FGF2, EGFR, SLC9A1, AKT2, AKT3, AKT1, MAPK1, PDGFRB, PDGFRA, MAP2K2, LIMK2, F2R, F2, RHOA, PIK3CA, RAF1	2.271146	2.28E-04
hsa04973:Carbohydrate digestion and absorption	AMY2A, PIK3CA, AKT2, AKT3, PIK3CD, AKT1, PIK3CB, SLC5A1, PIK3R1, SLC37A4, HK2	4.25	2.49E-04
hsa04630:JAK-STAT signaling pathway	PDGFRB, PDGFRA, STAT1, IL23R, STAT3, PIK3CD, PTPN11, PIK3CB, PIK3R1, EGFR, MTOR, PIK3CA, AKT2, AKT3, AKT1, EP300, GRB2, JAK2, RAF1, JAK3, PTPN2	2.511364	2.96E-04
hsa05131:Shigellosis	ITGB1, GSK3B, GSK3A, CXCL8, ITPR1, PIK3CD, PIK3CB, PIK3R1, HK2, EGFR, IKBKB, MAPK9, MAPK8, TBK1, AKT2,	2.178532	3.16E-04

	AKT3, AKT1, MAPK1, CAPN1, CHUK, PRKCD, RHOA, MTOR, NFKB1, STING1, PIK3CA, TLR4		
hsa04550:Signaling pathways regulating pluripotency of stem cells	GSK3B, MAP2K2, STAT3, PIK3CD, PIK3CB, PIK3R1, ACVR1B, FGF2, PIK3CA, AKT2, KAT6A, AKT3, AKT1, MAPK1, GRB2, JAK2, RAF1, JAK3, BMPR1A	2.650884	3.21E-04
hsa00790:Folate biosynthesis	QDPR, DHFR, AKR1B10, TH, PAH, AKR1C3, GGH, AKR1B1	5.952862	3.21E-04
hsa04929:GnRH secretion	MAP2K2, PIK3CA, AKT2, AKT3, ITPR1, MAPK1, PIK3CD, AKT1, PRKCA, PIK3CB, PIK3R1, RAF1	3.709091	3.77E-04
hsa05224:Breast cancer	GSK3B, MAP2K2, PIK3CD, PIK3CB, PIK3R1, FGF1, FGF2, ESR1, EGFR, MTOR, PIK3CA, CDK4, AKT2, AKT3, AKT1, MAPK1, GRB2, HES1, RAF1	2.579238	4.43E-04
hsa04621:NOD-like receptor signaling pathway	HSP90AA1, CXCL8, HSP90AB1, CHUK, STAT1, PRKCD, ITPR1, RHOA, NFKB1, P2RX7, IKBKB, MAPK9, MAPK8, CASP8, TBK1, STING1, NAMPT, MAPK1, PKN2, PKN1, TLR4, CTSB	2.338624	5.15E-04
hsa05032:Morphine addiction	GABRA1, PDE3B, GABRA3, CACNA1B, PRKCA, OPRM1, GABRG2, PDE11A, GRK2, GRK5, ADORA1, PDE4B, PDE7A, PRKACA	3.090909	6.07E-04
hsa04064:NF-kappa B signaling pathway	CXCL8, PARP1, CSNK2A1, CHUK, CSNK2A2, PTGS2, NFKB1, IKBKB, IRAK1, PLAU, LCK, CSNK2B, BTK, TLR4, MAP3K14	2.87013	7.59E-04
hsa05225:Hepatocellular carcinoma	GSK3B, MAP2K2, GSTP1, PIK3CD, KEAP1, PRKCA, PIK3CB, PIK3R1, EGFR, MTOR, TGFBR2, PIK3CA, CDK4, AKT2, AKT3, AKT1, MAPK1, GRB2, RAF1, NFE2L2	2.363636	8.66E-04
hsa00590:Arachidonic acid metabolism	CYP2B6, HPGD, TBXAS1, PLA2G2A, AKR1C3, ALOX12, LTA4H, PTGS2, ALOX15B, PTGES, PTGS1	3.622951	8.68E-04
hsa01232:Nucleotide metabolism	CDA, DTYMK, DUT, ADK, AMPD1, TYMS, TYMP, NT5E, PNP, TK1, HPRT1, XDH, ADA	3.072727	0.001064
hsa04666:Fc gamma R-mediated phagocytosis	SPHK2, SPHK1, LIMK2, PRKCD, PIK3CD, PRKCA, PIK3CB, PIK3R1, PIK3CA, AKT2, AKT3, AKT1, MAPK1, RAF1	2.87013	0.001192
hsa05130:Pathogenic Escherichia coli infection	ITGB1, CXCL8, CHUK, F2R, LPAR1, LPAR2, PTPN11, F2, RHOA, TUBA4A, NFKB1, IKBKB, MAPK9, MAPK8, CASP8, IRAK1, CASP3, TUBB1, ABL1, MAPK1, FYN, TLR4	2.17734	0.001237
hsa04723:Retrograde endocannabinoid signaling	GRIA1, GRIA2, GABRA1, FAAH, GABRA3, CACNA1B, ITPR1, PRKCA, PTGS2, GABRG2, MAPK9, MAPK8, CNR1, MAPK1, PRKACA, GRIA3, MGLL, GRIA4	2.42709	0.001237
hsa04022:cGMP-PKG signaling pathway	OPRD1, MAP2K2, NOS3, PDE3B, ITPR1, ADRB1, ADRB2, RHOA, PIK3CG, EDNRA, EDNRB, ADORA3, AKT2, AKT3, ADORA1, AKT1, MAPK1, PDE5A, RAF1	2.299562	0.001601
hsa00511:Other glycan degradation	GLB1, HEXB, FUCA1, HEXA, GBA1, GBA2	6.69697	0.001611

hsa04360:Axon guidance	ITGB1, GSK3B, CAMK2D, PDPK1, LIMK2, CXCR4, PIK3CD, PRKCA, PTPN11, PIK3CB, PIK3R1, PRKCZ, RHOA, PIK3CA, CDK5, ABL1, MAPK1, FYN, EPHB2, RAF1	2.183794	0.002094
hsa00240:Pyrimidine metabolism	CDA, DTYMK, NT5E, DUT, CAD, TK1, TYMS, UMPS, TYMP, DHODH	3.46395	0.002212
hsa00983:Drug metabolism - other enzymes	CDA, CYP2A6, DUT, GSTP1, HPRT1, TK1, CYP3A4, UMPS, GUSB, XDH, TYMP, CES1	2.976431	0.002225
hsa04960:Aldosterone-regulated sodium reabsorption	PIK3CA, PDPK1, MAPK1, PIK3CD, PRKCA, PIK3CB, PIK3R1, NR3C2	4.229665	0.002485
hsa05120:Epithelial cell signaling in Helicobacter pylori infection	IKBKB, MAPK9, MAPK8, CXCL8, CHUK, CASP3, ADAM10, PTPN11, MAP3K14, EGFR, NFKB1	3.112676	0.002626
hsa00500:Starch and sucrose metabolism	GPI, GYS1, PYGB, AMY2A, AGL, GAA, PYGM, HK2	4.018182	0.003321
hsa04976:Bile secretion	RXRA, EPHX1, SLC2A1, NR1H4, HMGCR, SLC5A1, CYP3A4, PRKACA, NR0B2, CFTR, SLC9A1, ABCG2	2.678788	0.004974
hsa05146:Amoebiasis	CXCL8, NOS2, PIK3CD, PRKCA, PIK3CB, PIK3R1, NFKB1, PIK3CA, CASP3, CTSG, PRKACA, TLR4, TLR2	2.535746	0.005018
hsa05133:Pertussis	ITGB1, MAPK9, MAPK8, CXCL8, IRAK1, NOS2, CASP3, MAPK1, TLR4, RHOA, NFKB1	2.833333	0.005109
hsa04110:Cell cycle	GSK3B, HDAC2, HDAC1, PLK1, CDC7, PKMYT1, CDC25C, HDAC8, AURKB, CDC25B, CCNE1, CDK4, CHEK2, CDK2, CDK1, ABL1, EP300	2.16168	0.005246
hsa04142:Lysosome	HEXB, GAA, FUCA1, HEXA, GBA1, CTSS, IGF2R, NPC1, GLB1, CTSL, CTSG, GUSB, CTSD, GLA, CTSB	2.283058	0.005812
hsa04924:Renin secretion	EDNRA, ACE, PTGER2, PDE3B, ADORA1, ITPR1, ADRB1, ADRB2, PRKACA, CTSB	2.911726	0.006776
hsa00360:Phenylalanine metabolism	AOC3, MAOB, MAOA, PAH, MIF	6.278409	0.006777
hsa04622:RIG-I-like receptor signaling pathway	IKBKB, MAPK9, MAPK8, TBK1, CXCL8, CASP8, STING1, CHUK, PIN1, NFKB1	2.790404	0.008933
hsa05216:Thyroid cancer	RXRB, NTRK1, RET, RXRA, MAP2K2, MAPK1, PPARG	3.800983	0.009086
hsa04921:Oxytocin signaling pathway	CAMK2D, PRKAA1, PRKAB2, MAP2K2, NOS3, ITPR1, PRKCA, PTGS2, RHOA, EGFR, PIK3CG, CAMKK2, CD38, MAPK1, RAF1, PRKACA	2.087367	0.009451
hsa00760:Nicotinate and nicotinamide metabolism	NT5E, PNP, NAMPT, CD38, SIRT1, SIRT2, SIRT3	3.700957	0.010349
hsa05020:Prion disease	GSK3B, CSNK2A1, PRKCD, CSNK2A2, CACNA1B, ITPR1, PIK3CD, PIK3CB, PIK3R1, TUBA4A, GRIN1, MAPK9, GRIN2A, MAPK8, PSMB5, PIK3CA, PSMB2, CASP3, TUBB1, CSNK2B, PSMB1, MAPK1, FYN, PRKACA	1.734467	0.011079
hsa04650:Natural killer cell mediated cytotoxicity	MAP2K2, PIK3CD, PRKCA, PTPN11, PIK3CB, PIK3R1, PIK3CA, LCK, CASP3, PTK2B, MAPK1, GRB2, FYN, RAF1	2.163636	0.012306

hsa04670:Leukocyte transendothelial migration	ITGB1, ITK, MMP2, CXCR4, PIK3CD, PRKCA, PTPN11, PIK3CB, PIK3R1, MMP9, RHOA, PIK3CA, PTK2B	2.251567	0.012321
hsa04217:Necroptosis	PYGB, CAMK2D, HSP90AA1, HSP90AB1, PARP1, STAT1, STAT3, PYGM, MAPK9, MAPK8, CASP8, CAPN1, JAK2, PPIA, JAK3, TLR4	2.021727	0.012475
hsa00531:Glycosaminoglycan degradation	GLB1, HEXB, HEXA, HPSE, GUSB	5.287081	0.012828
hsa05140:Leishmaniasis	ITGB1, IRAK1, NOS2, STAT1, MAPK1, JAK2, PTGS2, TLR4, NFKB1, TLR2	2.609209	0.013631
hsa00600:Sphingolipid metabolism	SPHK2, GLB1, HEXB, SPHK1, HEXA, GBA1, GBA2, GLA	2.976431	0.016691
hsa00052:Galactose metabolism	AKR1B10, GLB1, GAA, AKR1B1, GLA, HK2	3.767045	0.019608
hsa05016:Huntington disease	GRIA1, GRIA2, HDAC2, HDAC1, CACNA1B, SLC1A2, ITPR1, MAPK9, MAPK8, CASP8, PSMB5, PSMB2, CASP3, TUBB1, PSMB1, EP300, GRIA3, TGM2, GRIA4, MAP3K5, MTOR, TUBA4A, GRIN1, ULK1, PPARG	1.615025	0.020939
hsa04713:Circadian entrainment	GRIA1, GRIA2, CAMK2D, GRIN2A, ITPR1, MAPK1, PRKCA, PRKACA, GRIA3, GRIN1, GRIA4	2.278351	0.021784
hsa04934:Cushing syndrome	GSK3B, CAMK2D, FH, MAP2K2, ITPR1, EGFR, NR4A1, PDE11A, CCNE1, CDK4, CYP11B1, CDK2, WDR5, MAPK1, PRKACA	1.944282	0.021816
hsa00051:Fructose and mannose metabolism	AKR1B10, TPI1, AKR1B1, ALDOA, FBP1, HK2	3.545455	0.024996
hsa00900:Terpenoid backbone biosynthesis	ICMT, FNTA, FNTB, MVD, HMGCR	4.367589	0.025073
hsa04928:Parathyroid hormone synthesis, secretion and action	RXRB, MMP14, RXRA, VDR, PDE4B, ITPR1, MAPK1, PRKCA, RAF1, PRKACA, RHOA, EGFR	2.096443	0.027332
hsa04916:Melanogenesis	GSK3B, CAMK2D, MAP2K2, EDNRB, MC1R, EP300, MAPK1, PRKCA, TYR, RAF1, PRKACA	2.188119	0.027972
hsa04730:Long-term depression	GRIA1, GRIA2, MAP2K2, ITPR1, MAPK1, PRKCA, RAF1, GRIA3	2.678788	0.028285
hsa05202:Transcriptional misregulation in cancer	NTRK1, HDAC2, FLT1, CXCL8, HPGD, HDAC1, MMP3, PLAT, MMP9, NFKB1, TGFBR2, RXRB, RXRA, PLAU, BMP2K, PPARG, GRIA3	1.769666	0.030467
hsa00350:Tyrosine metabolism	AOC3, MAOB, TH, MAOA, MIF, TYR	3.348485	0.031251
hsa04115:p53 signaling pathway	CASP8, CCNE1, CDK4, CHEK2, CASP3, CDK2, SERPINE1, CDK1, MDM4	2.410909	0.031867
hsa00140:Steroid hormone biosynthesis	CYP11B1, AKR1C3, HSD17B3, CYP1B1, AKR1C2, AKR1C4, CYP3A4, CYP19A1	2.592375	0.033125
hsa03320:PPAR signaling pathway	RXRB, RXRA, FABP4, PDPK1, MMP1, SCD, PPARG, CD36, PPARA	2.379187	0.034137
hsa00604:Glycosphingolipid biosynthesis - ganglio series	GLB1, HEXB, HEXA, ST3GAL1	5.357576	0.035442
hsa00330:Arginine and proline metabolism	MAOB, ALDH2, P4HA1, NOS2, MAOA, NOS3, LAP3	2.812727	0.036282

hsa04658:Th1 and Th2 cell differentiation	IKBKB, MAPK9, MAPK8, CHUK, STAT1, LCK, MAPK1, JAK2, JAK3, NFKB1	2.183794	0.038487
hsa04520:Adherens junction	PTPN1, CSNK2A1, CSNK2A2, CSNK2B, EP300, MAPK1, FYN, RHOA, EGFR, TGFBR2	2.160313	0.040826
hsa00603:Glycosphingolipid biosynthesis - globo and isoglobo series	HEXB, HEXA, ST3GAL1, GLA	5.022727	0.042058
hsa00270:Cysteine and methionine metabolism	LDHB, LDHA, GCLC, DNMT1, BHMT, AHCY, BCAT2	2.704545	0.042827
hsa04114:Oocyte meiosis	CAMK2D, PLK1, ITPR1, PKMYT1, CDC25C, AR, RPS6KA3, CCNE1, RPS6KA1, CDK2, CDK1, MAPK1, PRKACA	1.879006	0.043363
hsa00010:Glycolysis / Gluconeogenesis	GPI, LDHB, LDHA, TPI1, ALDH2, ALDOA, FBP1, HK2	2.398915	0.04755
hsa05012:Parkinson disease	CAMK2D, MAOB, MAOA, ITPR1, KEAP1, SLC6A3, TUBA4A, MAPK9, MAPK8, PSMB5, ADORA2A, TH, PSMB2, CASP3, TUBB1, PSMB1, MAPT, DRD2, PRKACA, NFE2L2, MAP3K5	1.55686	0.04983

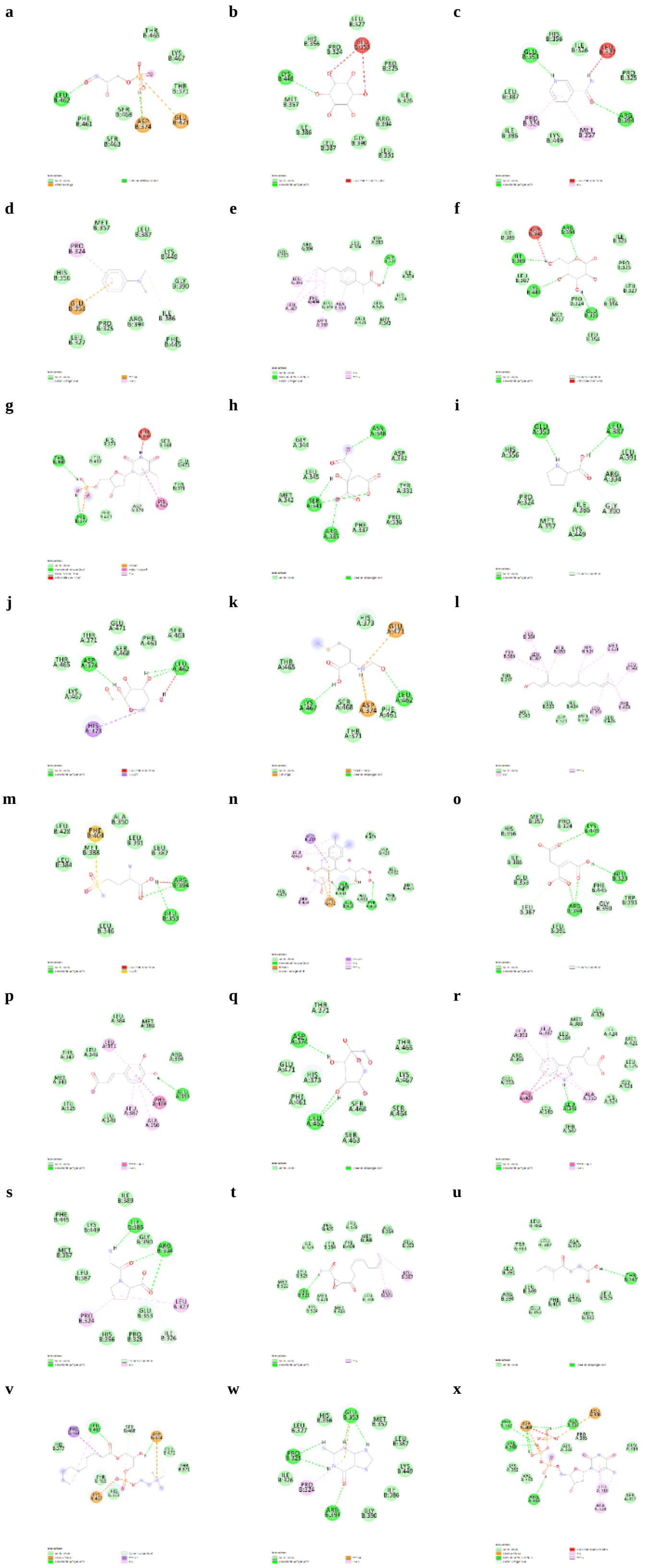

S27 Fig. Two-dimensional visualization of interactions between ESR1 (PDB ID: 1A52) and **a.** 729. b. 892. **c.** 936. d. 949. **e.** 3672. **f.** 6036. **g.** 9700. **h.** 31348. **i.** 145742. **j.** 439192. **k.** 439750. **l.** 445070. **m.** 44582. **n.** 493570. **o.** 643757. **p.** 689043. **q.** 2723872. **r.** 2761549. **s.** 3013625. **t.** 5282054. **u.** 6441567. **v.** 22851442. **w.** 135398634.

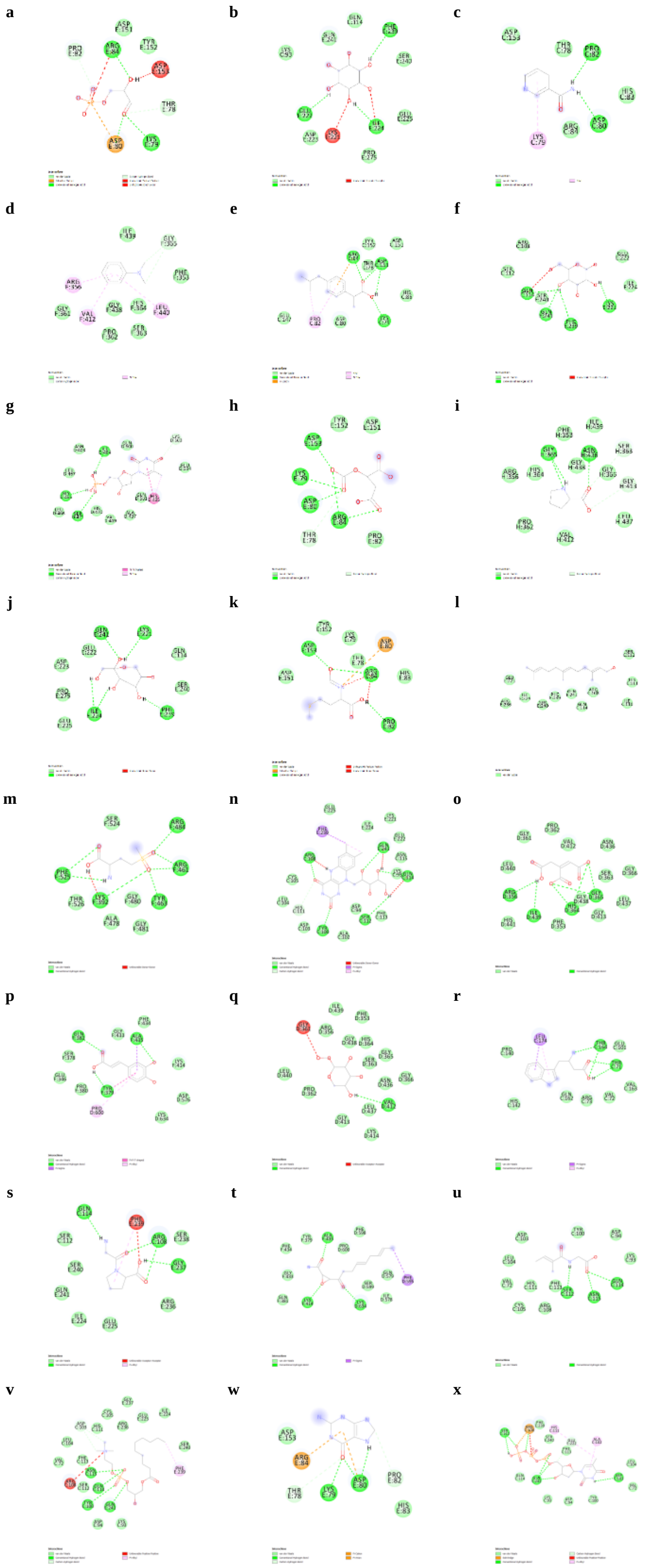

S28 Fig. Two-dimensional visualization of interactions between NFKB1 (PDB ID: 3GUT) and **a.** 729. b. 892. **c.** 936. d. 949. **e.** 3672. **f.** 6036. **g.** 9700. **h.** 31348. **i.** 145742. **j.** 439192. **k.** 439750. **l.** 445070. **m.** 44582. **n.** 493570. **o.** 643757. **p.** 689043. **q.** 2723872. **r.** 2761549. **s.** 3013625. **t.** 5282054. **u.** 6441567. **v.** 22851442. **w.** 135398634.

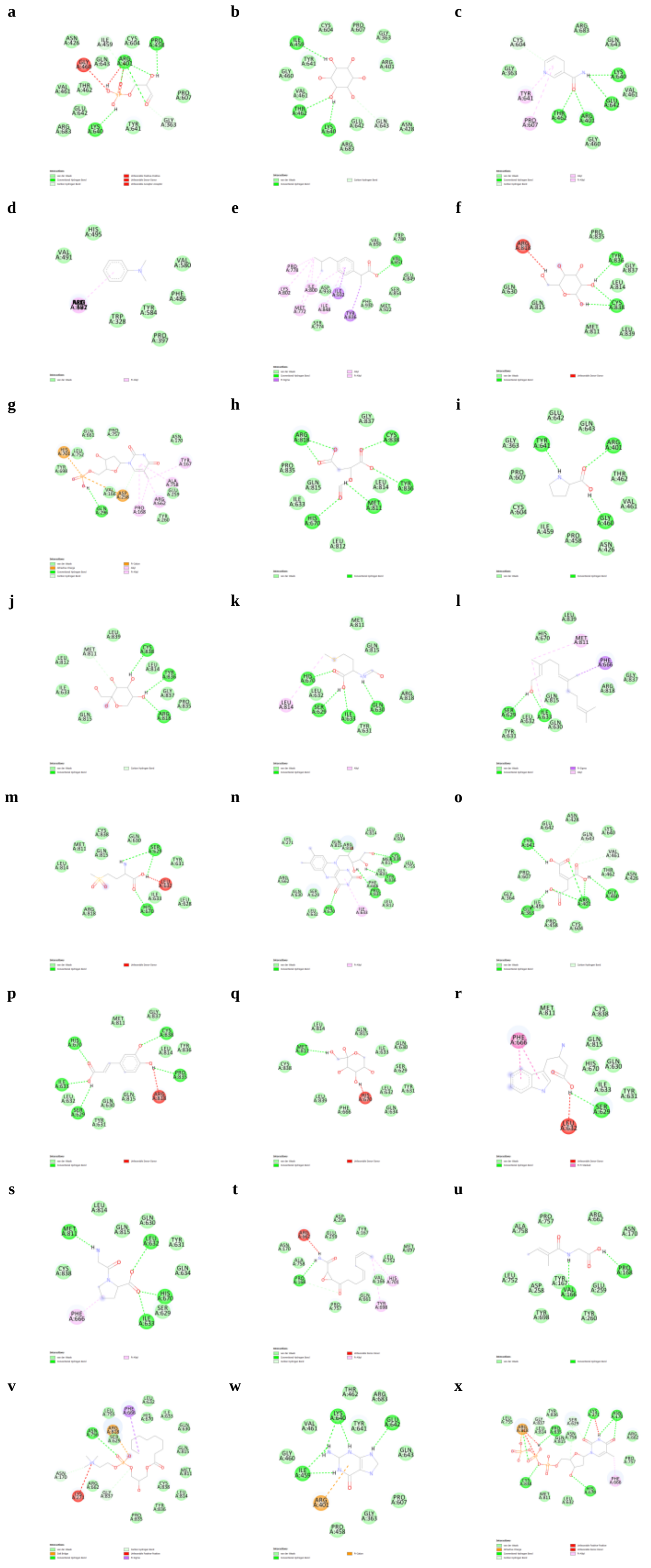

S29 Fig. Two-dimensional visualization of interactions between PIK3CA (PDB ID: 7R9V) and **a.** 729. b. 892. **c.** 936. d. 949. **e.** 3672. **f.** 6036. **g.** 9700. **h.** 31348. **i.** 145742. **j.** 439192. **k.** 439750. **l.** 445070. **m.** 44582. **n.** 493570. **o.** 643757. **p.** 689043. **q.** 2723872. **r.** 2761549. **s.** 3013625. **t.** 5282054. **u.** 6441567. **v.** 22851442. **w.** 135398634.

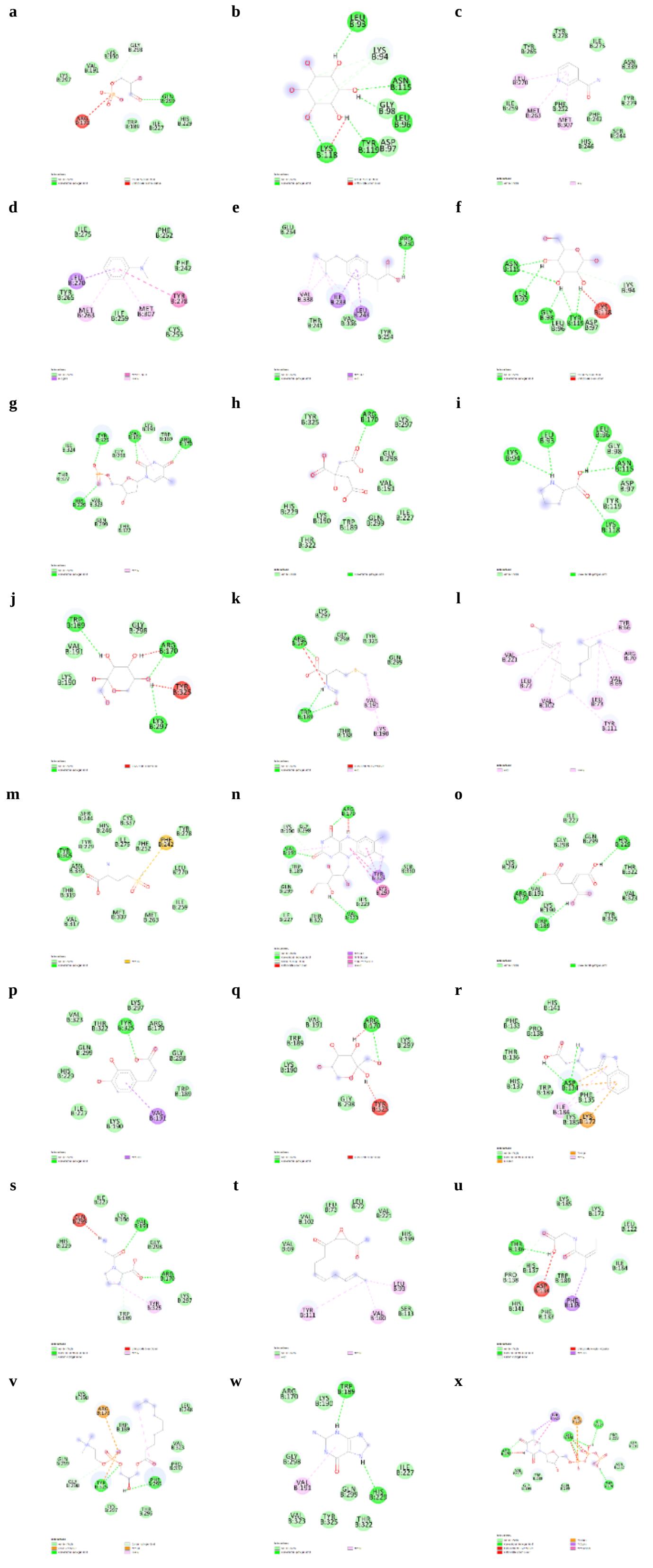

S30 Fig. Two-dimensional visualization of interactions between HIF1A (PDB ID: 4ZPR) and **a.** 729. b. 892. **c.** 936. d. 949. **e.** 3672. **f.** 6036. **g.** 9700. **h.** 31348. **i.** 145742. **j.** 439192. **k.** 439750. **l.** 445070. **m.** 44582. **n.** 493570. **o.** 643757. **p.** 689043. **q.** 2723872. **r.** 2761549. **s.** 3013625. **t.** 5282054. **u.** 6441567. **v.** 22851442. **w.** 135398634.

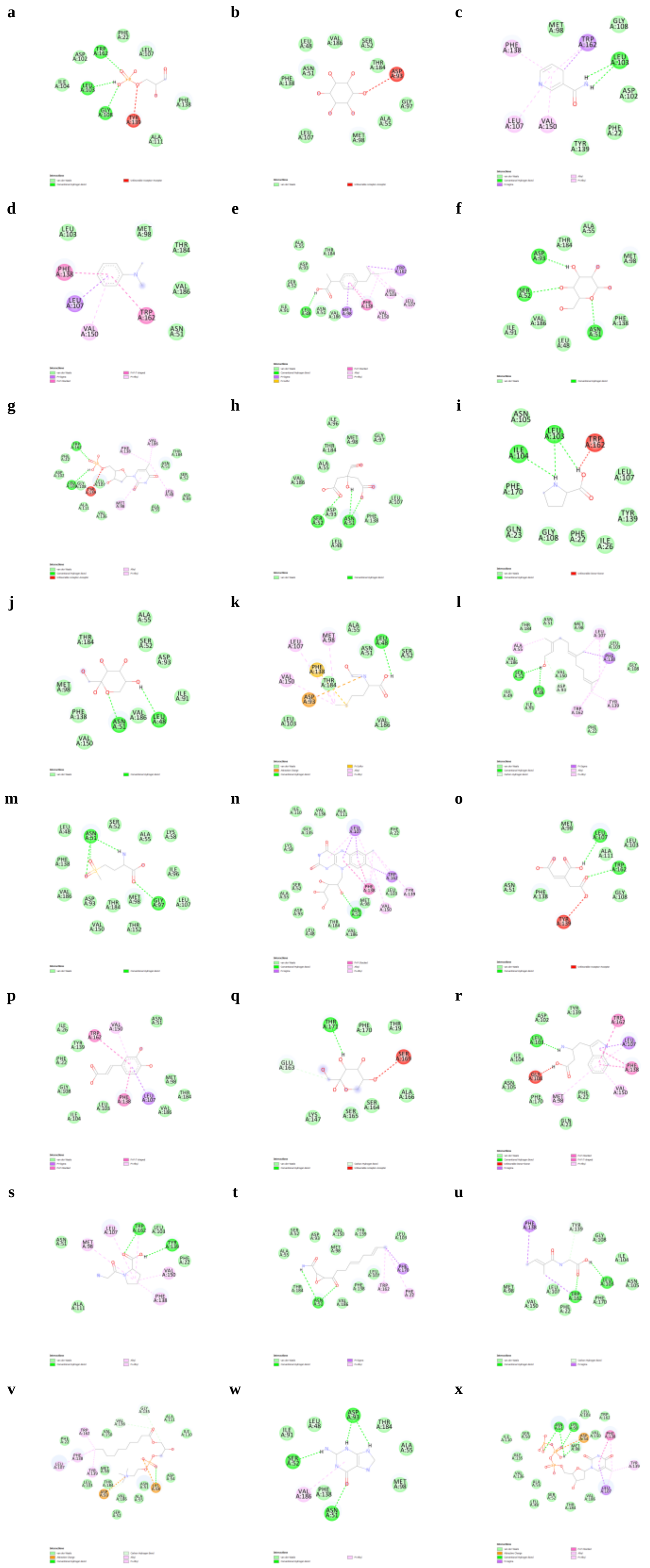

S31 Fig. Two-dimensional visualization of interactions between HSP90AA1 (PDB ID: 4BQG) and **a.** 729. b. 892. **c.** 936. d. 949. **e.** 3672. **f.** 6036. **g.** 9700. **h.** 31348. **i.** 145742. **j.** 439192. **k.** 439750. **l.** 445070. **m.** 44582. **n.** 493570. **o.** 643757. **p.** 689043. **q.** 2723872. **r.** 2761549. **s.** 3013625. **t.** 5282054. **u.** 6441567. **v.** 22851442. **w.** 135398634.

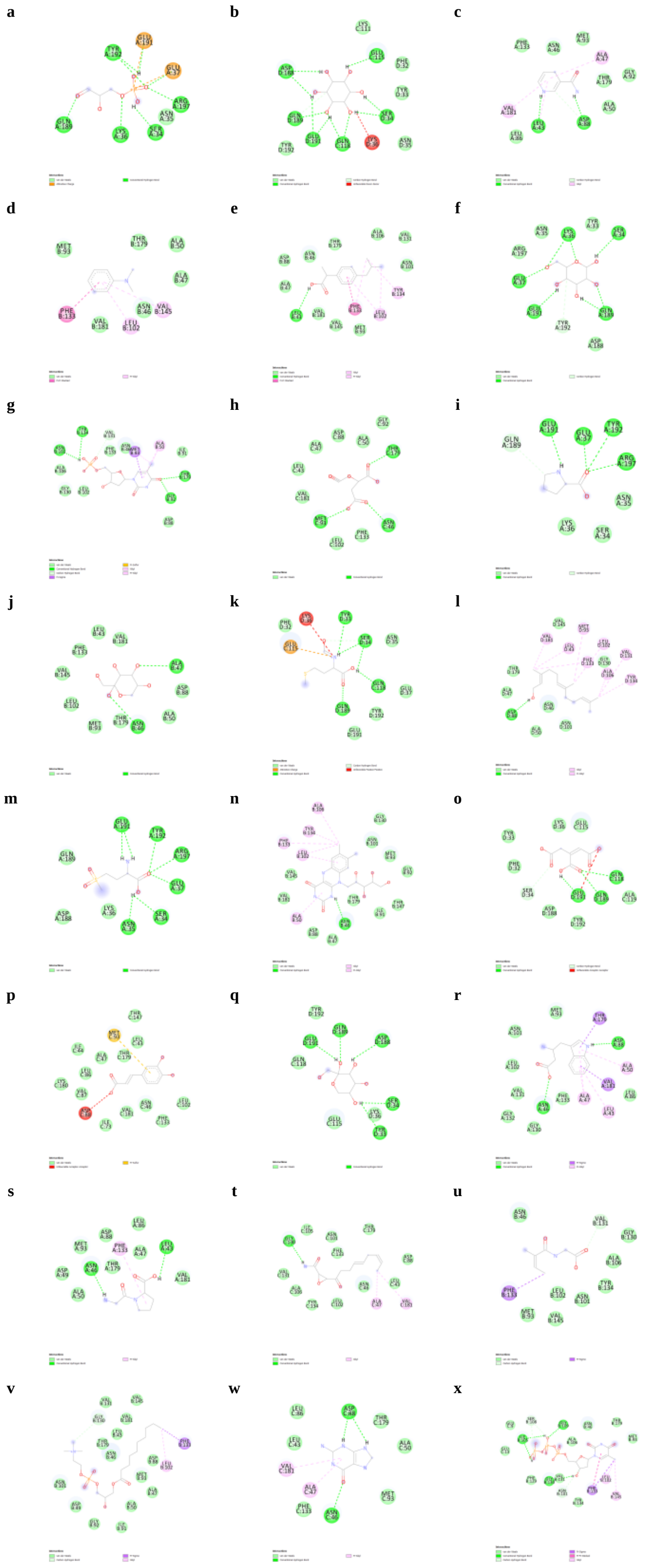

S32 Fig. Two-dimensional visualization of interactions between HSP90AB1 (PDB ID: 5UC4) and **a.** 729. b. 892. **c.** 936. d. 949. **e.** 3672. **f.** 6036. **g.** 9700. **h.** 31348. **i.** 145742. **j.** 439192. **k.** 439750. **l.** 445070. **m.** 44582. **n.** 493570. **o.** 643757. **p.** 689043. **q.** 2723872. **r.** 2761549. **s.** 3013625. **t.** 5282054. **u.** 6441567. **v.** 22851442. **w.** 135398634.

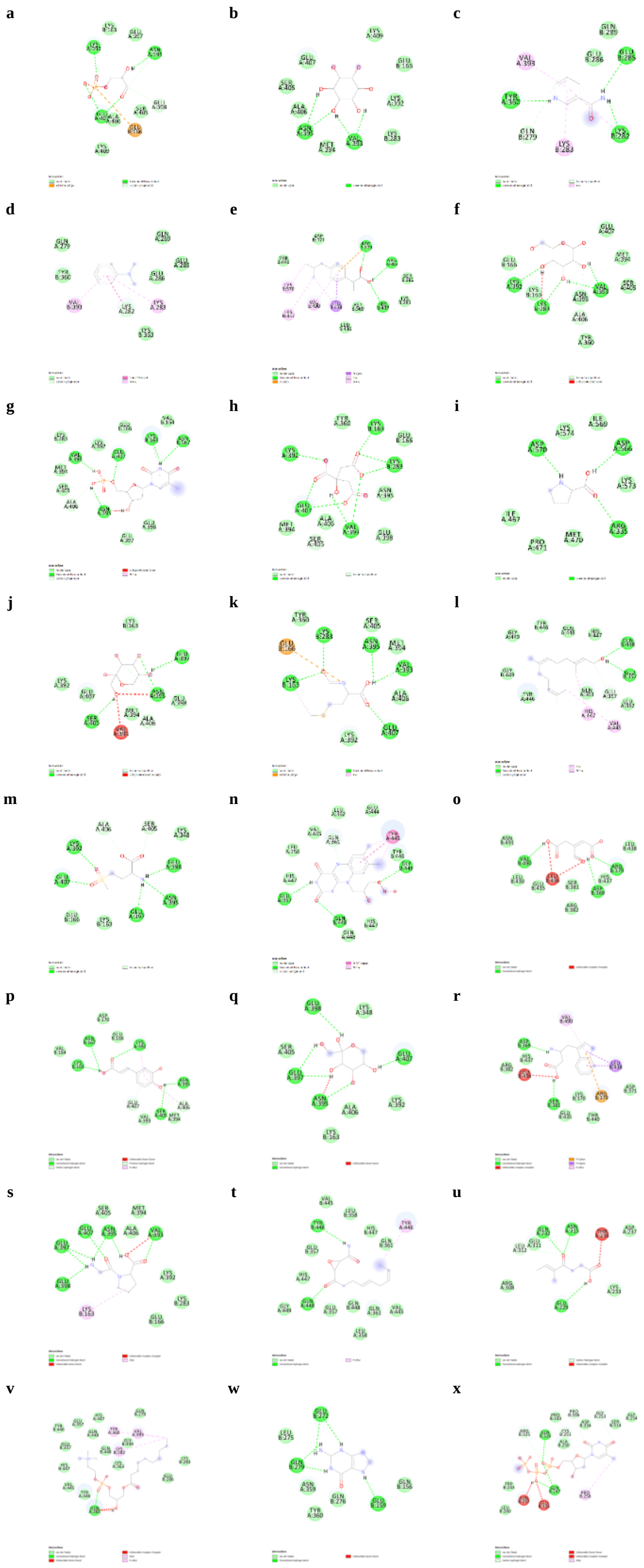

S33 Fig. Two-dimensional visualization of interactions between STAT3 (PDB ID: 6TLC) and **a.** 729. b. 892. **c.** 936. d. 949. **e.** 3672. **f.** 6036. **g.** 9700. **h.** 31348. **i.** 145742. **j.** 439192. **k.** 439750. **l.** 445070. **m.** 44582. **n.** 493570. **o.** 643757. **p.** 689043. **q.** 2723872. **r.** 2761549. **s.** 3013625. **t.** 5282054. **u.** 6441567. **v.** 22851442. **w.** 135398634.

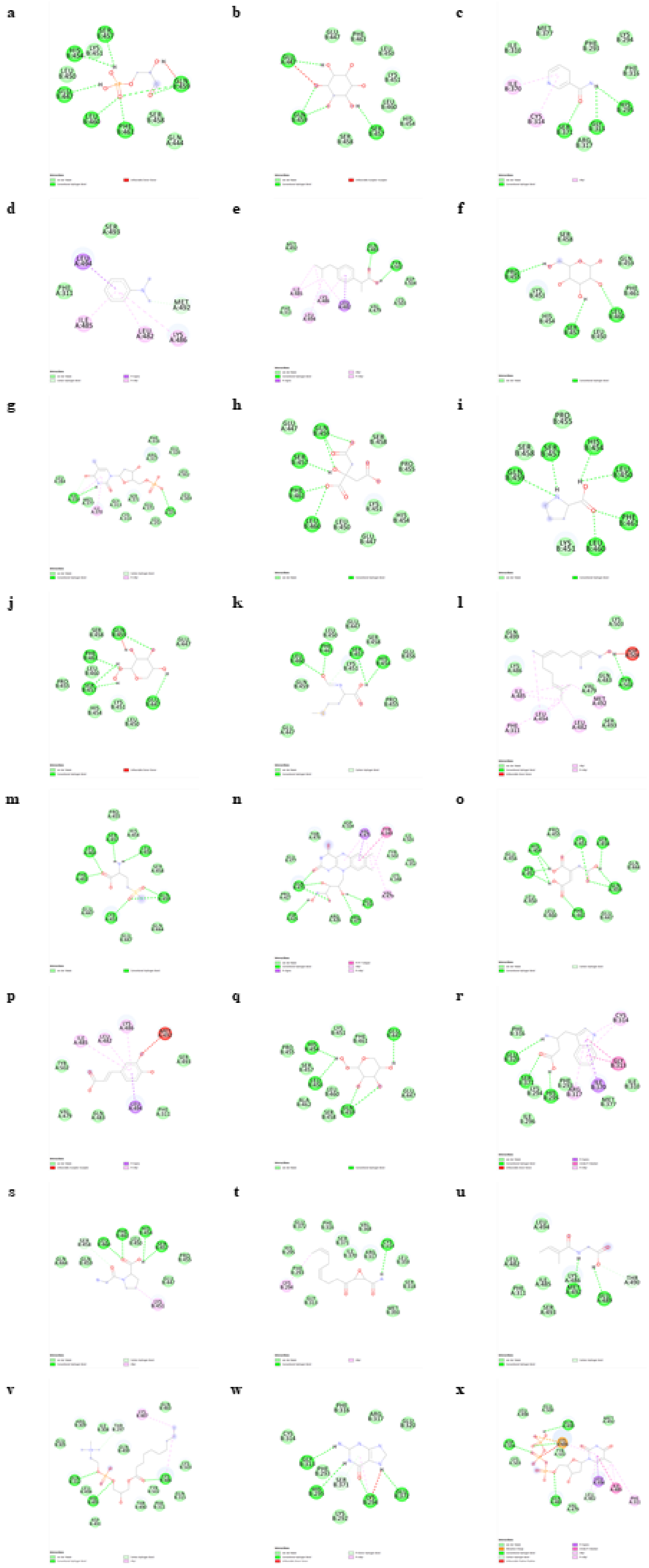

S34 Fig. Two-dimensional visualization of interactions between PPARG (PDB ID: 8WFE) and **a.** 729. b. 892. **c.** 936. d. 949. **e.** 3672. **f.** 6036. **g.** 9700. **h.** 31348. **i.** 145742. **j.** 439192. **k.** 439750. **l.** 445070. **m.** 44582. **n.** 493570. **o.** 643757. **p.** 689043. **q.** 2723872. **r.** 2761549. **s.** 3013625. **t.** 5282054. **u.** 6441567. **v.** 22851442. **w.** 135398634.

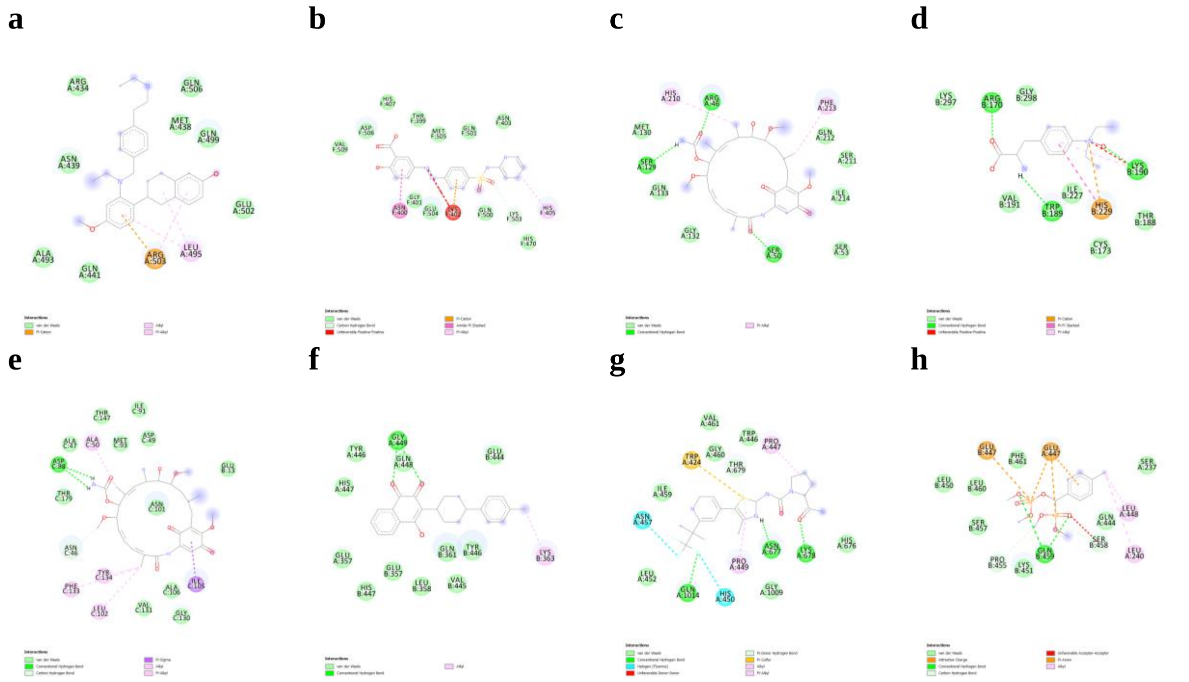

S35 Fig. Two-dimensional visualization of interactions between the studied core targets and their FDA-approved specific inhibitors. **a.** 1A52-23642301. **b.** 3GUT-5339. **c.** 4BQG-5288382. **d.** 4ZPR-11234794. **e.** 5UC4-5288382. **f.** 6TLC-74989. **g.** 7R9V-56649450. **h.** 8WFE-60910.

S3 Table. Effect of *S. mannii* roots aqueous extract on gene expression (raw data, $2^{-\Delta\Delta Ct}$ values)

Genes		1/4 IC50, 18H					1/2 IC50, 24H				
		Rep 1	Rep 2	Rep 3	Mean	SD	Rep 1	Rep 2	Rep 3	Mean	SD
NFKB1	Untreated	1.00	1.00	1.00	1.00	0.00	1.00	1.00	1.00	1.00	0.00
	RTAq	0.47	0.41	0.48	0.45	0.03	0.24	0.23	0.20	0.22	0.02
	Rapamycin	0.39	0.34	0.42	0.38	0.04	0.45	0.50	0.48	0.48	0.02
PIK3CA	Untreated	1.00	1.00	1.00	1.00	0.00	1.00	1.00	1.00	1.00	0.00
	RTAq	0.46	0.39	0.43	0.43	0.04	1.81	1.77	1.74	1.77	0.03
	Rapamycin	0.64	0.72	0.67	0.68	0.04	5.49	5.76	6.01	5.75	0.26
HIF1A	Untreated	1.00	1.00	1.00	1.00	0.00	1.00	1.00	1.00	1.00	0.00
	RTAq	0.55	0.49	0.50	0.51	0.03	0.15	0.04	0.09	0.10	0.05
	Rapamycin	0.18	0.13	0.17	0.16	0.03	6.62	7.11	6.77	6.83	0.25
STAT3	Untreated	1.00	1.00	1.00	1.00	0.00	1.00	1.00	1.00	1.00	0.00
	RTAq	2.21	2.31	2.28	2.27	0.05	2.27	2.58	2.32	2.39	0.17
	Rapamycin	0.35	0.27	0.38	0.33	0.06	2.36	2.20	2.31	2.29	0.08
HSP90AA1	Untreated	1.00	1.00	1.00	1.00	0.00	1.00	1.00	1.00	1.00	0.00
	RTAq	0.43	0.42	0.43	0.43	0.01	2.59	2.57	2.31	2.49	0.16
	Rapamycin	0.36	0.39	0.47	0.40	0.06	3.05	3.22	3.11	3.13	0.09
HSP90AB1	Untreated	1.00	1.00	1.00	1.00	0.00	1.00	1.00	1.00	1.00	0.00
	RTAq	0.63	0.57	0.65	0.62	0.04	2.42	2.37	2.39	2.39	0.02
	Rapamycin	0.74	0.73	0.75	0.74	0.01	2.50	2.52	2.58	2.53	0.04
PPARG	Untreated	1.00	1.00	1.00	1.00	0.00	1.00	1.00	1.00	1.00	0.00
	RTAq	0.53	0.47	0.51	0.50	0.03	0.69	0.66	0.70	0.68	0.02
	Rapamycin	1.41	1.43	1.40	1.41	0.01	3.32	3.01	3.12	3.15	0.16
ESR1	Untreated	1.00	1.00	1.00	1.00	0.00	1.00	1.00	1.00	1.00	0.00
	RTAq	0.74	0.79	0.70	0.74	0.04	0.30	0.27	0.38	0.31	0.06
	Rapamycin	0.80	0.86	0.82	0.83	0.03	1.66	1.57	1.51	1.58	0.07
BAK	Untreated	1.00	1.00	1.00	1.00	0.00	1.00	1.00	1.00	1.00	0.00
	RTAq	0.22	0.30	0.27	0.26	0.04	0.60	0.55	0.58	0.58	0.03
	Rapamycin	0.94	1.34	0.97	1.08	0.23	3.47	3.59	3.30	3.45	0.15
BAX	Untreated	1.00	1.00	1.00	1.00	0.00	1.00	1.00	1.00	1.00	0.00
	RTAq	0.24	0.17	0.30	0.24	0.06	0.24	0.31	0.58	0.38	0.18
	Rapamycin	0.17	0.13	0.20	0.17	0.04	1.01	0.97	1.11	1.03	0.07
NOXA	Untreated	1.00	1.00	1.00	1.00	0.00	1.00	1.00	1.00	1.00	0.00
	RTAq	0.47	0.44	0.47	0.46	0.02	0.94	0.99	0.98	0.97	0.03
	Rapamycin	0.27	0.29	0.20	0.25	0.05	0.70	0.64	0.71	0.68	0.04
PUMA	Untreated	1.00	1.00	1.00	1.00	0.00	1.00	1.00	1.00	1.00	0.00
	RTAq	1.15	1.01	1.23	1.13	0.11	0.62	0.66	0.60	0.63	0.03
	Rapamycin	0.69	0.70	0.69	0.69	0.01	4.66	4.12	4.55	4.44	0.29
cEBP	Untreated	1.00	1.00	1.00	1.00	0.00	1.00	1.00	1.00	1.00	0.00

	RTAq	0.72	0.75	0.69	0.72	0.03	3.29	3.43	3.44	3.39	0.09
	Rapamycin	0.55	0.32	0.75	0.54	0.21	1.79	1.87	2.09	1.92	0.16
FASL	Untreated	1.00	1.00	1.00	1.00	0.00	1.00	1.00	1.00	1.00	0.00
	RTAq	0.56	0.47	0.61	0.54	0.07	0.20	0.18	0.21	0.20	0.01
	Rapamycin	0.03	0.03	0.02	0.02	0.01	0.83	0.75	0.85	0.81	0.05
APAF1	Untreated	1.00	1.00	1.00	1.00	0.00	1.00	1.00	1.00	1.00	0.00
	RTAq	3.14	3.13	3.10	3.12	0.02	1.03	0.98	1.00	1.01	0.02
	Rapamycin	1.79	1.66	1.71	1.72	0.06	4.37	4.12	4.32	4.27	0.13
P53	Untreated	1.00	1.00	1.00	1.00	0.00	1.00	1.00	1.00	1.00	0.00
	RTAq	0.83	0.76	0.81	0.80	0.03	3.96	4.02	3.98	3.99	0.03
	Rapamycin	1.01	0.98	1.02	1.00	0.02	4.73	4.78	4.74	4.75	0.03
ATG13	Untreated	1.00	1.00	1.00	1.00	0.00	1.00	1.00	1.00	1.00	0.00
	RTAq	0.19	0.22	0.20	0.21	0.02	0.88	0.76	0.91	0.85	0.08
	Rapamycin	0.28	0.21	0.31	0.26	0.05	0.82	0.81	0.80	0.81	0.01
Beclin 1	Untreated	1.00	1.00	1.00	1.00	0.00	1.00	1.00	1.00	1.00	0.00
	RTAq	0.44	0.41	0.42	0.42	0.02	0.87	0.73	0.80	0.80	0.07
	Rapamycin	0.39	0.37	0.40	0.39	0.02	1.03	1.04	1.00	1.02	0.02
LC3	Untreated	1.00	1.00	1.00	1.00	0.00	1.00	1.00	1.00	1.00	0.00
	RTAq	0.31	0.33	0.28	0.30	0.03	0.83	0.76	0.86	0.82	0.05
	Rapamycin	0.47	0.49	0.40	0.45	0.05	2.46	2.57	2.38	2.47	0.10
E6	Untreated	1.00	1.00	1.00	1.00	0.00	1.00	1.00	1.00	1.00	0.00
	RTAq	0.25	0.23	0.31	0.26	0.04	1.34	1.03	1.12	1.16	0.16
	Rapamycin	0.53	0.67	0.54	0.58	0.08	7.93	8.25	7.70	7.96	0.27
E7	Untreated	1.00	1.00	1.00	1.00	0.00	1.00	1.00	1.00	1.00	0.00
	RTAq	15.60	15.54	15.62	15.59	0.04	22.61	22.46	22.70	22.59	0.12
	Rapamycin	1.22	1.09	1.27	1.19	0.09	37.75	37.01	37.81	37.53	0.44
RB	Untreated	1.00	1.00	1.00	1.00	0.00	1.00	1.00	1.00	1.00	0.00
	RTAq	0.54	0.50	0.43	0.49	0.06	1.99	2.00	1.76	1.92	0.14
	Rapamycin	1.03	0.91	1.28	1.07	0.19	5.01	5.13	4.86	5.00	0.14
P16	Untreated	1.00	1.00	1.00	1.00	0.00	1.00	1.00	1.00	1.00	0.00
	RTAq	0.15	0.11	0.16	0.14	0.02	0.25	0.28	0.22	0.25	0.03
	Rapamycin	0.16	0.15	0.18	0.16	0.02	1.13	1.14	1.12	1.13	0.01
P21	Untreated	1.00	1.00	1.00	1.00	0.00	1.00	1.00	1.00	1.00	0.00
	RTAq	0.04	0.01	0.04	0.03	0.02	0.01	0.00	0.01	0.01	0.00
	Rapamycin	0.05	0.01	0.03	0.03	0.02	0.14	0.18	0.13	0.15	0.03

S4 Table. MDC staining intensity (raw data)

	1/4 IC50, 18H					1/2 IC50, 24H				
	Rep 1	Rep 2	Rep 3	Mean	SD	Rep 1	Rep 2	Rep 3	Mean	SD
Untreated	14.86	15.07	14.59	14.84	0.24	14.86	15.07	14.59	14.84	0.24
RTAq	10.42	16.21	15.89	14.17	3.25	18.22	19.83	22.02	20.02	1.91
Rapamycin	12.42	13.79	12.95	13.05	0.69	10.27	12.80	7.83	10.30	2.48

S5 Table. Effect of *S. mannii* roots aqueous extract on cell cycle (raw data)

		1/4 IC50, 18H					1/2 IC50, 24H				
		Rep 1	Rep 2	Rep 3	Mean	SD	Rep 1	Rep 2	Rep 3	Mean	SD
RTAq	G1	13.60	13.30	10.70	12.53	1.30	27.10	26.50	31.20	28.27	2.09
	S	1.24	2.11	1.86	1.74	0.37	18.40	17.50	11.70	15.87	2.97
	G2	84.90	84.50	87.10	85.50	1.14	55.20	56.60	68.70	60.17	6.06
Rapamycin	G1	12.90	13.90	10.80	12.53	1.58	15.60	16.00	11.50	14.37	2.49
	S	7.56	6.97	7.91	7.48	0.48	6.92	6.84	9.20	7.65	1.34
	G2	79.60	79.40	82.01	80.34	1.45	77.50	77.30	79.50	78.10	1.22
Untreated	G1	51.10	62.70	49.20	54.33	7.31	51.10	62.70	49.20	54.33	7.31
	S	27.10	29.80	18.10	25.00	6.13	27.10	29.80	18.10	25.00	6.13
	G2	21.20	7.20	31.90	20.10	12.39	21.20	7.20	31.90	20.10	12.39
